# Dynamic DNA Networks-Guided Directional and Orthogonal Transient Biocatalytic Cascades

**DOI:** 10.1021/jacs.3c08020

**Published:** 2023-09-29

**Authors:** Yu Ouyang, Jiantong Dong, Itamar Willner

**Affiliations:** The Institute of Chemistry, Center for Nanoscience and Nanotechnology, The Hebrew University of Jerusalem, Jerusalem 91904, Israel

## Abstract

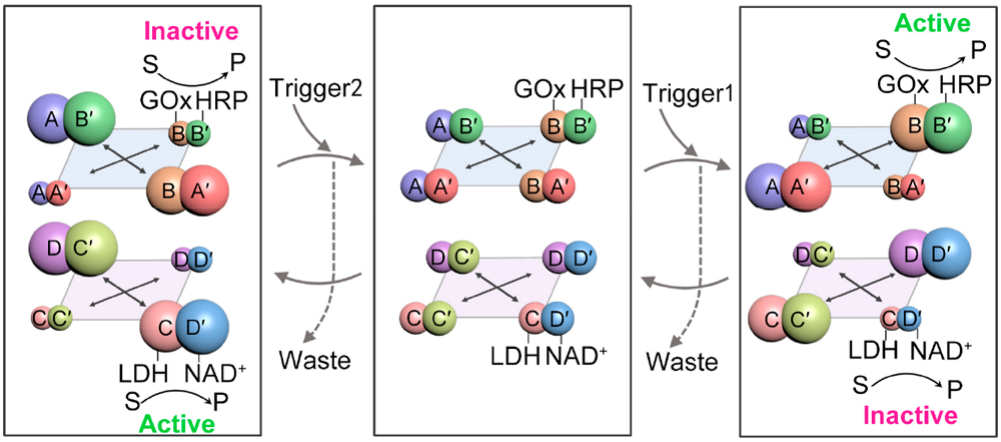

DNA frameworks, consisting of constitutional dynamic networks (CDNs) undergoing fuel-driven reconfiguration, are coupled to a dissipative reaction module that triggers the reconfigured CDNs into a transient intermediate CDNs recovering the parent CDN state. Biocatalytic cascades consisting of the glucose oxidase (GOx)/horseradish peroxidase (HRP) couple or the lactate dehydrogenase (LDH)/nicotinamide adenine dinucleotide (NAD^+^) couple are tethered to the constituents of two different CDNs, allowing the CDNs-guided operation of the spatially confined GOx/HRP or LDH/NAD^+^ biocatalytic cascades. By applying two different fuel triggers, the directional transient CDN-guided upregulation/downregulation of the two biocatalytic cascades are demonstrated. By mixing the GOx/HRP-biocatalyst-modified CDN with the LDH/NAD^+^-biocatalyst-functionalized CDN, a composite CDN is assembled. Triggering the composite CDN with two different fuel strands results in orthogonal transient upregulation of the GOx/HRP cascade and transient downregulation of the LDH/NAD^+^ cascade or *vice versa*. The transient CDNs-guided biocatalytic cascades are computationally simulated by kinetic models, and the computational analyses allow the prediction of the performance of transient biocatalytic cascades under different auxiliary conditions. The concept of orthogonally triggered temporal, transient, biocatalytic cascades by means of CDN frameworks is applied to design an orthogonally operating CDN for the temporal upregulated or downregulated transient thrombin-induced coagulation of fibrinogen to fibrin.

## Introduction

Diverse intracellular dynamic interactions among DNA, RNA, proteins, and low-molecular-weight ligands form complex networks that are guided by intercommunicating stimuli and signal promotion. These networks activate key biological processes, such as cell division,^1−3^ signal transduction,^4^ cell motility,^5,6^ or gene expression and regulation.^7,8^ Inspired by nature, one of the challenges of systems chemistry is to emulate the operating principles of the native processes by artificial means and to harness the biomimetic systems for practical applications.^9−11^

The base sequence encoded in nucleic acids provides a rich “toolbox” of structural and functional information that can be used to construct biomimetic circuits and networks emulating native processes. Indeed, the catalytic functions of nucleic acids, such as DNAzymes,^12,13^ the sequence-specific recognition properties of aptamers,^14,15^ the dynamic reconfiguration of nucleic acid nanostructures,^16,17^ and the sequence-dictated reactivity patterns of nucleic acids in the presence of enzymes, e.g., polymerase,^18−20^ nickase,^21,22^ ligase,^23,24^ endonucleases,^25−27^ and exonucleases,^28^ were extensively used to develop dynamic reaction modules, networks, and circuits mimicking elements of native systems. For example, constitutional dynamic networks, CDNs, revealing reversible dynamic reconfiguration in the presence of auxiliary triggers, such as fuel strands,^29^ metal ions,^29^ or light,^30^ were demonstrated. In analogy to native networks, these bioinspired frameworks demonstrated adaptive,^31^ feedback-driven,^32^ and intercommunicating features.^33^ Moreover, dynamic temporally transient, reaction modules emulating native out-of-equilibrium dissipative transformations were engineered.^34−36^ In these systems, nucleic acid frameworks were subjected to external fuel agents^37^ or an energy source, e.g., light,^38^ to yield temporal intermediates that are temporally depleted in the presence of enzymes,^39,40^ DNAzyme,^41^ or heat,^38^ to generate “waste” products that recover the original “rest” reaction modules. Diverse applications of dynamically operating CDNs or transiently driven reaction modules were reported. These included the CDNs-dictated synthesis of hydrogel matrices revealing dynamic switchable stiffness properties and controlled drug release features^42^ and the CDNs-guided operation of enzyme cascades^43^ or dynamic photosynthetic processes.^44^ Also, transient reconfiguration of CDN frameworks was demonstrated.^45^ In addition, dissipative, transient, reaction circuits were applied for controlled formation of fibrils,^46^ transient biocatalytic reactions,^47^ transient aggregation/disaggregation of nanoparticles or semiconductor quantum dots leading to temporally programmed optical functionalities,^48^ and temporal synthesis of DNAzymes.^49^ Moreover, dynamic transcription machineries, including transcriptional oscillators,^50^ transcriptional switches,^51^ bistable genetic regulatory networks,^52^ transcriptional clocks,^53^ and topologically modulated temporal transcription machineries^54,55^ were demonstrated.

The previous work in the area of dynamic DNA networks has emphasized, however, the adaptivity, hierarchical adaptivity, network intercommunication and feedback-driven reconfiguration of dynamically equilibrated networks,^56^ and the feature of transient dissipative, out-of-equilibrium, networks operated by fuel-driven enzymes coupled to dissipative biocatalysts.^57−59^ The coupling of the different dynamic networks to biocatalytic cascades is, however, less established.^36^ In particular, the assembly of cooperatively operating equilibrated dynamic networks and transient dissipative networks, acting as functional frameworks that control biocatalytic cascades, is unprecedented. Moreover, besides enhanced functional complexities introduced by biocatalytic cascades driven by coupled equilibrated and dissipative dynamic frameworks, emerging applications driven by such systems may be envisaged. In the present study, we introduce the assembly of biocatalyst-functionalized equilibrated CDNs and transient, dissipative networks as functional frameworks driving cooperatively temporal biocatalytic cascades. The integrated systems reveal important emerging functions and introduce potential applications of the hybrid systems: (i) The spatial proximity between the biocatalysts tethered to the network constituents provides a confined nanoenvironment for the operation of biocatalytic cascades,^60^ a clear advantage over the operation of biocatalysts in a diffusional mixture in solution. (ii) The dynamic reconfiguration of the CDNs allows the programmed directional control over the contents of the constituents by triggering the stabilities of agonist/antagonist constituents, thereby guiding the directional efficacies of the temporal, transient biocatalytic cascades coupled to the constituents. (iii) By mixing two different intercommunicated biocatalytic networks, the orthogonal gated operation of the CDNs-guided transient dissipative circuits is demonstrated. (iv) By conjugation of a therapeutically significant protein (thrombin) into a hybrid equilibrated dynamic network and dissipative, transient network, the programmed temporal upregulation or downregulation of the trombin activities by a composite dynamic framework is demonstrated.

Here we report on the organization of DNA frameworks that couple CDNs to transient dissipative transformations. We demonstrate the fuel-guided thermodynamically controlled dynamic reconfiguration of CDNs that is perturbed by out-of-equilibrium degradation of the reconfigured networks, leading to transient reconfiguration of the CDNs. Furthermore, we tether biocatalysts to the constituents comprising the networks, and these drive the glucose oxidase (GOx)/horseradish peroxidase (HRP) biocatalytic cascade or the lactate dehydrogenase (LDH)/nicotinamide adenine dinucleotide (NAD^+^) biocatalytic cascade. By applying two different fuel triggers, directional transient operation of the GOx/HRP or of the LDH/NAD^+^ cascades is demonstrated. In addition, by mixing the GOx/HRP-modified and LDH/NAD^+^-tethered CDNs, a composite network driving the two transient biocatalytic cascades is assembled. By applying two different triggers on the composite networks, the orthogonal transient operation of upregulated transient GOx/HRP and downregulated transient LDH/NAD^+^ cascades or *vice versa* are demonstrated. The experimental results are computationally simulated with appropriate kinetic models. The computational analyses allow us to predict and experimentally validate the network-guided transient dynamic biocatalytic behaviors under different auxiliary conditions. Moreover, to demonstrate the possible practical utility of dynamic network-guided directional and orthogonal transient biocatalytic cascades, we introduce a dynamic network that controls the orthogonal upregulation/downregulation of the biocatalytic functions of thrombin. In these systems, the orthogonal, transient, upregulated and downregulated fibrinogen coagulation properties are demonstrated, thus revealing potential medical applications of the dynamic networks.

## Results and Discussion

**Figure 1:**
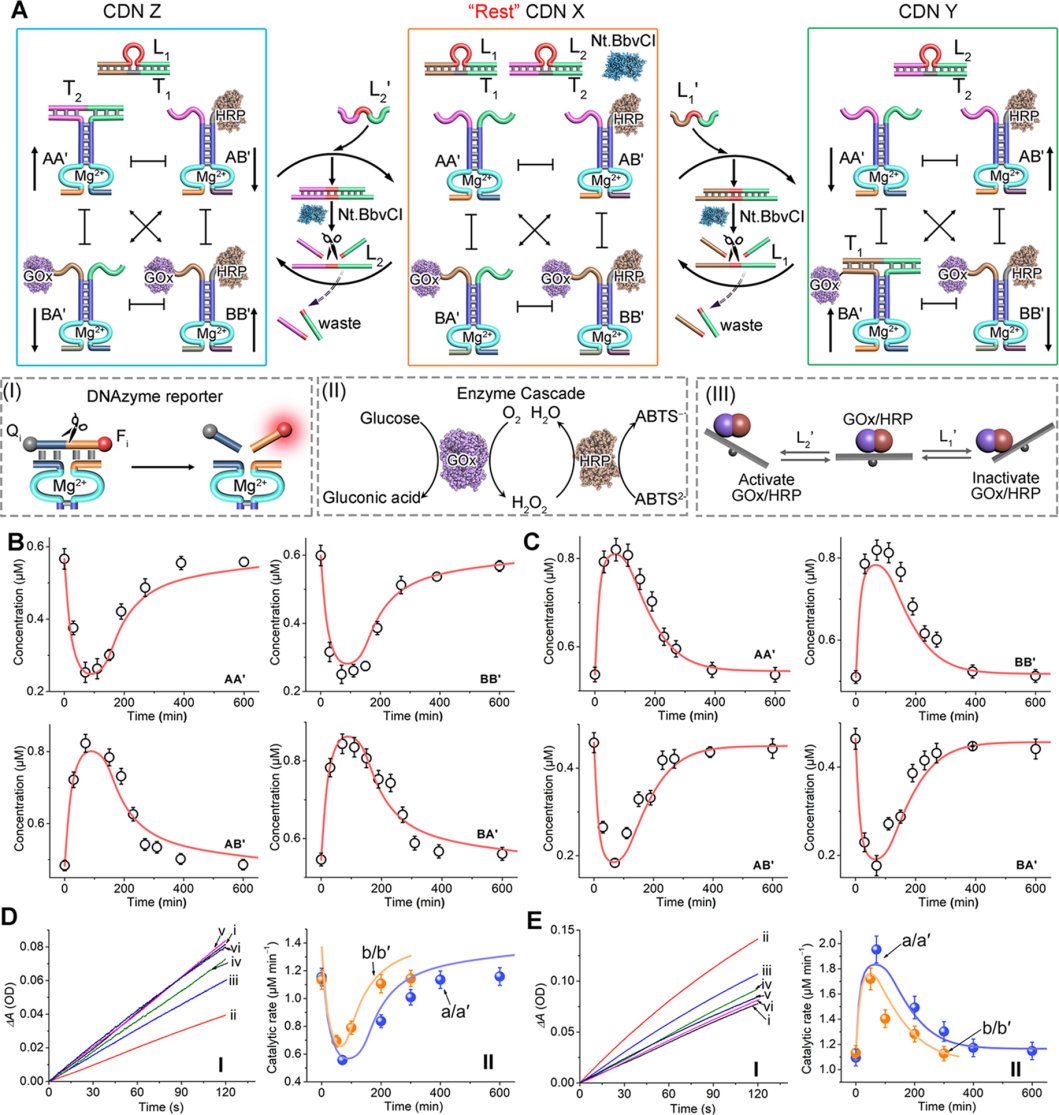
(A) Directional CDNs-guided transient GOx/HRP cascades. The concentrations of the constituents are probed by the DNAzyme reporter units associated with the constituents that cleave the respective fluorophore (F_i_)/quencher (Q_i_)-modified substrates, panel I. The biocatalytic cascade corresponds to the aerobic oxidation of glucose to form gluconic acid and H_2_O_2_ and the subsequent H_2_O_2_-induced oxidation of ABTS^2–^ to ABTS^•–^. The cascade is followed by colorimetric temporal evaluation of ABTS^•–^, λ = 420 nm, panel II. Panel III-Schematic directional operation of the transient GOx/HRP cascade. (B) Temporal concentration changes of the constituents upon the L_1_′-triggered reconfiguration of CDN X to CDN Y and its transient recovery to CDN X (curves consisting of dots, experimental data; solid curves, computationally simulated transient concentration changes using the kinetic model formulated in Figure S14). (C) Temporal concentration changes of the constituents upon the L_2_′-triggered reconfiguration of CDN X to CDN Z and its transient recovery to CDN X (curves consisting of dots, experimental data; solid curves, computationally simulated transient concentration changes using the kinetic model formulated in Figure S15). (D) panel I: Time-dependent concentration changes of ABTS^•–^ generated by transient reaction samples of the GOx/HRP cascade upon the L_1_′ (6 μM)-triggered dynamic reconfiguration of CDN X to CDN Y and back: after (i) *t* = 0, (ii) 100 min, (ii) 200 min, (iv) 300 min, (v) 400 min, and (vi) 600 min. Panel II: Transient catalytic rates of the GOx/HRP cascade upon reconfiguration of CDN X to CDN Y and back (curve a, dots, experimental data; solid curve a′, computational data at L_1_′ = 6 μM; curve b, dots, experimental data; solid curve b′, computational data at L_1_′ = 4 μM). (E) Panel I: Time-dependent concentration changes of ABTS^•–^ generated by transient reaction samples of the GOx/HRP cascade upon the L_2_′ (6 μM)-triggered dynamic reconfiguration of CDN X to CDN Z and back: after (i) *t* = 0, (ii) 100 min, (ii) 200 min, (iv) 300 min, (v) 400 min, (vi) 600 min. Panel II: Transient catalytic rates of the GOx/HRP cascade upon reconfiguration of CDN X to CDN Z and back (curve a, dots, experimental data; solid curve a′, computational data at L_2_′ = 6 μM; curve b, dots, experimental data; solid curve b′, computational data at L_2_′ = 4 μM). Experimental conditions: A, A′, B, B′, L_1_/T_1_, L_2_/T_2_ each 1 μM; nickase (Nt.BbvCI) 0.0345 μM; L_1_′, L_2_′ each 6 μM. For the predicted/experimentally validated curves b/b′ in (D) and (E), the concentrations of L_1_′/L_2_′ corresponded to 4 μM. Error bars are derived from *N* = 4 experiments.

Figure 1A schematically depicts the transient and adaptive CDN-guided bidirectional temporal operation of the GOx/HRP cascade. The “rest” [2 × 2] CDN X consists of four equilibrated constituents AA′, AB′, BA′, and BB′, where the nucleic acid component B in the different constituents is functionalized with GOx (molar ratio of GOx:B = 1:1), and the nucleic acid component B′ is modified with HRP (molar ratio of HRP:B′ = 1:1). For the purification and characterization of the precise 1:1 molar ratio of conjugates, including GOx-B and HRP-B′, LDH-D′, and C-NAD^+^, quantitative validation of these enzyme–DNA conjugates, and an assessment of the activities of the enzymes in the conjugates, see Figures S1–S7, Supporting Information, and accompanying discussion. Each of the constituents is integrated into a Mg^2+^-ion-dependent DNAzyme unit that binds to the respective fluorophore/quencher (F_i_/Q_i_)-modified substrate, and these act as catalytic reporter units for probing the dynamic contents of the respective constituents in the system(s), Figure 1A, panel I. By following the cleavage rate of the fluorophore/quencher-modified substrates by the respective Mg^2+^-ion-dependent DNAzymes, and using appropriate calibration curves, Figures S8 and S9, the quantitative evaluation of the contents of the constituents is possible, *vide infra*. Also, each of the constituents (AA′ and BA′) includes two different tethers acting as binding “arms” for appropriate programmed auxiliary fuel strands, allowing the control over the stability of a target constituent and the adaptive dynamic reconfiguration of the CDN. In addition, the duplexes L_1_/T_1_ and L_2_/T_2_, and the nicking enzyme Nt.BbvCI, are added as co-agents into the “rest” CDN X module, and these constituents operate the transient biocatalytic cascade of the CDN. Subjecting CDN X to the fuel-strand L_1_′ results in the displacement of duplex L_1_/T_1_ to yield the duplex structure L_1_/L_1_′ and the release of T_1_. The released strand T_1_ is engineered, however, to bind to the arms of constituent BA′, and stabilization of BA′-T results in its upregulation and the concomitant upregulation of AB′, while the antagonist constituents AA′ and BB′ are adaptively downregulated. As a result, the downregulation of the content of BB′ decreases the efficacy of the GOx/HRP cascade, Figure 1A, panel II, as compared to the GOx/HRP catalytic cascade driven by the parent CDN X. The L_1_′/L_1_ duplex is engineered, however, to include a nicking site in L_1_′ for Nt.BbvCI, which generates fragments of L_1_′ as waste and releases L_1_ as a functional strand to separate BA′-T_1_, leading to the temporal recovery of the “rest” CDN X. (For the purification of the nickase used in the system, see the experimental section in the Supporting Information and Figure S10.) That is, subjecting CDN X to the fuel strand L_1_′ leads to the temporal reconfiguration of CDN X to CDN Y and to the transient depletion of CDN Y and recovery of the parent CDN X. The dynamic adaptive reconfiguration of CDN X to CDN Y and back dynamically guides the control over the catalytic GOx/HRP cascade, where the reconfiguration of CDN X to CDN Y is accompanied by a dynamic primary decrease of the efficacy of the biocatalytic cascade, followed by transient recovery of the initial efficiency of the GOx/HRP cascade. Similarly, subjecting CDN X to the trigger L_2_′ results in the separation of the duplex L_2_/T_2_ to form the duplex L_2_′/L_2_ and the separated strand T_2_ that binds to the constituent AA′. Stabilization of AA′-T_2_ leads to the dynamic adaptive reconfiguration of CDN X to CDN Z, where constituents AA′-T_2_ and BB′ are upregulated and the antagonist constituents BA′ and AB′ are downregulated. That is, the dynamic adaptive reconfiguration of CDN X to CDN Z and upregulation of AA′-T_2_ promote the upregulation of the agonist constituents composed of GOx/HRP-BB′. This results in the dynamic enhancement of the GOx/HRP cascade as compared to the activity of the GOx/HRP cascade in the parent CDN X. The strand L_2_′ is engineered, however, to be cleaved by Nt.BbvCI, resulting in the concomitant release of L_2_ to the form duplex L_2_/T_2_ and the temporal recovery of CDN X, thus depleting the efficiency of the enhanced GOx/HRP cascade. That is, the L_2_′-triggered dynamic reconfiguration of CDN X → Z → X leads to temporal positive enhancement of the GOx/HRP cascade, followed by the transient depletion of the enhanced catalytic cascade to the original catalytic functions embedded in the “rest” module of CDN X. Thus, the triggered dynamic reconfiguration of CDN X with the input L_1_′ or L_2_′ leads to the transient formation of CDN Y or CDN Z that guides the bidirectional transient biocatalytic cascades, reflected by transient negative efficacy in the presence of L_1_′ and transient positive efficacy in the presence of L_2_′.

In the first step, the contents (concentrations) of the constituents associated with the dynamically and temporally reconfigured CDNs were confirmed. Toward this goal, the time-dependent fluorescence generated by cleavage of the F_i_/Q_i_-modified substrates by the DNAzyme units associated with four constituents was monitored on samples extracted from the temporal reaction modules driving the transient transition of CDN X → Y → X (in the presence of L_1_′) or CDN X → Z → X (in the presence of L_2_′), Figures S11 and S12. Treatment of CDN X with L_1_′ reveals an initial enhanced cleavage rate of the DNAzyme units associated with constituents BA′-T_1_ and AB′ (implying upregulation of these constituents) and initial retardation of the substrate cleavage rates of the DNAzyme units associated with constituents AA′ and BB′ (indicating downregulation of the AA′ and BB′), Figure S11. The upregulated activities of the DNAzymes associated with BA′-T_1_ and AB′ and the downregulated activities of the DNAzymes associated with AA′ and BB′ reveal temporal behavior and are recovered within ca. 8 h to the DNAzyme activities characterizing the “rest” module, CDN X. Similarly, Figure S12 depicts the time-dependent cleavage rates of the substrates by the respective DNAzymes upon subjecting CDN X to the trigger L_2_′. The initial cleavage rates of the DNAzymes associated with AA′-T_2_ and BB′ are upregulated as compared to the “rest” module and then temporally decrease to the parent DNAzyme activities in the “rest” CDN X. Also, the rates of the DNAzymes associated with BA′ and AB′ concomitantly decrease as compared to the cleavage rates of the DNAzymes in the “rest” CDN X. The upregulated and downregulated cleavage rates are recovered within ca. 8 h. Using appropriate calibration curves relating the cleavage rates of the respective substrates as a function of the different DNAzyme concentrations, the concentrations of each of the constituents upon the temporal operation of the CDNs were evaluated. It should be noted that the cleavage rates of the substrates by the DNAzyme reporter units on the samples withdrawn from the dynamically operating modules are probed on a time scale of 30 min, as compared to the substantially longer time scale (8 h) of the temporal reaction modules, and, thus, the concentration changes of the constituents within the probing time intervals can be neglected. (For probing the dynamics of the reconfigured CDNs at shorter time intervals of 10 min, see Figure S13 and accompanying discussion.) Figure 1B (curves consisting of dots) shows the temporal transient concentrations of the constituents upon the L_1_′-triggered transition of CDN X to CDN Y and the transient recovery of CDN X. The concentrations BA′ and AB′ are temporally upregulated by ca. 155% and reach maximum values at ca. 70 min, afterward gradually decaying to lower concentrations and recovering the original concentrations after ca. 600 min. In parallel, the constituents AA′ and BB′ reveal “mirror image” negative transient concentration profiles. The concentrations of these constituents decrease within the first 70 min of transition of CDN X to CDN Y, and afterward a transient gradual time-dependent increase in the concentrations of these constituents is observed and the original concentrations of the constituents in CDN X are recovered after ca. 600 min. For further support of the temporal concentration changes of the constituents upon the L_1_′-triggered transition of CDN X to CDN Y and back by quantitative gel electrophoretic experiments, see Figure S16, Supporting Information, and accompanying discussion. Similarly, the transient temporal concentrations of the constituents upon the L_2_′-triggered transient transition of CDN X → Z → X, are displayed in Figure 1C, curves consisting of dots. In this case, stabilization of constituent AA′-T_2_ leads to the temporal positive upregulation of the concentrations of AA′ and BB′, reaching maximum intermediate concentration at ca. 70 min, followed by the gradual temporal decay in the concentrations of these constituents, to the parent concentration values in CDN X after ca. 600 min. Thus, subjecting CDN X to trigger L_1_′ or L_2_′ yields bidirectional transient concentration profiles for the constituents upon dynamic reconfiguration of CDN X → Y → X or CDN X → Z → X. While the L_1_′-stimulated transition of CDN X to CDN Y is accompanied by the “negative” control of the concentrations of AA′ and BB′, subjecting CDN X to L_2_′ results in the “positive” transient upregulation of the concentrations of constituents AA′ and BB′. It should be noted that mutation of the loop domains of L_1_/T_1_ and L_2_/T_2_ in a manner noncomplementary to L_1_′/L_2_′ did not allow the L_1_′- or L_2_′-triggered dynamic reconfiguration of CDN X to CDN Y or CDN Z.

The GOx/HPR cascade in the transient reconfigured CDN Y and CDN Z networks is controlled and dictated by the concentration of biocatalysts conjugated to the BB′ constituent. While the downregulation of the concentration of GOx/HRP-BB′ upon L_1_′-triggered transition of CDN X to CDN Y is anticipated to induce temporal, transient, downregulation of the biocatalytic cascade, the temporal concentration enrichment of the GOx/HRP-BB′ constituent upon L_2_′-triggered transition of CDN X to CDN Z is expected to stimulate transient positive upregulation of the biocatalytic cascade. Figure 1D and E depict the efficacies of the transient GOx/HRP biocatalyst cascades upon the L_1_′/L_2_′-driven dynamic transition of CDN X to CDN Y or to CDN Z, respectively. The efficiency of the transient biocatalytic cascade was followed by probing at time intervals the generation of the colored 2,2′-azino-bis(3-ethylbenzothiazoline-6-sulfonic acid) radical anion (ABTS^•–^) in samples extracted from the transiently operating modules, Figure 1D, panel I. From the kinetic curves at time intervals of biocatalytic cascaded operation, the temporal catalytic rates corresponding to the biocatalytic cascades were evaluated, Figure 1D, panel II, curve (a), dots, representing the experimental results at different time intervals. The L_1_′-fueled dynamic transition of CDN X to CDN Y leads to the transient downregulation of the GOx/HRP cascade, as compared to the activity of the GOx/HRP cascade in CDN X, while the L_2_′-fueled reconfiguration of CDN X to CDN Z leads to the upregulated transient operation of the GOx/HRP cascade, Figure 1E panel I, and curve (a), dots, in panel II. The experimental transient dynamic concentration changes of the constituents upon the L_1_′- or L_2_′-triggered reconfiguration of CDN X → Y → X or CDN X → Z → X (Figure 1B and C, curves consisting of dots) were computationally simulated. Kinetic models following the dynamic reconfigurations of CDN X → Y → X and CDN X → Z → X were formulated, Figure S14 and Figure S15. The rate constants leading to the best fit of the transient reconfiguration curves (solid curves overlaid on the experimental dots) were evaluated, and these are summarized in Tables S2 and S3. For the transient concentrations of the constituent BB′ bearing the biocatalyst GOx/HRP components and the calibration curve relating the rates of the catalytic cascade to variable concentrations of the biocatalyst constituent, see Figure S17. The computationally calculated transient catalytic rates of the GOx/HRP cascade were evaluated, and these are displayed in the solid curve (a′) overlaid on the experimental transient catalytic rates, curve (a), dots characterizing the dynamic transition of CDN X → Y → X, Figure 1D, panel II, curves a/a′, and the dynamic transition of CDN X → Z → X, Figure 1E, panel II, curves a/a′. The kinetic computational simulation of the experimental results and the derived rate constants have a value only if the computational data can predict the behavior of the transient biocatalytic cascade under different auxiliary conditions that can be evaluated later by experiments. Accordingly, while the original transient transitions of CDN X → Y → X and CDN X → Z → X were triggered with fuel concentrations of L_1_′/L_2_′ = 6 μM, the fuel concentrations were altered to L_1_′/L_2_′ = 4 μM to validate the transiently predicted catalytic rates. Using the set of rate constants provided in Tables S2 and S3, the transient concentrations of the biocatalytic constituent were computationally evaluated and translated into computationally predicted transient catalytic rates of the GOx/HRP cascade in CDN X → Y → X, Figure 1D, panel II, solid curve (b′), and the GOx/HRP cascade in CDN X → Z → X, Figure 1E, panel II, solid curve (b′). (For the time-dependent absorption curves of GOx/HRP cascade-driven generation of ABTS^•–^ in the transient L_1_′/L_2_′ (4 μM)-triggered transition of CDN X → Y → X or CDN X → Z → X, see Figure S18.) The computationally predicted dynamic curves were then experimentally validated at the altered L_1_′/L_2_′ concentrations (4 μM), curve b, dots, Figure 1D and E, panel II. A very good fit between the computational and experimental results is observed, supporting the validity of the kinetic models. That is, the results demonstrate that conjugation of biocatalytic cascades to the dynamically fuel-driven reconfigurable CDNs coupled to the transient, dissipative, reaction modules has the capacity to control the directional and dose-controlled downregulation or upregulation of the biocatalytic cascade, thus acting as complex systems that emulate the native dynamic control of biocatalytic cascaded transformations. Two negative control experiments to support the dynamic operation of the “rest” CDN X were performed: (i) In one experiment, the base sequence of the conjugate HRP-B′ was randomized to HRP-B″, eliminating the possibility to yield the constituent GOx-B/HRP-B′′. Under these conditions, the entire CDN X was not formed, and the concentration of AA′ remained constant (1 μM). Furthermore, the biocatalytic GOx-B and HRP-B′ in the mixture were extremely inefficient, similar to the activity of the separated diffusional component GOx-B and HRP-B′. (ii) In a second control experiment, the L_1_ strand in the duplex L_1_/T_1_ was exchanged with a mutated strand L_1_^M^ that could not be displaced by the fuel strand L_1_′. Subjecting the CDN X reaction module to the trigger L_1_′ did not affect the dynamic reconfiguration of CDN X to CDN Y, and the biocatalytic cascade associated with the constituent GOx-B/HRP-B′ remained constant before and after triggering the system with L_1_′, Figure S19. These results indicate that the L_1_′-triggered activation of CDN X leads, indeed, to the dynamic reconfiguration of CDN X to CDN Y and the accompanying transient recovery of CDN X.

**Figure 2:**
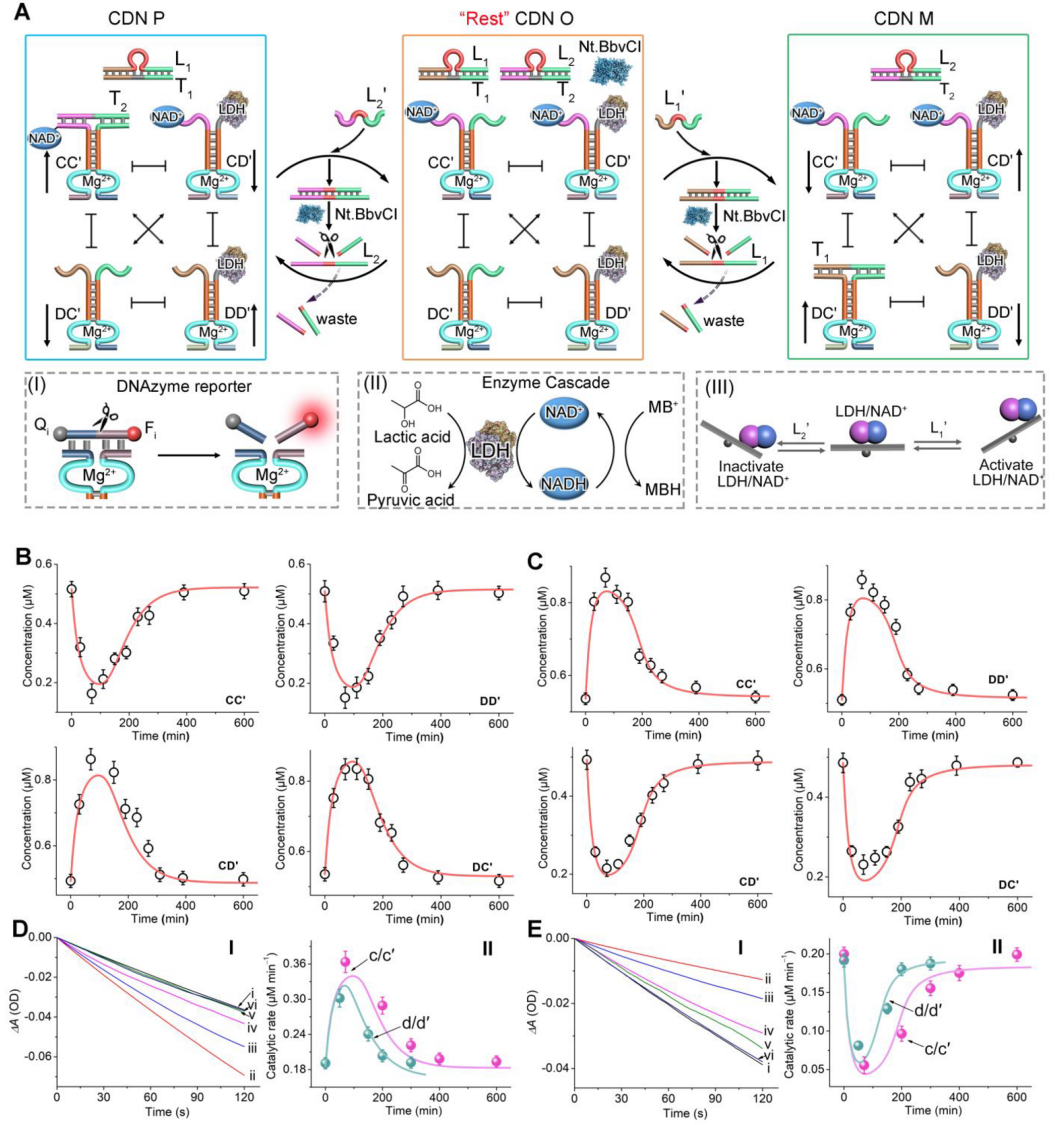
(A) Directional CDNs-guided transient LDH/NAD^+^ cascades. The concentrations of the constituents are probed by the DNAzyme reporter units associated with the constituents that cleave the respective fluorophore (F_i_)/quencher (Q_i_)-modified substrates, panel I. The biocatalytic cascade corresponds to the LDH-catalyzed reduction of NAD^+^ by lactic acid, and the temporal formation of NADH is probed by the interaction of NADH with MB^+^ by following the absorbance changes of MB^+^ (λ = 664 nm), panel II. Panel III-Schematic directional operation of the transient LDH/NAD^+^ cascade. (B) Temporal concentration changes of the constituents upon the L_1_′-triggered reconfiguration of CDN from O to CDN M and its transient recovery to CDN O (dots, experimental data; solid curves, computationally simulated transient concentration changes using the kinetic model formulated in Figure S24). (C) Temporal concentration changes of the constituents upon the L_2_′-triggered reconfiguration of CDN O to CDN P and its transient recovery to CDN O (dots, experimental data; solid curves, computationally simulated transient concentration changes using the kinetic model formulated in Figure S25). (D) Panel I: Time-dependent concentration changes of MB^+^ depleted by transient reaction samples of the LDH/NAD^+^ cascade upon the dynamic reconfiguration of CDN X to CDN Y and back: after (i) *t* = 0, (ii) 100 min, (ii) 200 min, (iv) 300 min, (v) 400 min, and (vi) 600 min. Panel II: Transient catalytic rates of the LDH/NAD^+^ cascade upon reconfiguration of CDN O to CDN M and back (curve c, dots, experimental data; solid curve c′, computational data at L_1_′ = 6 μM; curve d, dots, experimental data; solid curve d′, computational data at L_1_′ = 4 μM). (E) Panel I: Time-dependent concentration changes of MB^+^ depleted by transient reaction samples of the LDH/NAD^+^ cascade upon the dynamic reconfiguration of CDN O to CDN P and back: after (i) *t* = 0, (ii) 100 min, (ii) 200 min, (iv) 300 min, (v) 400 min, (vi) 600 min. Panel II: Transient catalytic rates of the LDH/NAD^+^ cascade upon reconfiguration of CDN O to CDN P and back (curve c, dots, experimental data; solid curve c′, computational data at L_1_′ = 6 μM; curve d, dots, experimental data; solid curve d′, computational data at L_1_′ = 4 μM). Experimental conditions: C, C′, D, D′, L_1_/T_1_, L_2_/T_2_ each 1 μM; nickase (Nt.BbvCI) 0.0345 μM; L_1_′, L_2_′ each 6 μM. For the predicted/experimentally validated curves d/d′ in (D) and (E), the concentration of L_1_′/L_2_′ corresponded to 4 μM. Error bars derived are from *N* = 4 experiments.

The concept of applying the dynamic reconfiguration of a constitutional dynamic network as a functional framework that controls the transient operation of a biocatalytic cascade was further extended to include a different biocatalytic cascade consisting of the LDH-catalyzed oxidation of lactic acid to pyruvic acid and the concomitant reduction of oxidized nicotinamide adenine dinucleotide (NAD^+^) to reduced nicotinamide adenine dinucleotide (NADH). Specifically, we discuss the application of the fuel strands L_1_′ or L_2_′ that were applied for the reconfiguration of the CDNs guiding the transient operation of the GOx/HRP cascade, cf. Figure 1, as triggers to guide the reconfiguration of the CDNs guiding the transient operation of the LDH/NAD^+^ cascade. We note, however, that the structural engineering of the CDN constituents driving the LDH/NAD^+^ cascade will follow the principle that the L_1_′- or L_2_′-triggered reconfiguration of the CDN constituents are designed to stabilize/destabilize the constituents in orthogonal directions operating in CDN driving the GOx/HRP cascade. This not only allows the L_1_′/L_2_′-triggered reconfiguration of the CDN assemblies guiding the transient operation of the directional LDH/NAD^+^ cascades but will enable the mixing of the GOx/HRP-CDNs and the LDH/NAD^+^-CDNs and the operation of the composite CDN assembly as an orthogonally gated biocatalytic cascaded framework guided by the trigger L_1_′ or L_2_′. The mixture of CDNs then offers a model system for triggering transient orthogonal biotransformations by dynamic networks. Figure 2A depicts the composition and operation of the L_1_′- or L_2_′-triggered reconfiguration of CDN O to yield the transient reconfigured CDN M or CDN P and the transient directional operation of the LDH/NAD^+^ cascade (in orthogonal directions to the L_1_′/L_2_′-guided operation of the GOx/HRP cascade). The “rest” CDN O is composed of four constituents CC′, CD′, DC′, and DD′, where the component D′ is tethered to the LDH biocatalyst and component C is linked to the NAD^+^ cofactor. (For the purification and characterization of the precise 1:1 molar ratio of LDH-D′ and NAD^+^-C, see Figures S5–S7, Supporting Information, and accompanying discussion.) The duplexes L_1_/T_1_ and L_2_/T_2_ and the nicking enzyme Nt.BbvCI are added as functional agents to CDN O. Each of the constituents comprising CDN O is functionalized with the DNAzyme reporting units, allowing the quantitative assessment of the concentrations of the constituents in CDN O or the transient concentrations of the constituents in the L_1_′-reconfigured CDN M or the L_2_′-reconfigured CDN P. Subjecting CDN O to trigger L_1_′ results in the displacement of the duplex L_1_/T_1_ to yield the duplex L_1_/L_1_′ and the free strand T_1_. The strand T_1_ binds and stabilizes constituent DC′, resulting in the reconfiguration of CDN O to CDN M, where the DC′-T_1_ constituent is upregulated, the antagonist constituents CC′ and DD′ are downregulated, and the agonist constituent CD′ is concomitantly upregulated. The strand L_1_′ in the duplex L_1_/L_1_′ is engineered to include a nicking site for Nt.BbvCI, resulting in the cleavage of L_1_′ to two fragmented “waste” products that release strand L_1_. The released strand L_1_ displaces, however, the bridging strand T_1_, resulting in the recovery of CDN O.

That is, treatment of CDN O with L_1_′ leads to the transient reconfiguration of CDN O to CDN M and the dynamic depletion of CDN M to CDN O. The transient concentration changes of the constituents upon the dynamic reconfiguration of CDN O to CDN M were evaluated by following the cleavage rates of the fluorophore/quencher (F_i_/Q_i_)-modified substrates by the reporters conjugated to the constituents, Figure 2A, panel I, and using appropriate curves, Figures S20 and S21. The transient concentration changes of the constituents upon the L_1_′-triggered dynamic reconfiguration of CDN O → M → O are displayed in Figure 2B, curves consisting of dots. Similarly, subjecting CDN O to the trigger L_2_′ results in the displacement of the duplex L_2_/T_2_, the formation of L_2_/L_2_′, and the release of free T_2_, which stabilizes constituent CC′ (CC′-T_2_). Stabilization of constituent CC′ by T_2_ guides the reconfiguration of CDN O to CDN P, where constituents CC′ and DD′ are upregulated, and concomitantly constituents CD′ and DC′ are downregulated. As the strand L_2_′ in the duplex L_2_/L_2_′ is engineered to be nicked by Nt.BbvCI, its cleavage by the nickase leads to the release of L_2_, which displaces T_2_ from the constituent CC′-T_2_, resulting in the recovery of CDN P to CDN O and to the transient transition of CDN O → P → O. The transient concentrations of the constituents accompanying the dynamic transition of CDN O → P → O are transduced by the DNAzyme reporter units associated with the constituents. The transient concentration changes of the constituents upon the dynamic transition CDN O → P → O are displayed in Figure 2C, curves consisting of dots. (For the temporal time-dependent cleavage rates of the substrates corresponding to the different DNAzyme reporter units, which allowed the evaluation of the transient concentration changes of the constituents, see Figures S22 and S23.) The temporal upregulation of constituent DC′-T_1_, upon reconfiguration of CDN O to CDN M, is accompanied by the upregulation of constituent CD′ that includes the bicatalyts LDH and NAD^+^ units that drive the LDH/NAD^+^ cascade. As a result, the dynamic reconfiguration of CDN O to CDN M is reflected by the temporal transient enhancement of the LDH-biocatalyzed oxidation of lactic acid and the concomitant reduction of NADH. The temporal catalytic rate changes of the biocatalytic cascade (LDH/NAD^+^) are then analyzed spectroscopically by following the kinetics of reduction of methylene blue (MB^+^) by NADH, Figure 2A, panel II. Figure 2D, panel I, depicts the time-dependent absorption changes of MB^+^, upon MB^+^ reduction by LDH/NAD^+^ samples withdrawn from the L_1_′-triggered CDN O at time intervals of the transient operation of CDN O → M → O. The temporal rates of MB^+^ reduction were, then, translated into the transient catalytic rate of the LDH/NAD^+^ cascade, Figure 2D, panel II, curve (c), dots. Similarly, Figure 2E, panel I, shows the time-dependent absorbance changes of MB^+^ upon the L_2_′-triggered MB^+^-reduction by LDH/NAD^+^ samples withdrawn from the CDN O at time intervals of the transient operation of CDN O → P → O. These time-dependent curves were translated into transient catalytic rates of the L_2_′-guided LDH/NAD^+^ cascade, Figure 2E, panel II, curve (c), dots. The results demonstrate directional control of the LDH/NAD^+^ upon the L_1_′/L_2_′-triggered reconfiguration of CDN O to CDN M or CDN P. While the L_1_′-triggered transition of CDN O → M → O leads to the transient upregulation of the LDH/NAD^+^ cascade, the L_2_′-triggered dynamic reconfiguration of CDN O → P → O leads to the transient downregulation of the LDH/NAD^+^ cascade. As before, the kinetics of the bidirectional L_1_′- or L_2_′-triggered transient transitions of CDN O → M → O and CDN O → P → O were computationally simulated. The respective subreactions comprising the kinetic models corresponding to these transitions are summarized in Figures S24 and S25. The best fit of the computational transient curves (solid curves) corresponding to the transient concentrations of the constituents associated with the transient reconfiguration of CDN O → M → O and CDN O → P → O is overlaid on the transient experimental concentrations of the constituents (curves, consisting of dots), Figure 2B and C, respectively. The set of rate constants derived from the best fitted curves is summarized in Tables S4 and S5. Using the calibration curve relating the rates of the LDH/NAD^+^ biocatalytic cascade to the concentrations of constituent CD′ bearing the biocatalyst components, Figure S26, the transient simulated catalytic rates of the LDH/NAD^+^ cascade (curve c′) were calculated and overlaid on the experimental LDH/NAD^+^ catalytic rates (curve c) corresponding to the transitions of CDN O → M → O (curves c/c′), Figure 2D, panel II, and to the transitions CDN O → P → O (curves c/c′), Figure 2E, panel II. (Note that the experimental and computational results associated with curves c/c′, Figure 2D and E, panel II, were derived at L_1_′/L_2_′ concentrations corresponding to 6 μM.) The computational results were, then, supported by the initial prediction of the catalytic rate of the LDH/NAD^+^ cascade of the transition of CDN O → M → O or CDN O → P → O at different auxiliary fuel concentrations, L_1_′ or L_2_′ (4 μM), curve d′ in panel II (Figure 2D and E), respectively, followed by experimental validation of the predicted catalytic curve d, dots, in Figure 2D and E, panel II. For the time-dependent absorbance changes of MB^+^ by LDH/NAD^+^ in the L_1_′/L_2_′ (4 μM)-triggered transient transitions of CDN O → M → O or CDN O → P → O, see Figure S27. A very good fit between the predicted catalytic rates and experimentally validated data is observed.

Note, however, that the L_1_′-triggered reconfigurations of CDN X to CDN Y and of CDN O to CDN M lead to opposite effects on the transient biocatalytic cascades associated with the respective CDNs, and while the GOx/HRP associated with the BB′ constituent of CDN Y is downregulated, the transient LDH/NAD^+^ biocatalytic cascade associated with constituent CD′ of CDN M is upregulated. Similarly, the L_2_′-triggered reconfiguration of CDN X to CDN Z leads to the upregulation of the GOx/HRP cascade through stabilization of constituent BB′, while the reconfiguration of CDN O to CDN P leads to the downregulation of the LDH/NAD^+^ cascade due to the downregulation of constituent CD′.

**Figure 3:**
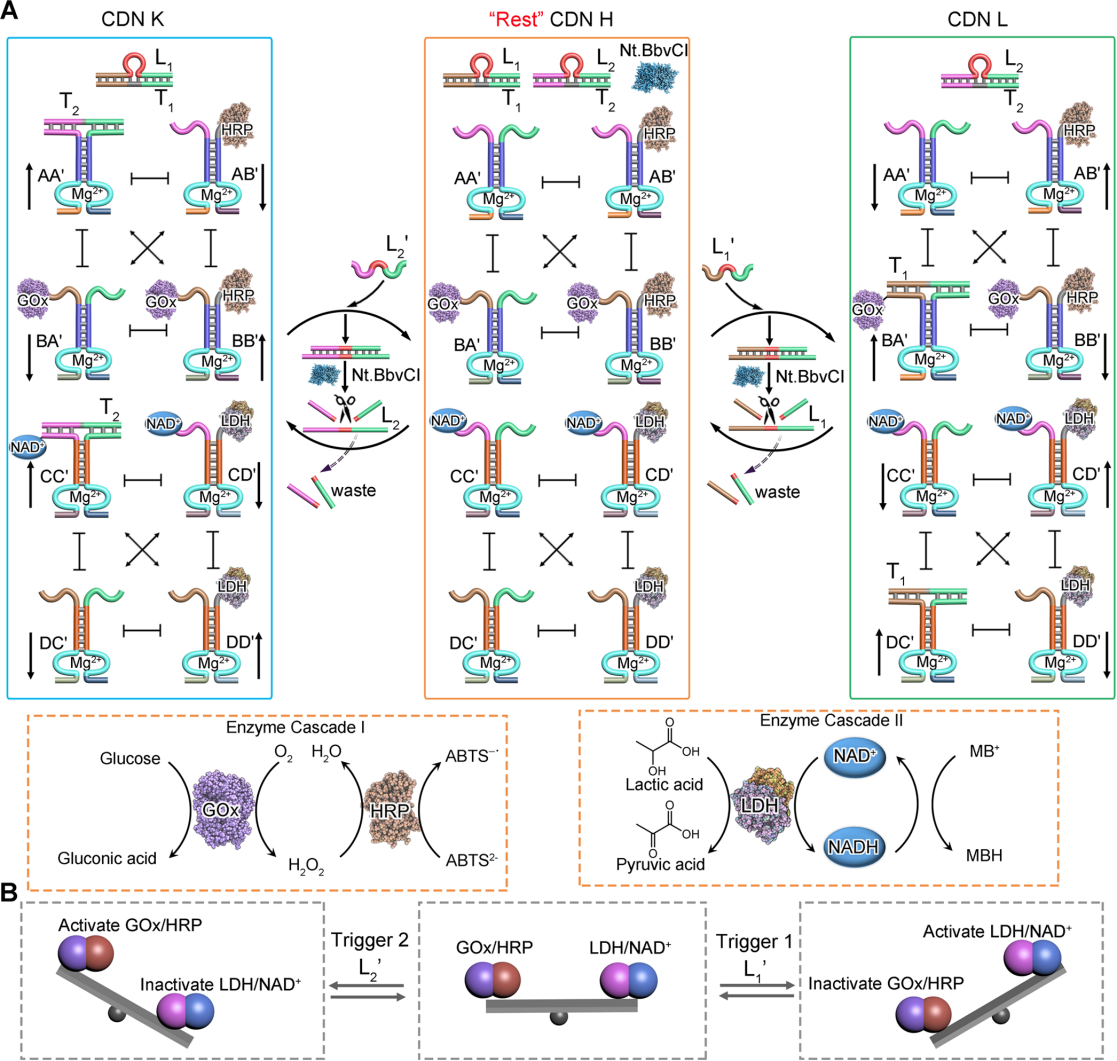
(A) Schematic orthogonal transient dynamic reconfiguration of two biocatalytic cascades composed of the L_1_′- or L_2_′-triggered dynamic reconfiguration of CDN H to CDN L and back or of CDN H to CDN K and back. (B) Schematic orthogonal upregulation/downregulation of the GOx/HRP and LDH/NAD^+^ biocatalytic cascades upon the fueled transient dynamic reconfiguration of CDN H: CDN H → L → H transiently upregulates the LDH/NAD^+^ cascade and transiently downregulates the GOx/HRP cascade. CDN H → K → H transiently upregulates the GOx/HRP cascade and transiently downregulates the LDH/NAD^+^ cascade.

Thus, the assembly of a reaction module that integrates the constituents included in CDN X and CDN O is anticipated to yield a composite CDN consisting of eight constituents, “rest” CDN H, that can be triggered by either L_1_′ or L_2_′. The integration of the two networks allows the L_1_′- or L_2_′-stimulated reconfiguration of “rest” CDN H into CDN L or CDN K, Figure 3A, where the two biocatalytic GOx/HRP and LDH/NAD^+^ cascades operate concomitantly, yet the fuel-triggered rate perturbations operate in opposite directions. While in CDN L the LDH/NAD^+^ cascade is upregulated and the GOx/HRP cascade is downregulated, in CDN K the GOx/HRP cascade is upregulated and the LDH/NAD^+^ cascade is downregulated. Beyond enhancing the complexity of integrated dynamic networks guiding dynamic directionally dictated transient biocatalytic processes, the system introduces catalytic orthogonality demonstrated by the intercommunicating networks. This principle is exemplified in Figure 3A, with the integration of CDN X and CDN O into a common reaction module, CDN H, that includes the duplexes L_1_/T_1_ and L_2_/T_2_ and the nicking enzyme Nt.BbvCI as auxiliary functional agents. Subjecting CDN H to trigger L_1_′ results in the displacement of duplex L_1_/T_1_ to yield L_1_/L_1_′ and the release of T_1_. The released strand T_1_ binds and stabilizes the constituents BA′ and DC′, resulting in the reconfiguration of CDN H to CDN L where constituents AA′, BB′ are downregulated and BA′, AB′ are upregulated and constituents CC′, DD′ are downregulated and DC′, CD′ are upregulated. Importantly, we note, however, that in CDN L the constituent BB′ carrying the GOx/HRP biocatalysts is downregulated, whereas the constituent CD′ carrying the LDH/NAD^+^ catalyst/cofactor pair is upregulated. The duplex L_1_/L_1_′ formed upon the reconfiguration of CDN H to CDN L is nicked by Nt.BbvCI to fragment L_1_′ and releases L_1_. The released L_1_ displaces T_1_ from the structures T_1_-BA′ and T_1_-DC′, leading to the temporal, transient recovery of CDN L to CDN H. The dynamic transient behavior of the constituents dictates then the orthogonal transient features of the biocatalytic transformations. The transient GOx/HRP biocatalytic cascade reveals a temporal decrease in efficacy, followed by the temporal recovery of the parent biocatalytic activity of CDN H, while the transient LDH/NAD^+^ biocatalyst cascade shows a temporal upregulation, followed by temporal decay to the parent LDH/NAD^+^ activities characterizing CDN H. Similarly, subjecting CDN H to L_2_′ results in the displacement of L_2_/T_2_ to yield the duplex L_2_/L_2_′ and strand T_2_ that stabilizes the constituents AA′ and CC′ and the reconfiguration of CDN H to CDN K, where the constituent BB′ bearing the GOx/HRP biocatalyst is upregulated, and the constituent CD′ carrying the LDH/NAD^+^ components is downregulated. The concomitant cleavage of strand L_2_′ in the intermediate duplex L_2_/L_2_′ by the nicking enzyme releases L_2_, which displaces T_2_ from the respective constituents to recover L_2_/T_2_. This leads to the transient transition of CDN H to CDN K, where the GOx/HRP cascade is upregulated and the LDH/NAD^+^ cascade is downregulated, followed by the dynamic recovery of CDN K to CDN H, where the parent activities of GOx/HRP and LDH/NAD^+^ are recovered. Thus, the L_2_′-guided dynamic transient transition of CDN H → K → H leads to orthogonal transient behaviors of the GOx/HRP and LDH/NAD^+^ cascades. The directional and orthogonal catalytic features of the L_1_′/L_2_′-triggered biocatalysts’ dynamic transient cascades are schematically presented in Figure 3B.

**Figure 4:**
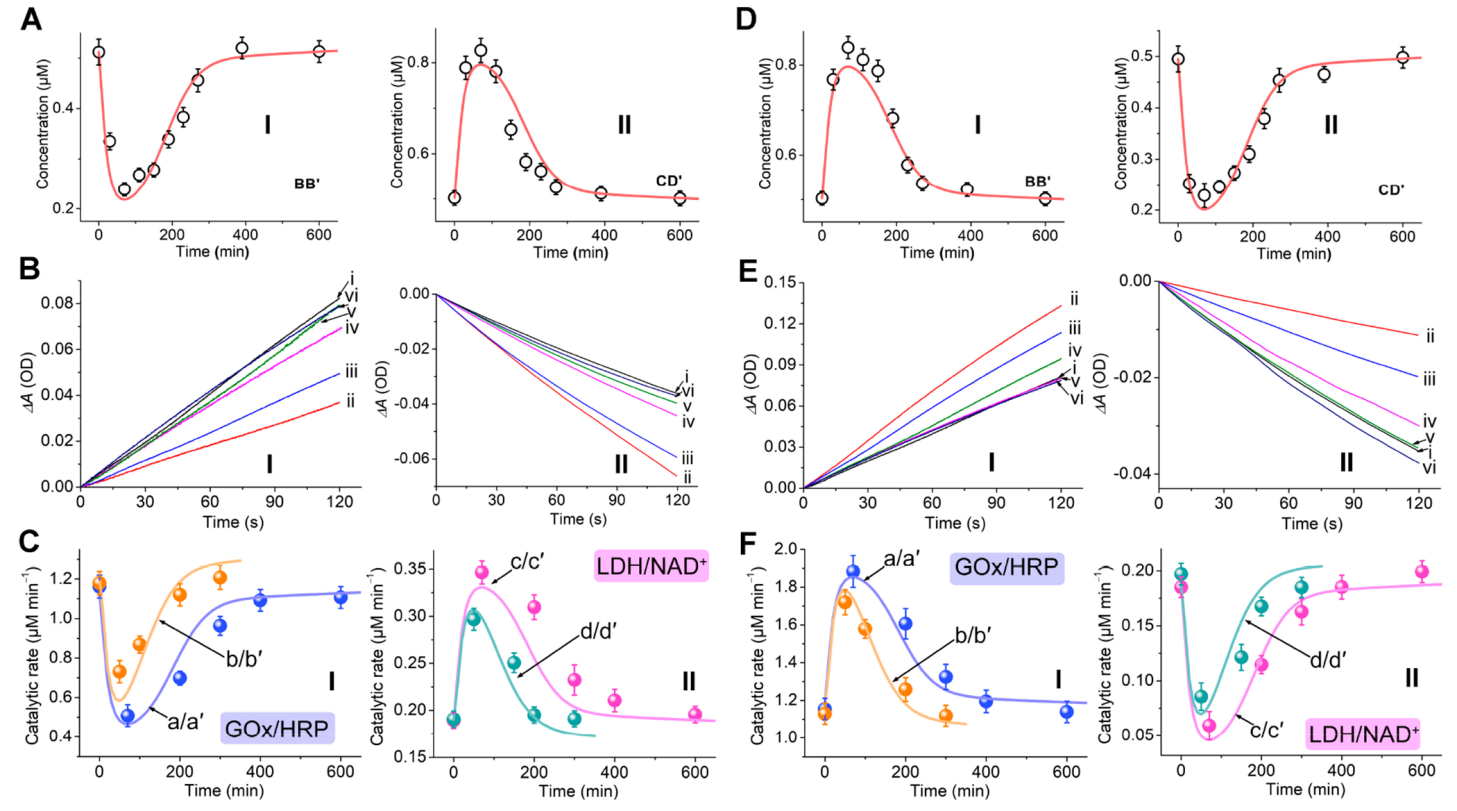
(A) Transient concentrations of constituents: panel I (BB′) and panel II (CD′) were subjected to the L_1_′-triggered reconfiguration of CDN H to CDN L and the recovery of CDN H. (B) L_1_′ (12 μM)-triggered time-dependent absorbance changes generated by panel I (the GOx/HRP cascade oxidizing ABTS^•–^) and panel II (the LDH/NAD^+^ cascade reducing MB^+^ at time intervals of the transient transition CDN H → L → H): (i) *t* = 0, (ii) after 100 min, (ii) after 200 min, (iv) after 300 min, (v) after 400 min, (vi) after 600 min. (C) Transient orthogonal catalytic rates corresponding to the orthogonal cascades GOx/HRP and LDH/NAD^+^ upon the dynamic L_1_′-triggered reconfiguration of CDN H → L → H: panel I, the GOx/HRP cascade; panel II, the LDH/NAD^+^ cascade. Curves a, b, c, d (dots), experimental results; solid curves a′, b′, c′, d′, computationally simulated results. Curves a/a′ and c/c′ were recorded in the presence of L_1_′ = 12 μM and involved computational simulation of the experimental results. Curves b/b′ and d/d′ were recorded in the presence of L_1_′ = 8 μM, first computationally predicted followed by experimental validation. (D) Transient concentration of constituents: panel I (BB′) and panel II (CD′) upon the L_2_′-triggered reconfiguration of CDN H → K → H. (E) L_2_′ (12 μM)-triggered time-dependent absorbance changes generated by panel I (the GOx/HRP cascade oxidizing ABTS^•–^) and panel II (the LDH/NAD^+^ cascade reducing MB^+^ at time intervals of the transient transition CDN H → K → H): (i) *t* = 0, (ii) after 100 min, (ii) after 200 min, (iv) after 300 min, (v) after 400 min, (vi) after 600 min. (F) Transient orthogonal catalytic rates corresponding to the orthogonal cascades GOx/HRP and LDH/NAD^+^ upon the dynamic L_2_′-triggered reconfiguration of CDN H → L → H: panel I (the GOx/HRP cascade); panel II (the LDH/NAD^+^ cascade). Curves (a, b, c, d) (dots), experimental results; solid curves a′, b′, c′, d′, computationally simulated results. Curves a/a′ and c/c′ were recorded in the presence of L_2_′ = 12 μM and involved computational simulation of the experimental results. Curves b/b′ and d/d′ were recorded in the presence of L_2_′ = 8 μM, first computationally predicted followed by experimental validation. Error bars are derived from *N* = 4 experiments.

Figure 4A, curves (dots), depicts the transient concentration changes of the constituents BB′ (panel I) and CD′ (panel II) transduced by the DNAzyme reporter units upon the L_1_′-triggered transient transition of CDN H → L → H. The constituent BB′ is downregulated for 70 min followed by a concentration increase along a time interval of ca. 400 min, after which the original concentration of BB′ in “rest” CDN H was recovered. Within this time scale constituent CD′ undergoes orthogonal transient concentration changes and initially reveals a temporal increase of concentration for ca. 70 min, followed by a temporal decrease of its concentration to the initial concentration in “rest” CDN H, over a time interval of ca. 400 min. The orthogonal transient concentrations of the constituents BB′ and CD′ are reflected by orthogonal effects on the temporal biocatalytic transformations of the biocatalytic scaffolds associated with the constituents, Figure 4B. The temporal absorbance changes generated upon the oxidation of ABTS^2–^ to ABTS^•–^ within the samples withdrawn at time intervals from the reaction mixture, following the temporal upregulation of the GOx/HRP cascade, are displayed in Figure 4B, panel I. The concomitant transient absorbance changes generated by the LDH/NAD^+^ cascade and transduced by the temporal absorbance changes of MB^+^ reduction to MBH by samples withdrawn, at time intervals, from the system are displayed in Figure 4B, panel II. Clearly, the L_1_′-triggered dynamical reconfiguration of CDN H to CDN L leads to the orthogonal transient perturbation of the efficiency of the GOx/HRP and LDH/NAD^+^ cascades, Figure 4C, panel I and panel II, curves a and c, dots, respectively. Figure 4D, panel I and II, curves (dots), presents the transient concentration of the constituent BB′, bearing the GOx/HRP catalysts, and of constituent CD′, to which the LDH/NAD^+^ biocatalyst is tethered, upon the L_2_′-triggered transition of CDN H → K → H. Orthogonal transient temporal concentration changes for the two constituents are observed. While the constituent BB′ is upregulated and recovered to the parent concentration in CDN H, the constituent CD′ is downregulated and recovered to its original values. The orthogonal transient concentration changes of constituents BB′ and CD′ are reflected in the corresponding biocatalytic cascades of the biocatalysts linked to the two constituents, Figure 4E, panels I and II. While the temporal oxidation of ABTS^2–^ to ABTS^•–^ by samples withdrawn at time intervals from the system reveals upregulation of the GOx/HRP cascade, followed by recovery of the original GOx/HRP cascade in CDN H, the LDH/NAD^+^ cascade reveals orthogonal behavior, and the temporal reduction of MB^+^ to MBH by samples withdrawn at time intervals from the system reveals downregulation of the LDH/NAD^+^ cascade followed by recovery of the parent biocatalytic activity characterizing CDN H. In Figure 4F, panels I and II, curves a and c (dots), correspond to the orthogonal transient catalytic rates of the cascades GOx/HRP and LDH/NAD^+^ upon the dynamic reconfiguration of CDN H → K → H. The kinetics of the L_1_′/L_2_′-triggered orthogonally operating biocatalytic cascades were computationally simulated, Figure S28 and Figure S29. As the composite of CDN H and its transient reconfiguration of CDN H → L → H or CDN H → K → H include the same rate constants of the operating equations in the separated CDN X or O, we adapted the derived rate constants for the separated CDNs to predict the transient concentrations of the constituents in the orthogonally operating modules (for the rate constants, see Tables S6 and S7). The resulting predicted transient concentrations of the constituents (solid lines) are presented in Figure 4A and D, and these fit well with the experimental results (curves consisting of dots). Also, the catalytic rates evaluated for the separated CDNs X and O were adapted to predict the catalytic rates of the orthogonally operating biocatalytic cascades. The resulting predicted catalytic rates of the orthogonally operating cascades upon the L_1_′-triggered transient transition of CDN H → L → H and L_2_′-triggered transient transition of CDN H → K → H are overlaid on the experimental catalytic rates (curves a and c, dots), Figure 4C and F, curves a′ and c′, respectively. Very good fitting between the computational catalytic rates and the experimental data is observed. While the experimental and computational results presented in curves a′/c′ correspond to the system triggered by L_1_′/L_2_′ at concentrations corresponding to 12 μM, we probed the versatility of the kinetic model at L_1_′/L_2_′ concentrations corresponding to 4 μM. For the time-dependent absorbance changes of ABTS^•–^ and MBH generated by the GOx/HRP and LDH/NAD^+^ cascades in the L_1_′/L_2_′ (8 μM)-triggered transient transitions of CDN H → L → H or CDN H → K → H, see Figures S30 and S31. The computationally predicted orthogonal transient catalytic rates for the GOx/HRP and LDH/NAD^+^ cascades were derived, Figure 4C and F, solid curves b′/d′, and these were experimentally validated, curves b/d (dots). Very good agreement between the predicted and experimental results is demonstrated.

**Figure 5:**
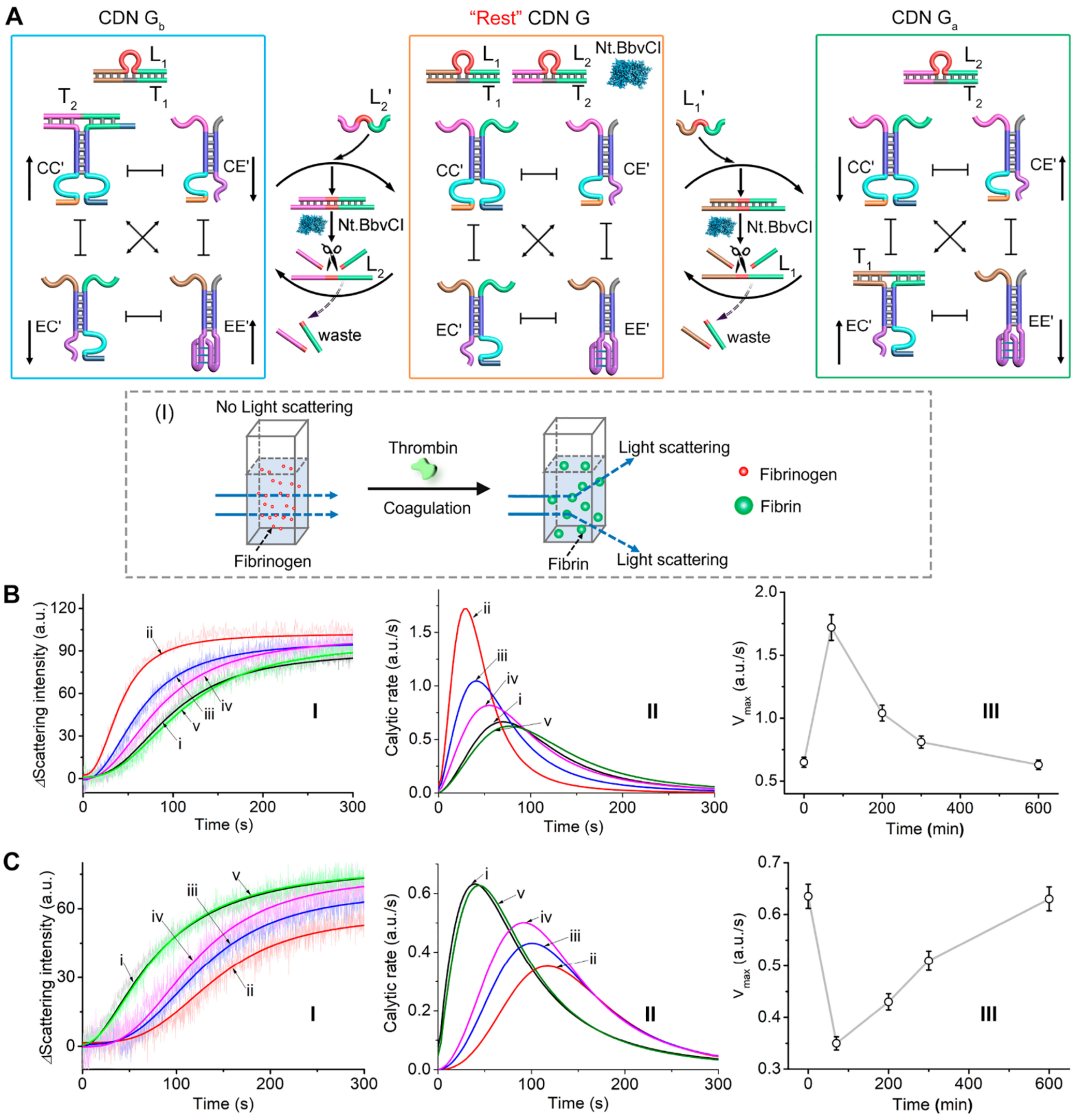
(A) Constitutional dynamic network-guided directional and orthogonal transient reconfiguration of a CDN G network, composed of an anti-thrombin aptamer constituent inhibiting the catalytic fibrinogen coagulation functions of thrombin. The dynamic transient reconfiguration of CDN G to CDN G_a_ or CDN G_b_ leads to transient downregulation or upregulation of the catalytic functions of thrombin. Inset I: Schematic probing catalytic functions of thrombin by temporal light-scattering experiments following the coagulation of fibrinogen to fibrin.^61^ (B) Panel I: Temporal light-scattering intensity changes corresponding to the temporal coagulation of fibrinogen to fibrin upon the dynamic transition of CDN G to CDN G_a_ and back. Light-scattering curves are recorded on samples withdrawn from the system at time intervals corresponding to (i) 0 min, (ii) 70 min, (iii) 200 min, (iv) 300 min, and (v) 600 min. Panel II: Catalytic rates corresponding to the coagulation of fibrinogen to fibrin at time intervals operating the transition of CDN G → CDN G_a_ → CDN G. Panel III: Transient maximum catalytic rates demonstrated by the L_1_′-triggered transition of CDN G → CDN G_a_ → CDN G. (C) Panel I: Temporal light-scattering intensity changes corresponding to the temporal coagulation of fibrinogen to fibrin upon the dynamic transition of CDN G to CDN G_b_ and back. Light-scattering curves are recorded on samples withdrawn from the system at time intervals corresponding to (i) 0 min, (ii) 70 min, (iii) 200 min, (iv) 300 min, and (v) 600 min. Panel II: Catalytic rates corresponding to the coagulation of fibrinogen to fibrin at time intervals operating the transition of CDN G → CDN G_b_ → CDN G. Panel III: Transient minimum catalytic rates demonstrated by the L_2_′-triggered transition of CDN G → CDN G_b_ → CDN G. Error bars are derived from *N* = 4 experiments.

To address the potential utility of a dynamic DNA network exhibiting directional and orthogonal transient biocatalytic functions, we designed a network demonstrating the dynamic upregulation or downregulation of transient catalytic activities of thrombin toward the coagulation of fibrinogen, Figure 5. The parent constitutional dynamic network, “rest” CDN G, consists of four constituents CC′, CE′, EC′, and EE′, where constituent EE′ includes components E and E′ tethered to G-quadruplex subunits being parts of the thrombin aptamer. Thus, the constituent EE′ acts as the active component that binds to thrombin and inhibits its catalytic coagulation activities based on its concentration.^61,62^ The two duplexes L_1_/T_1_ and L_2_/T_2_ and the nicking enzyme Nt.BbvCI are added as auxiliary components to CDN G. Subjecting CDN G to trigger L_1_′ separates the duplex L_1_/T_1_ to form L_1_/L_1_′, while releasing strand T_1_ that is engineered to hybridize with constituent EC′. This results in the dynamic reconfiguration of CDN G to CDN G_a_, where EC′ and CE′ are upregulated and concomitantly CC′ and EE′ are downregulated. The downregulation of EE′ results in a network with low catalytic inhibition capacity toward thrombin, and thus, the thrombin-stimulated capacity to induce the coagulation of thrombin is faster in CDN G_a_ as compared to CDN G. The sequence encoded in the strand L_1_′ was engineered, however, to be nicked by Nt.BbvCI in the duplex structure L_1_/L_1_′, resulting in the release of L_1_. The released L_1_ separates the strand T_1_ from the constituent EC′, resulting in the transient recovery of CDN G_a_ into CDN G and the transient restoration of the catalytic inhibition properties of constituent EE′ toward thrombin (enhanced inhibition). Figure 5B, panel I, presents the temporal coagulation rates of sample withdrawn from the L_1_′-triggered activation of CDN G. While at *t* = 0 min the system reveals lower thrombin-stimulated fibrinogen coagulation rates, curve i (due to higher contents of EE′), subjecting the system to L_1_′ and reconfiguration of CDN G to CDN G_a_ enhances the rate of coagulation, curve ii, due to the lowering of the content of EE′. Subsequently, a temporal decrease in the coagulation rates of fibrinogen by thrombin is observed in curves iii–v due to the transient depletion of CDN G_a_ and the recovery of CDN G. Figure 5B, panel II, presents the catalytic rates corresponding to the temporal coagulation rates of fibrinogen by the dynamic system (derivatives of the temporal curves shown in panel I). In Figure 5B, panel III shows the peak values of the catalytic rates of the coagulation process upon the transient transition of the network across CDN G → CDN G_a_ → CDN G. Similarly, triggering CDN G with strand L_2_′ leads to the separation of the duplex L_2_/T_2_ to form L_2_/L_2_′ and the free strand T_2_. Accordingly, the strand T_2_ hybridizes with constituent CC′ of CDN G, resulting in the dynamic reconfiguration of CDN G to CDN G_b_, where constituents CC′ and EE′ are upregulated and constituents CE′ and EC′ are downregulated. The upregulation of EE′ leads to the enhanced catalytic inhibition capacities toward the coagulation fibrinogen of CDN G_b_ as compared to the inhibiting capacities of CDN G (due to the higher content of the inhibiting G-quadruplex aptamer). The nickase-stimulated cleavage of L_2_′ in the duplex L_2_/L_2_′ releases L_2_, which displaces T_2_, resulting in the transient recovery of CDN G_b_ to CDN G. In Figure 5C, panel I, curve i, shows the rate of fibrinogen coagulation at *t* = 0 min, by CDN G. Subjecting the CDN G to L_2_′ leads to a substantially lower rate of thrombin coagulation, curve ii, due to the reconfiguration of CDN G to CDN G_b_, enriching EE′. Subsequently, the nickase-stimulated recovery of CDN G_b_ to CDN G leads to the temporal enhancement of the coagulation rates demonstrated by the dynamic system, consistent with the recovery of CDN G. In Figure 5C, panel II shows the temporal catalytic rates demonstrated by CDN G subjected to L_2_′, and panel III in Figure 5C depicts the transient catalytic rates corresponding to the L_2_′-triggered transition of CDN G → CDN G_b_ → CDN G. It should be noted that the dynamically controlled coagulation of fibrinogen by thrombin is controlled by the relative concentrations of the constituent EE′ in the respective networks. Realizing, however, that the concentration of EE′ is dominated by the concentration of the agonist constituent CC′, we were able to probe the dynamic concentration changes of constituent CC′ upon the transitions of CDN G → CDN G_a_ → CDN G and CDN G → CDN G_b_ → CDN G; see Figure S32 and accompanying discussion, Supporting Information. In addition, the CDNs bearing the thrombin aptamer subunits were used as a sensing platform to probe the thrombin as a practical application, Figure S33. These results demonstrate the orthogonal dynamic and transient dose-controlled inhibition of the thrombin fibrinogen coagulation process by the dynamic network.

## Conclusions

The present study has introduced concepts to couple dynamic reconfigurable constitutional dynamic networks and the transient temporal operation of the reconfigured scaffolds. Specifically, by tethering biocatalytic units to the constituents of the reaction modules and driving the dynamic reconfiguration and transient operation of the frameworks, transient temporal operation of the biocatalytic cascades was accomplished. These were demonstrated for two different biocatalytic cascades, GOx/HRP and LDH/NAD^+^. By applying two different triggers, L_1_′ and L_2_′, the directionally dictated upregulated or downregulated operation of each of the biocatalytic cascades was demonstrated. In addition, by integration of the two CDNs, driving the GOx/HRP and LDH/NAD^+^ cascades, into a united reaction module, the L_1_′/L_2_′-driven dynamic orthogonal operation of the two biocatalytic cascades and the dictated directional operation of the biocatalytic cascades were achieved. Moreover, the concept of orthogonal upregulation and downregulation of temporal biocatalytic processes using transient CDNs as control frameworks was applied to design the temporal upregulation/downregulation of the thrombin-stimulated coagulation of fibrinogen, thus providing a potential medical application. In fact, this concept can be broadened further for other temporal, transient, dose-controlled activation of other therapeutic agents by dynamic CDNs. Beyond the basic concepts and principles employing complex dynamic and transient DNA networks as frameworks controlling biocatalytic transformations, the systems provide artificial models for native networks-guided dynamic and transient biocatalytic transformations. While the bottom-up design of the nucleic acid/enzyme networks seems like a possible limitation, particularly due to cross-interactions between the nucleic acid strands and limited stability due to enzyme denaturation and nucleic acid enzymatic digestions, we feel that nucleic acids provide significant properties and functions to further advance the field. The structural and functional information encoded in the base sequence of nucleic acid provides a plethora of design principles and diverse structural framework complexities that are not available with any other synthetic framework. We also believe that the future synthetically modified nucleic acids and the integration of synthetic catalysts, instead of enzymes, could overcome the present limitations of the native biomolecules. Furthermore, the present study used two duplexes, L_1_/T_1_ and L_2_/T_2_, and two CDNs to control two biocatalytic processes, yet designing systems of enhanced complexity is envisaged. For example, by integrating three duplexes, L_1_/T_1_, L_2_/T_2_, and L_3_/T_3_, and appropriate triggers, three branched biotransformations consisting of catalytic cascades could be designed, and further employing pairs of L_1_/T_1_, L_2_/T_2_, and L_3_/T_3_, L_4_/T_4_, the biorthogonality of four different biotransformations could be realized.

## Experimental Section

The nucleic acid sequences used in the study were obtained commercially (Integrated DNA Technologies, IDT), and the respective sequuences were as follows:

A: 5′-GATATCAGCGATCAAAATACTTACAGACACAACAA-3′

A′: 5′-ACAGAAGAACCGTAAGTATTTTGCACCCATGTTACTCT-3′

B: 5′-CTGCTCAGCGATCAAAATACTTACCCCATCACAAA AATTT-SH-3′

B′: 5′-SH-AAGTAAGTATTTTGCACCCATGTTCGTCA-3′

C: 5′-CTGTTCAGCGATCAAACTAATTACAGACACAACAAA-NH_2_-3′

C′: 5′-ACAGAAGAACCGTAATTAGTTTGCACCCATGTTTCAGT-3′

D: 5′-GTCCTCAGCGATCAAACTAATTACCCCATCACAAAAATTT-3′;

D′: 5′-SH-AAGTAATTAGTTTGCACCCATGTTCCTGA-3′

E: 5′-GGTTGGTCAAACTAATTACCCCATCACAAAAATTT-3′

E′: 5′-AAGTAATTAGTTTGGTGGTTGG-3′

T_1_: 5′-AAAGGTTTGTGATGGACGTTCTTCTGTC-3′

L_1_: 5′-GACAGAAGAACGCTGAGGCCATCACAAACC-3′

L_1_^M^: 5′-GACAGAAGAACGGCGTAGCCATCACAAACC-3′

L_1_′: 5′-GATGGCCTCAGCGTT-3′

T_2_: 5′-CGTTGTTGTGTCACGTTCTTCTGTG-3′

L_2_: 5′-CACAGAAGAACGCTGAGGGACACAACAACG-3′

L_2_^M^: 5′-CACAGAAGAACGGCGTAGGACACAACAACG-3′

L_2_′: 5′-GTGTCCCTCAGCGTT-3′

F_cali_: 5′-Cy5-TCGTCCTCAGCT-3′

Q_cali_: 5′-AGCTGAGGACGA-BHQ2-3′

sub1 (AA′): 5′-FAM-AGAGTATrAGGATATC-BHQ–3′

sub2 (BB′): 5′-ROX-TGACGATrAGGAGCAG-BHQ2-3′

sub3 (BA′): 5′-Cy5-AGAGTATrAGGAGCAG-BHQ2-3′

sub4 (AB′): 5′-Cy5.5-TGACGATrAGGATATC-IBRQ-3′

sub5 (DC′): 5′-FAM-ACTGAATrAGGAACAG-BHQ1-3′

sub6 (CD′): 5′-ROX-TCAGGATrAGGAGGAC-BHQ2-3′

sub7 (CC′): 5′-Cy5-ACTGAATrAGGAGGAC-BHQ2-3′

sub8 (DD′): 5′-Cy5.5-TCAGGATrAGGAACAG-IBRQ-3′

sub1-noFQ (AA′): 5′-AGAGTATrAGGATATC-3′

sub2-noFQ (BB′): 5′-TGACGATrAGGAGCAG-3′

sub3-noFQ (BA′): 5′-AGAGTATrAGGAGCAG-3′

sub4-noFQ (AB′): 5′-TGACGATrAGGATATC-3′

sub5-noFQ (DC′): 5′-ACTGAATrAGGAACAG-3′

sub6-noFQ (CD′): 5′-TCAGGATrAGGAGGAC-3′

sub7-noFQ (CC′): 5′-ACTGAATrAGGAGGAC-3′

sub8-noFQ (DD′): 5′-TCAGGATrAGGAACAG-3′

The detailed preparations of single modified conjugates of nucleic acid/enzyme or cofactor (B-GOx; B′-HRP; D′-LDH; C-NAD^+^), time-dependent absorbance changes of ABTS^•-^ and MB^+^ generated by the reconfigured CDNs in the presence of the different triggers, and the computational kinetic models for the systems are presented in the Supporting Information.

## Supporting Information Available

Materials, characterizations, methods, calibration curves, time-dependent fluorescence changes of the constituents during the transient of reconfiguration of CDNs, computational kinetic models, and additional results (PDF)
