# Supplementary material for: Dynamic DNA Networks-Guided Directional and Orthogonal Transient Biocatalytic Cascades
Source: J Am Chem Soc. 2023 Sep 29;145(40):22135–49. doi: 10.1021/jacs.3c08020 (PMC10571085; doi:10.1021/jacs.3c08020)
Supplement: Supplementary file 1 — ja3c08020_si_001.pdf [file ja3c08020_si_001.pdf]

Supporting Information

Dynamic DNA Networks-Guided Directional and Orthogonal Transient Biocatalytic Cascades

Yu Ouyang, Jiantong Dong and Itamar Willner*

The Institute of Chemistry, Center for Nanoscience and Nanotechnology, The Hebrew University of Jerusalem, Jerusalem 91904, Israel.

Materials and Methods

Materials

Glucose oxidase from *Aspergillus niger* (GOx, 187300 U g^{-1}), peroxidase from horseradish, type II (HRP, 219000 U g^{-1}), nicotinamide adenine dinucleotide (NAD^+), succinimidyl 3-(2-pyridyldithio)propionate (SPDP), 4-carboxyphenylboronic acid, 1-ethyl-3-(3-dimethylaminopropyl)carbodiimide (EDC), N-hydroxysulfosuccinimide (NHS), lactate dehydrogenase from *rabbit muscle* (LDH, 550 U mg^{-1}), alanine dehydrogenase, recombinant, expressed in *E. coli* (AlaDH, 21 U mg^{-1}), pyruvic acid, glucose, 2,2′-azino-bis(3-ethylbenzothiazoline-6-sulfonic acid) ($ABTS^{2-}$), methylene blue (MB^+), hydrazine hydrate, and lactic acid were purchased from Sigma-Aldrich. rCutSmart Buffer was purchased from New England BioLabs Inc.. DNA oligonucleotides were purchased from Integrated DNA Technologies Inc.

Instrumentation

Absorption spectra were recorded at 25 °C using a UV-2450 spectrophotometer (Shimadzu), a cuvette of 50 μL volume (made of quartz suprasil, Hellma Analytics) was used in these experiments. Fluorescence spectra were recorded at 25 °C using a Cary Eclipse Fluorometer (Varian Inc), a cuvette of 100 μL volume (disposable cuvette, Brand GMBH, Wehrheim, Germany) was used in these experiments. The enzyme/cofactor-DNA conjugates purification were implemented by anion-exchange fast protein liquid chromatography (FPLC) with MonoQ HR 5/5 column (GE Healthcare, 5 × 50 mm, 1 mL bearing sample volume).

Methods

Modification of strand B with GOx

GOx (80 μl, 100 μM) was subjected to SPDP (2.4 μL, 0.01 M) in HEPES buffer (10 mM, pH = 7.2) for 1 h. Excess SPDP was removed by washing three times with Amicon 10 kDa cutoff filters. Next, SPDP-modified GOx was conjugated to strand B (eightfold excess) through a disulfide bond exchange of the activated pyridyldithiol group. The reaction mixture was stirred in HEPES buffer (10 mM, pH = 7.2) for 2 h. The coupling efficiency was evaluated by monitoring the increase in absorbance at 343 nm due to the release of pyridine-2-thione (extinction coefficient, 8,080 M^{-1} cm^{-1}). Finally, the excess DNA was removed by washing with Amicon 30 kDa cutoff filters. The DNA labelling ratio of the purified enzyme-DNA conjugates was also quantified by measuring the absorbance ratio at 260 and 280 nm. The enzymatic activity of the DNA-modified GOx was ~71% of the activity of the native enzyme.

Modification of strand B′ with HRP

HRP (80 μl, 100 μM) was subjected to SPDP (5.6 μL, 0.01 M) in HEPES buffer (10 mM, pH = 7.2) for 1 h. Excess SPDP was removed by washing three times with Amicon 10 kDa cutoff filters. Next, SPDP-modified HRP was conjugated to strand B′ (eightfold excess) through a disulfide bond exchange of the activated pyridyldithiol group. The reaction mixture was stirred in HEPES buffer (10 mM, pH = 7.2) for 2 h. The coupling efficiency was evaluated by monitoring the increase in absorbance at 343 nm due to the release of pyridine-2-thione (extinction coefficient, 8,080 M^{-1} cm^{-1}). Finally, the excess

DNA was removed by washing with Amicon 30 kDa cutoff filters. The DNA labelling ratio of the purified enzyme-DNA conjugates can also be quantified by measuring the absorbance ratio at 260 and 280 nm. The enzymatic activity of the DNA-modified HRP was ~63% of the activity of the native enzyme.

Modification of strand D′ with LDH

LDH (100 μl, 23 μM) was subjected to SPDP (4 μL, 0.5 M) in HEPES buffer (10 mM, pH = 8.0) for 1 h. Excess SPDP was removed by washing three times with Amicon 10 kDa cutoff filters. Next, SPDP-modified LDH was conjugated to strand D′ (eightfold excess) through a disulfide bond exchange of the activated pyridyldithiol group. The reaction mixture was stirred in HEPES buffer (10 mM, pH = 7.2) for 2 h. The coupling efficiency was evaluated by monitoring the increase in absorbance at 343 nm due to the release of pyridine-2-thione (extinction coefficient, 8,080 M^{-1} cm^{-1}). Finally, the excess DNA was removed by washing with Amicon 30 kDa cutoff filters. The DNA labelling ratio of the purified enzyme-DNA conjugates can also be quantified by measuring the absorbance ratio at 260 and 280 nm. The enzymatic activity of the DNA-modified LDH was ~67% of the activity of the native enzyme.

Modification of strand C with NAD^+

4-carboxyphenylboronic acid (2 μL, 50 mM) was subjected to EDC (3 μL, 10 mg mL^{-1}) in 200 μL of MES buffer (10 mM, pH = 5.5) for 5 min. Subsequently, NHS (5 μL, 10 mg mL^{-1}) in 200 μL of PBS buffer (50 mM, pH = 7.2) was added and the mixture shaken for 10 min. Strand C (10 μL, 1 mM) was then added and the mixture shaken for 2 ~ 3 h. Excess reagents were removed by washing three times with Amicon 3 kDa cutoff filters. Finally, the resulting solution was reacted with NAD^+ (10 μL, 10 mM) in PBS buffer (50 mM, pH = 10) for 2 h at room temperature and then overnight at 4 ℃. C-NAD^+ was obtained after washing with Amicon 3 kDa several times. For the characterization of the single nucleic acid-modified cofactor, see Supporting Information, Figure S7.

Enzyme/cofactor-DNA conjugate purification by anion-exchange FPLC

The enzyme/cofactor-DNA conjugates were diluted three times with wash buffer (20 mM phosphate buffer, pH 7.4, containing 25 mM Na^+) and were injected into the column. Then, the samples were separated by using an elution gradient from 0.25 mM Na^+ (20 mM phosphate buffer, pH 7.4) to 1 M Na^+ (20 mM phosphate buffer, pH 7.4) with a flow rate of 1.5 mL/min. The fractions of multiple peaks from the purification chromatogram were collected and analyzed by UV spectra to evaluate the ratio of enzyme and DNA.

Preparation of CDN X, CDN O and CDN H

A mixture of A, B, A′, and B′, 2 μM each, in 1× rCutSmart Buffer, was annealed at 65 °C for 10 min, and slow cooling to 25 °C at a rate of 0.3 °C min^{-1}, and then allowed to equilibrate at 25 °C for 2 h. The prepared mixture (400 μL) was subjected to the Nt.BbvCI nicking enzyme, 12 μL of 2.3 μM, T_1, T_2, L_1, and L_2, 8 μL of 100 μM, and

136 μL 1 × CutSmart Buffer, to yield CDN X including each component, 1 μM, T_1L_1 and T_2L_2, 1 μM each, and Nt.BbvCI, 0.0345 μM.

A mixture of C, D, C′, and D′, 2 μM each, in 1× rCutSmart Buffer, was annealed at 65 °C for 10 min, and slow cooling to 25 °C at a rate of 0.3 °C min^{-1}, and then allowed to equilibrate at 25 °C for 2 h. The prepared mixture (400 μL) was subjected to the Nt.BbvCI nicking enzyme, 12 μL of 2.3 μM, T_1, T_2, L_1, and L_2, 8 μL of 100 μM, and 136 μL 1 × CutSmart Buffer, to yield CDN O including each component, 1 μM, T_1L_1 and T_2L_2, 1 μM each, and Nt.BbvCI, 0.0345 μM.

A mixture of A, B, A′, B′, C, D, C′, and D′, 2 μM each, in 1× rCutSmart Buffer, was annealed at 65 °C for 10 min, and slow cooling to 25 °C at a rate of 0.3 °C min^{-1}, and then allowed to equilibrate at 25 °C for 2 h. The prepared mixture (400 μL) was subjected to the Nt.BbvCI nicking enzyme, 24 μL of 2.3 μM, T_1, T_2, L_1, and L_2, 16 μL of 100 μM, and 128 μL 1 × CutSmart Buffer, to yield CDN H including each component, 1 μM, T_1L_1 and T_2L_2, 2 μM each, and Nt.BbvCI, 0.0345 μM.

Note that the nicking enzyme was purified to remove dithiothreitol contained in storage buffer which may induce the breaking of disulfide bond, and a calibration curve was used for quantifying the concentration of the nicking enzyme, Figure S10.

Probing dissipative transitions of CDN X, CDN O and CDN H by using the DNAzyme reporter units

Taking the L_1'-triggered transient reconfiguration of CDN X→Y→X as an example. All other transient reconfigurations of the CDN X, CDN O and CDN H by triggers L_1' or L_2' are similarly followed.

For the dissipative transitions of CDN X→Y→X, CDN X (400 μL) was subjected to the fuel strand L_1', 5.82 μL of 500 μM, and allowed to equilibrate at 37 °C. Aliquots of 30 μL were withdrawn from the solution at different time-intervals and treated with sub1, sub2-noFQ, sub3-noFQ, and sub4-noFQ (for AA′), or with sub1-noFQ, sub2, sub3-noFQ, and sub4-noFQ (for BB′), or with sub1-noFQ, sub2-noFQ, sub3, and sub4-noFQ (for BA′), or with sub1-noFQ, sub2-noFQ, sub3-noFQ, and sub4 (for AB′), 1.5 μL of 100 μM each. Subsequently, the time-dependent fluorescence changes were followed at 25 °C (catalytic rate = $d(\Delta F)/dt$). By using the appropriate calibration curves corresponding to the cleavage rates of the different substrates by the intact constituents at various concentrations, the concentrations of the constituents in transient transition of CDN X→Y→X at different time intervals were quantified. For the dissipative transitions of CDN X→Z→X, the fuel strand L_2' was used instead of the fuel strand L_1'.

Transient GOx/HRP cascade in L_1'/L_2'-triggered transient transition of CDN X

To follow the GOx/HRP biocatalytic cascade, the equilibrated CDN X (400 μL, each component 1 μM) was subjected to the fuel strand L_1'/L_2' (6 μM or 4 μM), and allowed to equilibrate at 37 °C. Aliquots of 60 μL were withdrawn from the above L_1'/L_2'-triggered CDN X solution at different time-intervals and treated with glucose (1 μL, 2.4 mM) and $ABTS^{2-}$ (1 μL, 6 mM). The absorbance changes at 420 nm were intermediately measured (probing for 2 min).

Transient LDH/NAD$^+$ cascade in L_1'/L_2'-triggered transient transition of CDN O

To follow the LDH/NAD$^+$ biocatalytic cascade, the equilibrated CDN O (400 μL, each component 1 μM) was subjected to the fuel strand L_1'/L_2' (6 μM or 4 μM), and allowed to equilibrate at 37 °C. Subjecting the aliquots (60 μL) of the L_1'/L_2'-triggered CDN O to 1 mM lactic acid, 1 mM hydrazine, and 50 μM MB$^+$ with different time intervals allows us to probe the LDH/NAD$^+$ cascade by the time-dependent absorbance of reduction of MB$^+$ at λ = 664 nm (probing for 2 min).

Orthogonally operating transient GOx/HRP cascade and LDH/NAD$^+$ cascade in L_1'/L_2'-triggered transient transition of CDN H

To follow the orthogonally pertubed biocatalytic efficiencies of the GOx/HRP cascade and the LDH/NAD$^+$ biocatalytic cascade, the equilibrated CDN H (800 μL, each component 1 μM) was subjected to the fuel strand L_1'/L_2' (12 μM or 8 μM), and allowed to equilibrate at 37 °C. Aliquots of 60 μL were withdrawn from the above L_1'/L_2'-triggered CDN H solution at different time-intervals and treated with glucose (1 μL, 2.4 mM) and ABTS^{2-} (1 μL, 6 mM) followed by probing the absorbance changes at λ = 420 nm (probing for 2 min). Subjecting the aliquots (60 μL) of the L_1'/L_2'-triggered CDN H to 1 mM lactic acid, 1 mM hydrazine, and 50 μM MB$^+$ with different time intervals allows us to probe the LDH/NAD$^+$ cascade by the time-dependent absorbance of reduction of MB$^+$ at λ = 664 nm (probing for 2 min).

Preparation of CDN G and operating transient modulation of thrombin catalytic activity

A mixture of C, E, C′, and E′, 2 μM each, in 1× rCutSmart Buffer, was annealed at 65 °C for 10 min, and slow cooling to 25 °C at a rate of 0.3 °C min^{-1}, and then allowed to equilibrate at 25 °C for 2 h. The prepared mixture (400 μL) was subjected to the Nt.BbvCI nicking enzyme, 12 μL of 2.3 μM, T_1, T_2, L_1, and L_2, 8 μL of 100 μM, and 136 μL 1 × CutSmart Buffer, to yield CDN X including each component, 1 μM, T_1L_1 and T_2L_2, 1 μM each, and Nt.BbvCI, 0.0345 μM. To follow the temporal catalytic properties of the thrombin in CDN system, the equilibrated CDN G (800 μL, each component 1 μM) was subjected to the fuel strand L_1'/L_2' (6 μM), and allowed to equilibrate at 37 °C. Aliquots of 100 μL were withdrawn from the above L_1'/L_2'-triggered CDN H solution at different time-intervals and treated with thrombin (1 μL, 0.5 μM) and fibrinogen (5 μL, 10 mg/mL) followed by probing the scattering intensity at λ = 650 nm.

Purification of the GOx-B, HRP-B′, LDH-D′, NAD$^+$-C conjugates and the evaluation of the precise 1 : 1 molar ratio conjugation by FPLC and high-resolution mass spectra analyses.

1. The synthetic procedures to prepare the precise 1 : 1 molar ratio of enzyme : nucleic acid conjugates involved a common protocol and an identical purification protocol and validation process of the purity of conjugates:
   a. The enzymes were modified with SPDP to reach an approximate 1 : 1 molar functionalization yield that was estimated spectroscopically.

b. The resulting enzyme-SPDP conjugate was reacted with the respective thiolated nucleic acid to "yield" a 1 : 1 molar ratio conjugate. The resulting conjugate was spectroscopically analyzed to estimate a 1 : 1 molar yield functionalization by probing the relative absorbance intensities at λ = 260 nm and λ = 280 nm, respectively. Naturally, modification procedure might still yield a mixture of unmodified enzymes, accompanied by single-nucleic acid functionalized enzyme frameworks, accompanied by the traces of multiple nucleic acid modified enzyme units.
c. Accordingly, the mixture of nucleic acid modified enzymes was subjected to the FPLC chromatographic separation. The FPLC experiment were performed with gradient rise of sodium chloride concentration. The fraction containing the single nucleic acid modified enzyme was collected.
d. The collected single nucleic acid modified enzyme was reanalyzed by FPLC to confirm the single peak of the 1 : 1 modified, followed by spectroscopic analysis at λ = 260 nm and λ = 280 nm to confirm the 1 : 1 molar ratio.
e. The purified 1 : 1 molar ratio of nucleic acid : enzyme conjugate was further subjected to mass spectroscopy analysis while comparing the mass to bare parent enzyme.
f. In addition, the activity of the 1 : 1 nucleic acid-modified enzyme was compared to the native enzyme activities prior to modification.

Figure S3 depicts the experimental results that followed the purification of the HPR-B′ conjugate following the steps (a) to (d). Figure S4 shows the mass spectra analyses according to step (e).

Similar procedures were adapted for the characterization of GOx-B and LDH-D′ and the results are provided in Figure S2, Figure S5 and S6.

Table S8 summarizes the precise FPLC-purified enzyme : nucleic acid molar ratio derived by spectroscopic evaluation of the conjugate.

An procedure was adapted to synthesize the 1 : 1 NAD^+-C conjugate and to evaluate the purity of the conjugate:

a. DNA was reacted with 4-carboxyphenylboronic acid by the crosslinkers, (EDC) and (NHS), then linked to the NAD^+.
b. The synthesized NAD^+-C mixture was purified with 3 kDa Amicon filter to remove the redundant NAD^+.
c. Accordingly, the mixture of nucleic acid modified enzymes was subjected to the FPLC chromatographic separation. The FPLC experiment were formed and the fraction containing the single nucleic acid modified enzyme was collected, Figure S7A.

d. The separated NAD^+-C conjugate was subjected to the mass spectra analysis and compared to the mass of unmodified strand C, Figure S7B and S7C. The mass shift confirms the single 1 : 1 molar ratio of NAD^+-C.

Evaluation of the temporal concentration changes of the constituents upon the L_1'-triggerd transition of CDN X to CDN Y.

To further support the temporal concentration changes of the constituents upon the L_1'-triggered transition of CDN X to CDN Y by the DNAzyme reporter units, we performed complementary quantitative gel electrophoretic experiments. In these experiments, CDN X system composed of the constituents AA′, BB′, AB′, BA′ (with the enzyme labels) and L_1T_1 and the nickase was triggered with L_1' (samples with constant volume). A set of the CDNs was triggered for different time-intervals and samples from the temporal operating CDNs were deposited on the separating gel while ensuring that each deposited sample represents a real operation time of the transient CDN. The entire set of samples were separated on 12% native PAGE gel stained with Gel Red dye, and imaged by Gel Imager-ChemiDoc-Bio Rad. The separated constituents were compared to reference constituents of known concentrations to derive the concentrations of temporally generated separated constituents.

Figure S16 depicts, in lanes 1 to 8, the bands corresponding to the respective reference constituents (1 uM). In lane 9, the separated constituents at t = 0 (in absence of the trigger L_1') are presented. In lanes 10, 11 and 12, the separated bands corresponding to the L_1'-triggered constituents generated in the dynamic system after 70 minutes, 200 minutes, 600 minutes are presented, respectively. After 70 minutes, the strong band corresponding to $BA'T_1$ is observed (that is not present at t = 0) and the concomitant band corresponding to AB′ is intensified, while the non-separable bands AA′+BB′ are downregulated. Simultaneously, the band of T_1L_1 reveals lower intensity as compared to t = 0, and the appearance of a new band corresponding to $L_{1/}L_1'$. (Consistent with the separation of T_1L_1 and formation of $L_{1/}L_1'$). At longer time intervals, intensity changes of the separated bands AB′, $BA'T_1$, $L_{1/}L_1'$ decrease while the bands AA′+BB′, T_1L_1 intensities and after a time interval of 600 minutes the whole separation bands reveal the intensities observed at t = 0 (Consistent with the recovery of CDN X). Table S1 summarizes the temporal concentration of the respective constituents evaluated by the gel electrophoretic experiments in comparison to the temporal concentrations of the constituents determined by the DNAzyme reporter units. Good agreement between the two methods is observed, supporting the DNAzyme method to probe the concentration of the constituents in the different networks.

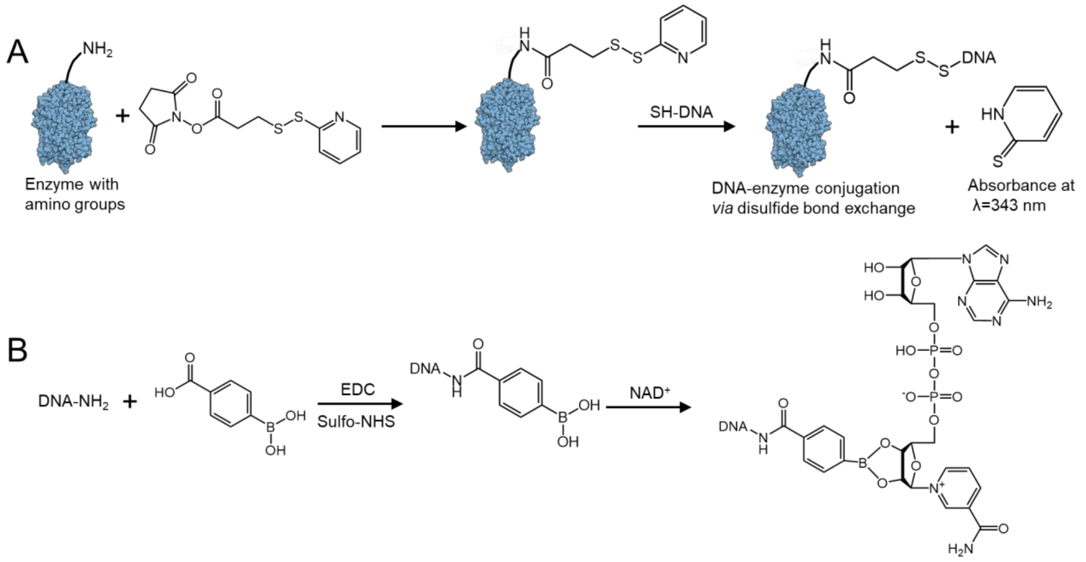

Figure S1. (A) Synthetic route to enzyme (GOx, HRP, LDH)-DNA conjugates. (B) Synthetic route to NAD^+-DNA conjugates.

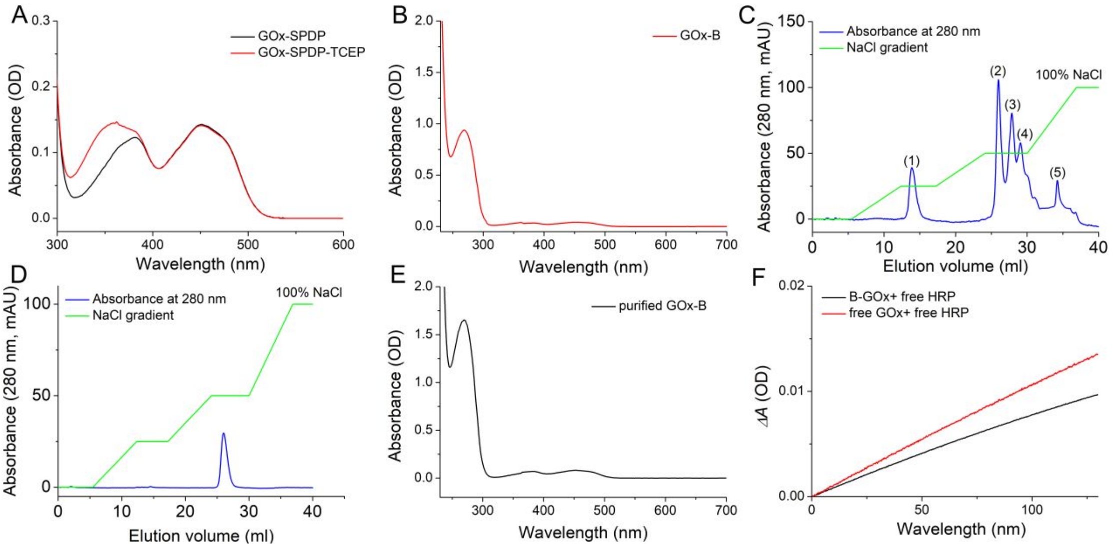

Figure S2. (A) Absorbance spectra of GOx-SPDP before and after the treatment of B: ΔA (λ = 343 nm) before and after the treatment of TCEP is ~ 0.062 (extinction coefficient of pyridine-2-thione: 8,080 M^{-1} cm^{-1}), corresponding to 7.67 μM SPDP coupled with 6.33 μM GOx (extinction coefficient of GOx at 450 nm is 22600 M^{-1} cm^{-1}). TCEP is used to calculate the coupling efficiency between GOx and SPDP due to the release of pyridine-2-thione. (B) The absorbance spectra of B-GOx conjugate after removing pyridine-2-thione and excess strand B with Amicon 30 kD cutoff filters. (C) Anion-exchange FPLC to purify DNA-conjugated GOx. The GOx and GOx modified with different strand B number are separated into distinct peaks that are collected in fractions: peak (1)-unmodified GOx; peak (2)-GOx labelled with one strand B; peak (3)-GOx labelled with two strand B; peak (4)-GOx labelled with three strand B; peak (5) unlabeled strand B. (D) Anion-exchange FPLC chromatogram output from collected fraction of peak (2) in (C). (E) The absorbance spectrum of purified B-GOx conjugate collected from peak (2) in anion-exchange FPLC spectrum. (F) The enzyme activity of B-GOx as compared to the native GOx by probing the generation of $ABTS^{\bullet-}$ in the presence of glucose (20 μM) and $ABTS^{2-}$ (100 μM) under identical concentration of each enzyme (0.5 μM).

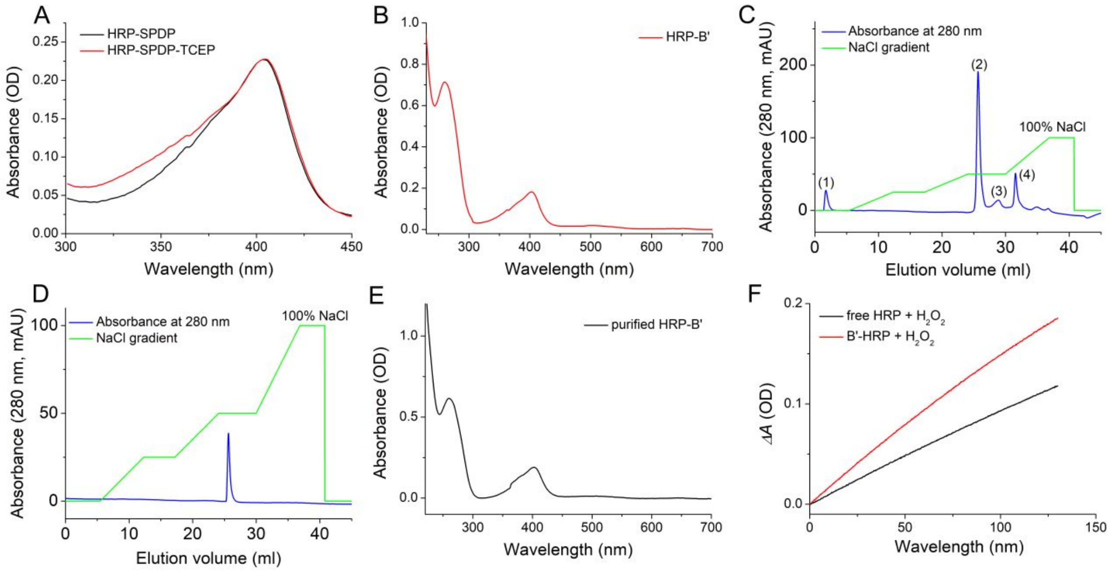

Figure S3. (A) Absorbance spectra of HRP-SPDP before and after the treatment of strand B′: ΔA ($\lambda = 343$ nm) before and after the treatment of TCEP is ~ 0.026 (extinction coefficient of pyridine-2-thione: 8,080 M^{-1} cm^{-1}), corresponding to 3.22 μM SPDP coupled with 2.28 μM HRP (extinction coefficient of HRP at 405 nm is 100000 M^{-1} cm^{-1}). TCEP is used to calculate the coupling efficiency between HRP and SPDP due to the release of pyridine-2-thione. (B) The absorbance spectra of B′-HRP conjugation after removing pyridine-2-thione and excess strand B′ with Amicon 30 kD cutoff filters. (C) Anion-exchange FPLC to purify DNA-conjugated HRP. The HRP and HRP modified with different strand B′ number are separated into distinct peaks that are collected in fractions: peak (1)-unmodified HRP; peak (2)-HRP labelled with one strand B; peak (3)-HRP labelled with two strand B′; peak (4)-unlabeled strand B′. (D) Anion-exchange FPLC chromatogram output from collected fraction of peak (2) in (C). (E) The absorbance spectrum of purified B′-GOx conjugate collected from peak (2) in anion-exchange FPLC spectrum. (F) The enzyme activity of B′-HRP as compared to the native HRP by probing the oxidation of ABTS^{2-}, in the presence of H_2O_2 (10 μM) and ABTS^{2-} (50 μM) under identical concentration of each enzyme (0.3 μM).

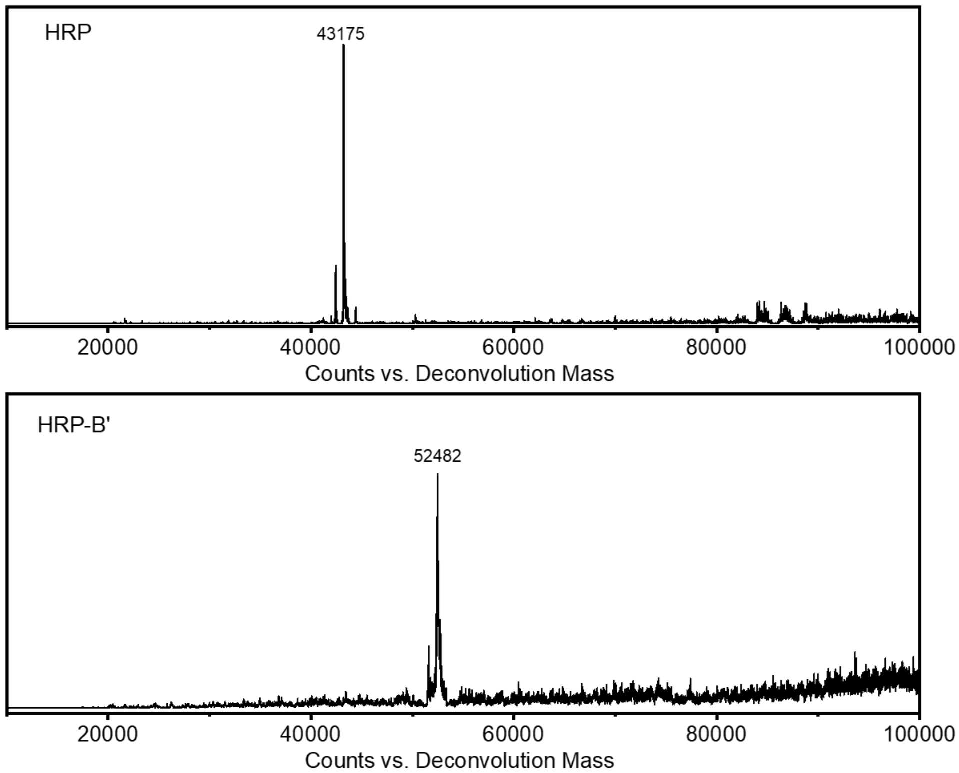

Figure S4. Transformed mass spectra by deconvolution of bare HRP and purified conjugate HRP-B′.

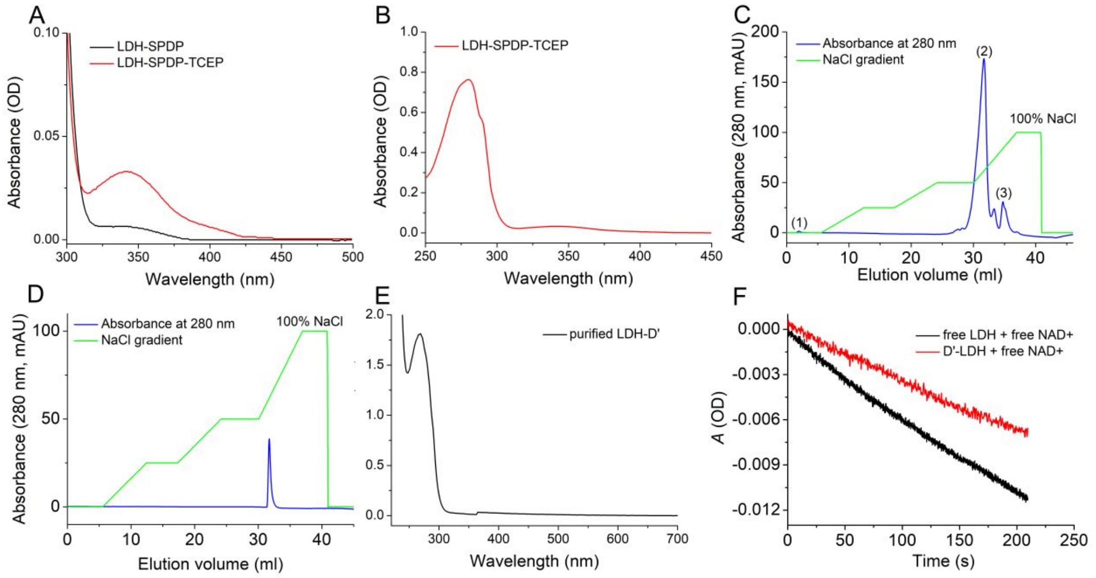

Figure S5. (A-B) Absorbance spectra of LDH-SPDP before and after the treatment of TCEP: ΔA (λ = 343 nm) before and after the treatment of TCEP is ~0.0265 (extinction coefficient of pyridine-2-thione: 8,080 M^{-1} cm^{-1}), corresponding to 3.28 μM SPDP coupled with 3.91 μM LDH (extinction coefficient of LDH at 280 nm is 186,502 M^{-1} cm^{-1}). The ratio of LDH: SPDP is 1.19. TCEP is used to calculate the coupling efficiency between LDH and SPDP due to the release of pyridine-2-thione. (C) Anion-exchange FPLC to purify DNA-conjugated LDH. The LDH and LDH modified with different strand D′ number are separated into distinct peaks that are collected in fractions: peak (1)-unmodified LDH; peak (2)-LDH labelled with one strand D′; peak (3)-unlabeled strand D′. (D) Anion-exchange FPLC chromatogram output from collected fraction of peak (2) in (C). (E) The absorbance spectrum of purified LDH-D′ conjugate collected from peak (2) in anion-exchange FPLC spectrum. (F) The enzyme activity of strand D′-modified lactate dehydrogenase (D′-LDH) as compared to the native LDH by probing the reduction of MB^+, in the presence of lactic acid (1 mM), hydrazine (1 mM), NAD^+ (0.2 μM) under the identical concentration of LDH (0.2 μM).

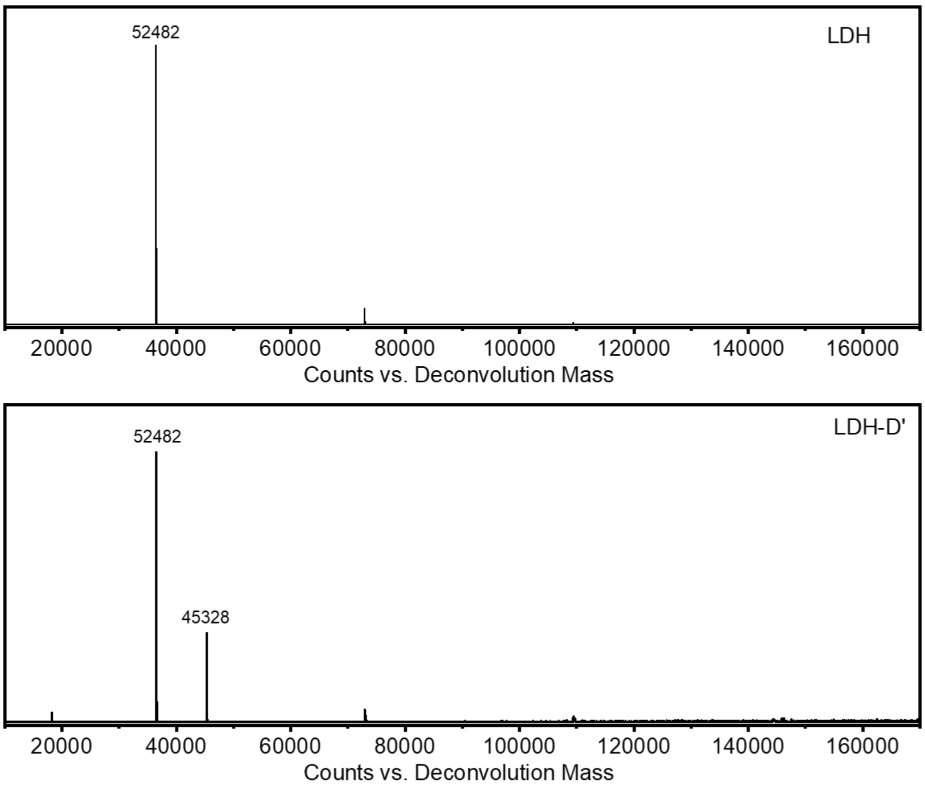

Figure S6. Transformed mass spectra by deconvolution of bare LDH and purified conjugate LDH-D′.

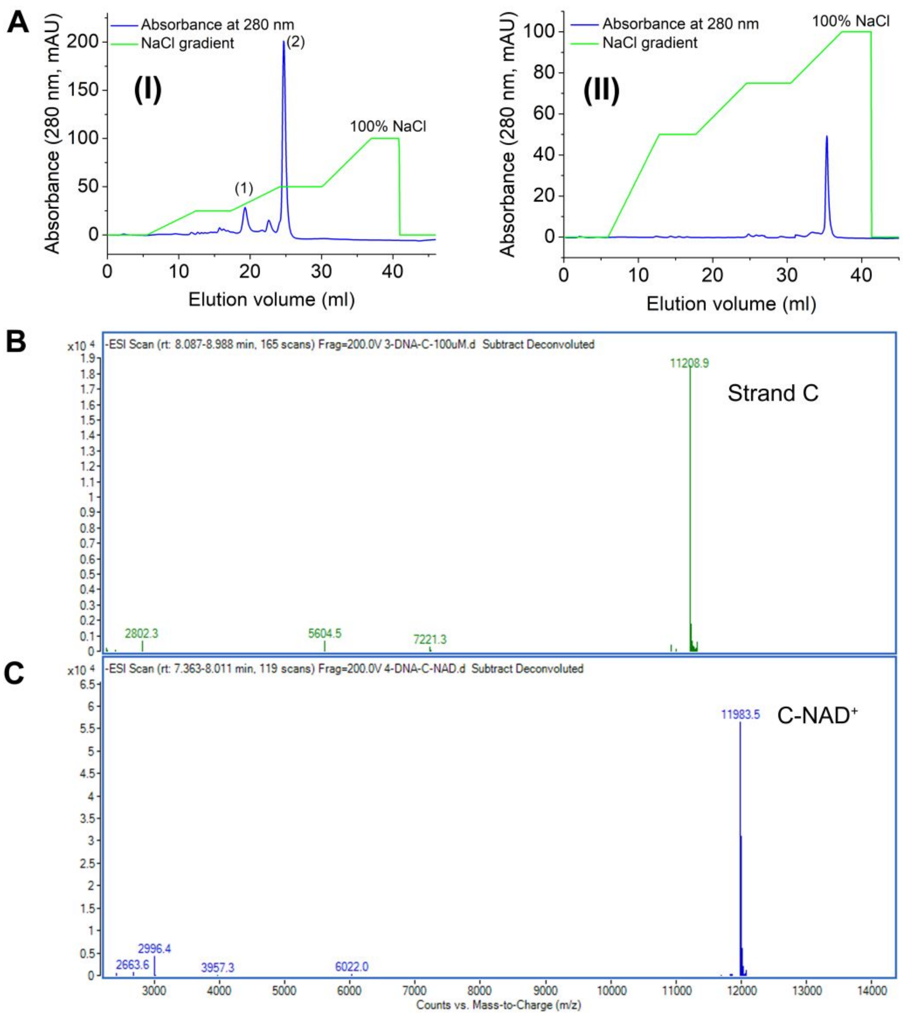

Figure S7. (A) Anion-exchange FPLC to purify DNA-conjugated NAD$^+$. Panel I-The NAD$^+$ modified with different strand C is separated into distinct peaks that are collected in fractions: peak (1)-unmodified strand C; peak (2)-NAD$^+$ labelled with one strand C. Panel II- Anion-exchange FPLC chromatogram output from collected fraction of peak (2) in Panel I. (B) The transformed mass spectrum by deconvolution of strand C. (C)The transformed mass spectrum by deconvolution of conjugate C-NAD$^+$.

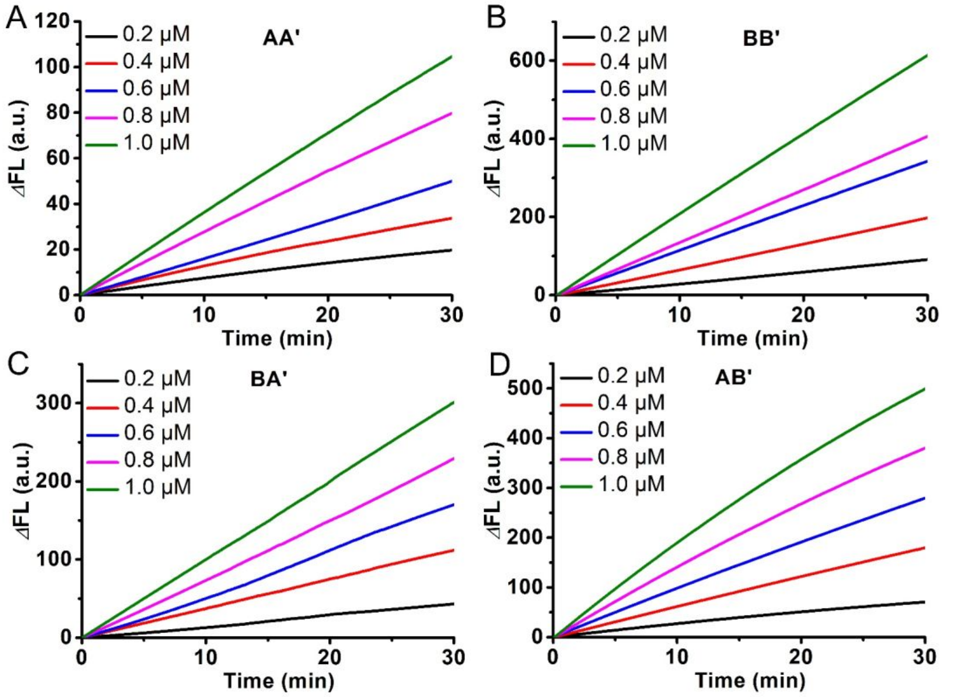

Figure S8. Time-dependent fluorescence changes generated from the cleavage of the fluorophore (F_i)/quencher (Q_i)-modified substrates by the respective DNAzyme reporter units associated with the individual constituents at variable concentrations. It should be noted that the trigger-stabilized constituents reveal similar catalytic activities to the respective constituents without stabilization.

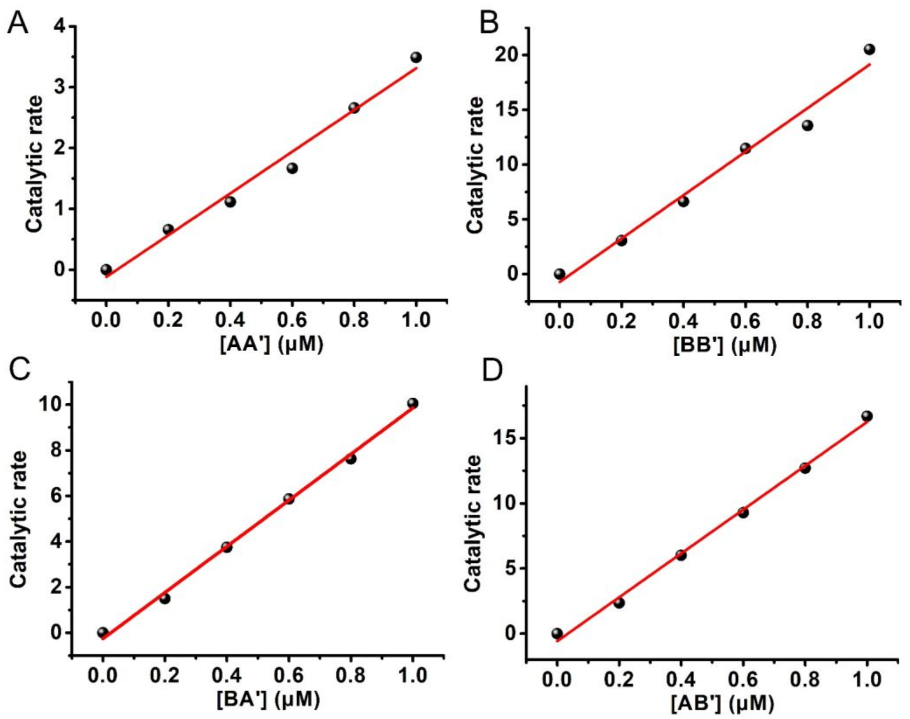

Figure S9. Corresponding calibration curves of the catalytic rates of the constituents as a function of their concentrations, derived from the data shown in Figure S8.

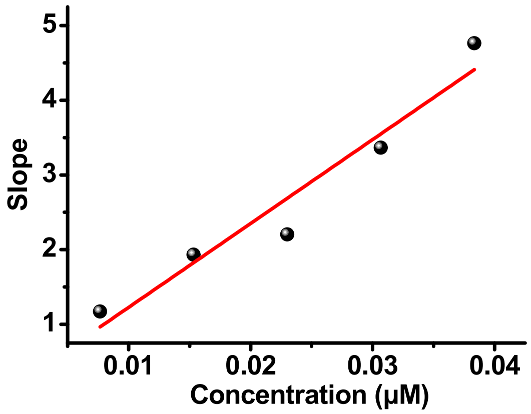

Figure S10. Calibration curve corresponding to the nicking rates of different concentration of nicking enzyme.

Note that, the nicking enzyme of buffer exchange process was achieved by washing with Amicon filter 30 kDa for several times and a calibration curve was used for quantifying the concentration of nicking enzyme. The commercially supplied Nt.BbvCI includes dithiothreitol as stabilizer. Its removal from the reaction mixtures is essential since the modification of the enzymes with the nucleic acid tethers includes an S-S bridge. The dithiothreitol additive degrades the disulfide bridge and, hence, its removal is essential for all the dissipative system.

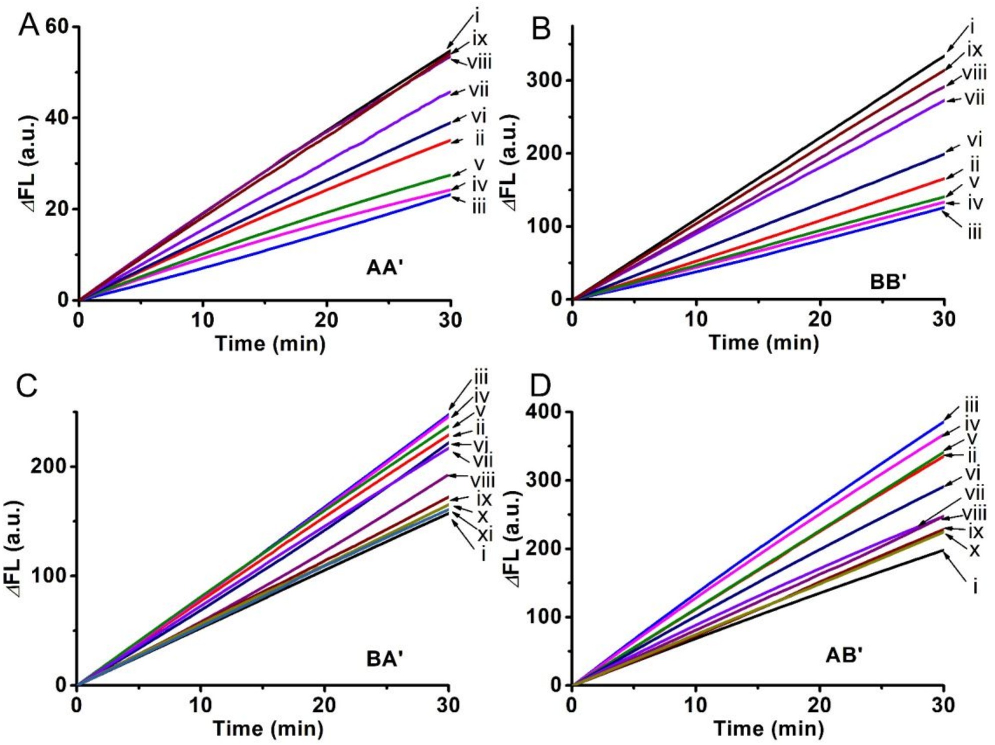

Figure S11. (A) and (B) Time-dependent catalytic activity changes of the DNAzyme reporter units associated with the constituents (AA′ and BB′) at different time intervals during the dissipative transitions of CDN X→Y→X, upon subjecting CDN X that includes the nicking enzyme, 0.0345 μM, to the fuel strand L_1', 6.0 μM: (i) 0 min; (ii) 30 min; (iii) 70 min; (iv) 110 min; (v) 150 min; (vi) 190 min; (vii) 270 min; (viii) 390 min; (ix) 600 min. (C) Time-dependent catalytic activity changes of the DNAzyme reporter units associated with the constituents (BA′) at different time intervals during the dissipative transitions of CDN X→Y→X, upon subjecting CDN X that includes the nicking enzyme, 0.0345 μM, to the fuel strand L_1', 6.0 μM: (i) 0 min; (ii) 30 min; (iii) 70 min; (iv) 110 min; (v) 150 min; (vi) 190 min; (vii) 230 min; (viii) 270 min; (ix) 310 min; (x) 390 min; (xi) 600 min. (D) Time-dependent catalytic activity changes of the DNAzyme reporter units associated with the constituents (AB′) at different time intervals during the dissipative transitions of CDN X→Y→X, upon subjecting CDN X that includes the nicking enzyme, 0.0345 μM, to the fuel strand L_1', 6.0 μM: (i) 0 min; (ii) 30 min; (iii) 70 min; (iv) 150 min; (v) 190 min; (vi) 230 min; (vii) 270 min; (viii) 310 min; (ix) 390 min; (x) 600 min.

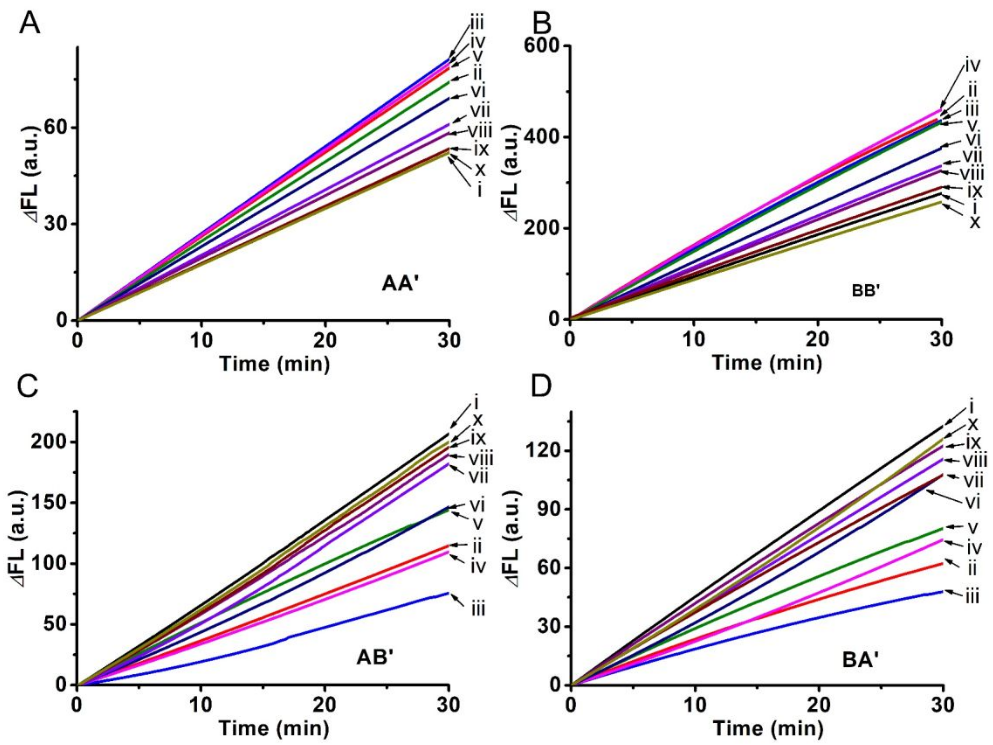

Figure S12. Time-dependent catalytic activity changes of the DNAzyme reporter units associated with the constituents (AA′, BB′, AB′, and BA′) at different time intervals during the dissipative transitions of CDN X→Z→X, upon subjecting CDN X that includes the nicking enzyme, 0.0345 μM, to the fuel strand L_2', 6.0 μM: (i) 0 min; (ii) 30 min; (iii) 70 min; (iv) 110 min; (v) 150 min; (vi) 190 min; (vii) 230 min; (viii) 270 min; (ix) 390 min; (x) 600 min.

Probing the dynamic transient reconfiguration of the transient CDNs by DNAzyme reporter units analysing the withdrawn samples at shorter time intervals (10 minutes nistead of 30 minutes)

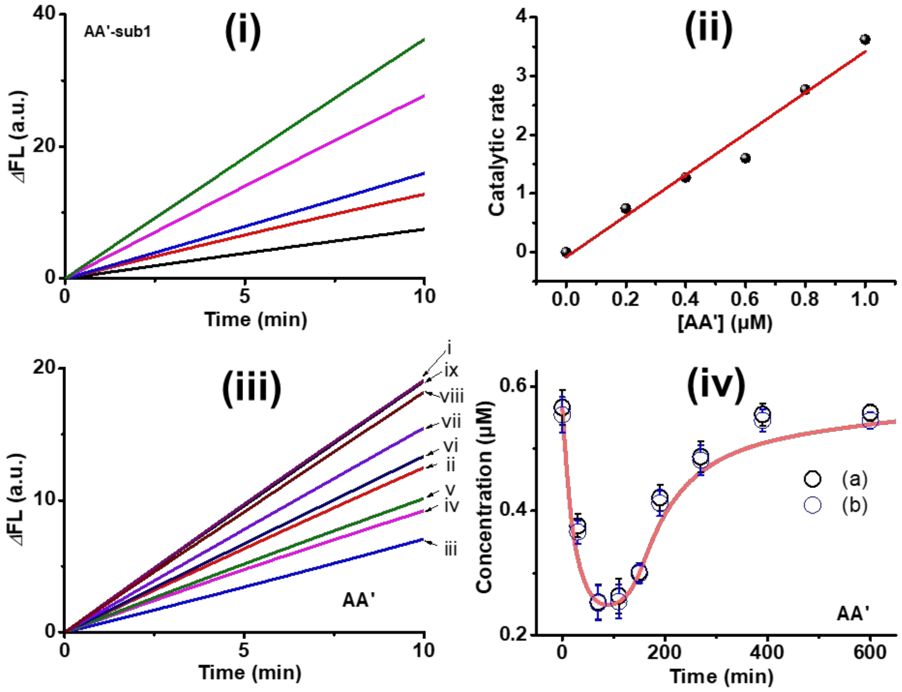

Figure S13. (i) Time-dependent fluorescence changes generated from the cleavage of the fluorophore (F_i)/quencher (Q_i)-modified substrates by the respective DNAzyme reporter units associated with the individual constituents at variable concentrations (10 min time interval). (ii) Calibration curves of the catalytic rates of the constituents as a function of their concentrations, derived from the data shown in (i). (iii) Time-dependent catalytic activity changes of the DNAzyme reporter units associated with the constituents (AA′) at different time intervals during the dissipative transitions of CDN X→Y→X, upon subjecting CDN X that includes the nicking enzyme, 0.0345 µM, to the fuel strand L_1', 6.0 µM. (iv) Temporal concentration changes of the constituents upon the L_1'-triggered reconfiguration of CDN X to CDN Y and its transient recovery to CDN X: (a)-original dotted curves; (b)-dotted curves derived from the calibration curve in (ii).

The samples withdrawn from the dynamically transient operating CDNs were analyzed by the DNAzyme reporter units using the cleavage rate of the DNAzyme substrate on a timescale of 30 minutes (and using the respective rates to extract a calibration curve) to evaluate the temporal concentrations of the constituents. We argued that on a timescale of 8 hours (duration of the transient), no significant concentration changes of the constituents occur on a timescale of 30 minutes.

One of the reviewers questioned the possibility that concentration changes of the constituents in the withdrawn samples may occur on the timescale of 30 minutes and might affect the accuracy of the results.

Accordingly, we shortened the DNAzyme probed analysis time of the constituents in the withdrawn samples from 30 minutes to a timescale of 10 minutes. The results are

presented in Figure S13. We applied a calibration curve relating the DNAzyme reporter cleavage rates to the concentration of the constituent upon probing the reaction rate for 10 minutes, Panel II. This calibration curve was applied to analyze the temporal concentration of the CDN constituents, AA′, in the withdrawn samples within a timescale of 10 minutes. The temporal fluorescence changes and concentrations of AA′, probed within the timescale of 10 minutes are presented in Panel (III) and Panel (IV). The results are basically the same as the results probed a timescale of 30 minutes.

These results imply the temporal concentrations of the constituents probed within the timescale of 10-30 minutes are indistinguishable and possible concentration changes of the CDN constituents at 30 minutes of analysis can be neglected.

Computational simulation of the time-dependent concentration changes of the constituents in the dynamic CDNs.

As an example, to computationally simulate the time-dependent concentration changes of the constituents in CDN X upon reconfiguration of CDN X to CDN Y and back, we formulated a kinetic model that comprises the stepwise reactions associated with the time-dependent concentration changes of the constituents of CDN X upon the L_1'-triggered transition to CDN Y and the nickase guided recovery of CDN Y to CDN X. The sub-reactions are summarized in eq. (1) to eq. (9), where eq. (1) presents the equilibrium reaction between the constituents existing in the two CDNs, eq. (2) and eq. (3) presents the equilibrium process between L_1/T_1 and L_2/T_2, and their separated components (These equations control the equilibrated CDN X). Equation (4) to (9) represent the effect of L_1' on the equilibrated constituents of CDN X and eventually minute interactions of system agents (such as T_2 and AA′) on the overall equilibrated CDN X.

The rate equation corresponding to the respective sub-reactions were defined as equations (10-29). These rate equations were coded in the Matlab R2019b software while providing k_i/k_{-i} values of the set of rate equations, together with the initial concentrations of the constituents in the system at t = 0 min (the k_i/k_{-i} values were based on literature values of related duplexes and provide approximate inputs), resulting in the first dynamic curves corresponding to the temporal concentrations of the constituents. As the experimental temporal concentrations of the constituents are read as a data matrix, the nonlinear least-squares solver (Lsqcurvefit) embedded in Matlab software compares the first round of simulated data experimental data and initiates an optimization iteration under the optimization limits: ′StepTolerance′,1e-90, ′FunctionTolerance′,1e-15, ′OptimalityTolerance′, 1e-20, ′MaxFunctionEvaluation′, 10000). This simulation procedure is processed till a satisfactory fit between the simulated results and experimental results is obtained. Usually, the maximum number of 500 stimulated iterations yield a satisfactory fit between the computationally simulated results and experimental data leading to a set of rate-constants that are supposed to follow the kinetic model. To support the computationally simulated results, it is well desirable to compare one (or more) experimentally validated rate constant to the simulated values. Alternatively, to avoid a possible meaningless set of computationally derived rate constants, the significance of the computational results may be supported by predicting the temporal concentrations of the constituents at different auxiliary conditions and validation of the predicted values by experiments.

Kinetic equations of L_1'-triggered transient transition of CDN X→Y→X shown in Figure 1A:

$$(1) AA' + BB' \underset{k_{-1}}{\overset{k_1}{\rightleftarrows}} AB' + BA'$$

$$(2) L_1 + T_1 \underset{k_{-2}}{\overset{k_2}{\rightleftarrows}} L_1T_1$$

$$(3) T_2 + L_2 \underset{k_{-3}}{\overset{k_3}{\rightleftarrows}} T_2L_2$$

$$(4) T_1L_1 + L_1' \underset{k_{-4}}{\overset{k_4}{\rightleftarrows}} L_1L_1' + T_1$$

$$(5) T_1 + BA' \underset{k_{-5}}{\overset{k_5}{\rightleftarrows}} T_1BA'$$

$$(6) T_2 + AA' \underset{k_{-6}}{\overset{k_6}{\rightleftarrows}} T_2AA'$$

$$(7) E + L_1L_1' \underset{k_{-7}}{\overset{k_7}{\rightleftarrows}} EL_1L_1'$$

$$(8) EL_1L_1' \xrightarrow{k_8} E + L_1L_{1-1}'L_{1-2}'$$

$$(9) L_1L_{1-1}'L_{1-2}' \underset{k_{-9}}{\overset{k_9}{\rightleftarrows}} L_1 + L_{1-1}' + L_{1-2}'$$

Derivatives equations (10-29):

$$(10) \quad \frac{dAA'}{dt} = K_{-1}[AB'][BA'] - K_1[AA'][BB'] + K_{-6}[T_2AA'] - K_6[T_2][AA']$$

$$(11) \quad \frac{dBB'}{dt} = K_{-1}[AB'][BA'] - K_1[AA'][BB']$$

$$(12) \quad \frac{dAB'}{dt} = K_1[AA'][BB'] - K_{-1}[AB'][BA']$$

$$(13) \quad \frac{dBA'}{dt} = K_1[AA'][BB'] - K_{-1}[AB'][BA'] + K_{-5}[T_1BA'] - K_5[T_1][BA']$$

$$(14) \quad \frac{dT_1}{dt} = K_{-2}[T_1L_1] - K_2[T_1][L_1] + K_4[T_1L_1][L_1'] - K_{-4}[L_1L_1'][T_1] + K_{-5}[T_1BA'] - K_5[T_1][BA']$$

$$(15) \quad \frac{dL_1}{dt} = K_{-2}[T_1L_1] - K_2[T_1][L_1] + K_9[L_1L_{1-1}'L_{1-2}'] - K_{-9}[L_1][L_{1-1}'][L_{1-2}']$$

$$(16) \quad \frac{dT_1L_1}{dt} = K_2[T_1][L_1] - K_{-2}[T_1L_1] + K_{-4}[L_1L_1'][T_1] - K_4[T_1L_1][L_1']$$

$$(17) \quad \frac{dT_2}{dt} = K_{-3}[T_2L_2] - K_3[T_2][L_2] + K_{-6}[T_2AA'] - K_6[T_2][AA']$$

$$(18) \quad \frac{dL_2}{dt} = K_{-3}[T_2L_2] - K_3[T_2][L_2]$$

$$(19) \quad \frac{dT_2L_2}{dt} = K_3[T_2][L_2] - K_{-3}[T_2L_2]$$

$$(20) \quad \frac{dL_1'}{dt} = K_{-4}[L_1L_1'][T_1] - K_4[T_1L_1][L_1']$$

$$(21) \quad \frac{dL_1L_1'}{dt} = K_4[T_1L_1][L_1'] - K_{-4}[L_1L_1'][T_1] + K_{-7}[EL_1L_1'] - K_7[E][L_1L_1']$$

$$(22) \quad \frac{dTBA'}{dt} = K_5[T_1][BA'] - K_{-5}[T_1BA']$$

$$(23) \quad \frac{dTAA'}{dt} = K_6[T_2][AA'] - K_{-6}[T_2AA']$$

$$(24) \quad \frac{dE}{dt} = K_{-7}[EL_1L_1'] - K_7[E][L_1L_1'] + K_8[EL_1L_1']$$

$$(25) \quad \frac{dEL_1L_1'}{dt} = K_7[E][L_1L_1'] - K_{-7}[EL_1L_1'] - K_8[EL_1L_1']$$

$$(26) \quad \frac{dL_1L_{1-1}'L_{1-2}'}{dt} = K_8[EL_1L_1'] + K_{-9}[L_1][L_{1-1}'][L_{1-2}'] - K_9[L_1L_{1-1}'L_{1-2}']$$

$$(27) \quad \frac{dL_{1-1}'}{dt} = K_9[L_1L_{1-1}'L_{1-2}'] - K_{-9}[L_1][L_{1-1}'][L_{1-2}']$$

$$(28) \quad \frac{dL_{1-2}'}{dt} = K_9[L_1L_{1-1}'L_{1-2}'] - K_{-9}[L_1][L_{1-1}'][L_{1-2}']$$

(29)

Figure S14. The kinetic scheme of the reactions associated with the time-dependent concentration changes of each constituent in transient transition of CDN X→Y→X is summarized in the above equations. Knowing the time-dependent concentration changes of each constituent, we computationally simulated the time-dependent concentration changes by using Matlab R2019b.

Kinetic equations of L_2'-triggered transient transition of CDN X→Z→X shown in Figure 1A:

$$(1) AA' + BB' \underset{k_{-1}}{\overset{k_1}{\rightleftarrows}} AB' + BA'$$

$$(2) L_1 + T_1 \underset{k_{-2}}{\overset{k_2}{\rightleftarrows}} L_1T_1$$

$$(3) T_2 + L_2 \underset{k_{-3}}{\overset{k_3}{\rightleftarrows}} T_2L_2$$

$$(4) T_2L_2 + L_2' \underset{k_{-4}}{\overset{k_4}{\rightleftarrows}} L_2L_2' + T_2$$

$$(5) T_1 + BA' \underset{k_{-5}}{\overset{k_5}{\rightleftarrows}} T_1BA'$$

$$(6) T_2 + AA' \underset{k_{-6}}{\overset{k_6}{\rightleftarrows}} T_2AA'$$

$$(7) E + L_2L_2' \underset{k_{-7}}{\overset{k_7}{\rightleftarrows}} EL_2L_2'$$

$$(8) EL_2L_2' \xrightarrow{k_8} E + L_2L_{2-1}'L_{2-2}'$$

$$(9) L_2L_{1-1}'L_{2-2}' \underset{k_{-9}}{\overset{k_9}{\rightleftarrows}} L_2 + L_{2-1}' + L_{2-2}'$$

Derivatives:

$$\frac{dAA'}{dt} = K_{-1}[AB'][BA'] - K_1[AA'][BB'] + K_{-6}[T_2AA'] - K_6[T_2][AA']$$

$$\frac{dBB'}{dt} = K_{-1}[AB'][BA'] - K_1[AA'][BB']$$

$$\frac{dAB'}{dt} = K_1[AA'][BB'] - K_{-1}[AB'][BA']$$

$$\frac{dBA'}{dt} = K_1[AA'][BB'] - K_{-1}[AB'][BA'] + K_{-5}[T_1BA'] - K_5[T_1][BA']$$

$$\frac{dT_1}{dt} = K_{-2}[T_1L_1] - K_2[T_1][L_1] + K_{-5}[T_1BA'] - K_5[T_1][BA']$$

$$\frac{dL_1}{dt} = K_{-2}[T_1L_1] - K_2[T_1][L_1]$$

$$\frac{dT_1L_1}{dt} = K_2[T_1][L_1] - K_{-2}[T_1L_1]$$

$$\frac{dT_2}{dt} = K_{-3}[T_2L_2] - K_3[T_2][L_2] + K_4[T_2L_2][L_2'] - K_{-4}[L_2L_2'][T_2]$$

$$\frac{dL_2}{dt} = K_{-3}[T_2L_2] - K_3[T_2][L_2] + K_9[L_2L_{2-1}'L_{2-2}'] - K_{-9}[L_2][L_{2-1}'][L_{2-2}']$$

$$\frac{dT_2L_2}{dt} = K_3[T_2][L_2] - K_{-3}[T_2L_2] + K_{-4}[L_2L_2'][T_2] - K_4[T_2L_2][L_2']$$

$$\frac{dL_2'}{dt} = K_{-4}[L_2L_2'][T_2] - K_4[T_2L_2][L_2']$$

$$\frac{dL_2L_2'}{dt} = K_4[T_2L_2][L_2'] - K_{-4}[L_2L_2'][T_2] + K_{-7}[EL_2L_2'] - K_7[E][L_2L_2']$$

$$\frac{dTBA'}{dt} = K_5[T_1][BA'] - K_{-5}[T_1BA']$$

$$\frac{dTAA'}{dt} = K_6[T_2][AA'] - K_{-6}[T_2AA']$$

$$\frac{dE}{dt} = K_{-7}[EL_2L_2'] - K_7[E][L_2L_2'] + K_8[EL_2L_2']$$

$$\frac{dEL_2L_2'}{dt} = K_7[E][L_2L_2'] - K_{-7}[EL_2L_2'] - K_8[EL_2L_2']$$

$$\frac{dL_2L_{2-1}'L_{2-2}'}{dt} = K_8[EL_2L_2'] + K_{-9}[L_2][L_{2-1}'][L_{2-2}'] - K_9[L_2L_{2-1}'L_{2-2}']$$

$$\frac{dL_{2-1}'}{dt} = K_9[L_2L_{2-1}'L_{2-2}'] - K_{-9}[L_2][L_{2-1}'][L_{2-2}']$$

$$\frac{dL_{2-2}'}{dt} = K_9[L_2L_{2-1}'L_{2-2}'] - K_{-9}[L_2][L_{2-1}'][L_{2-2}']$$

Figure S15. The kinetic scheme of the reactions associated with the time-dependent concentration changes of each constituent in transient transition of CDN X→Z→X is summarized in the above equations. Knowing the time-dependent concentration changes of each constituent, we computationally simulated the time-dependent concentration changes by using Matlab R2019b.

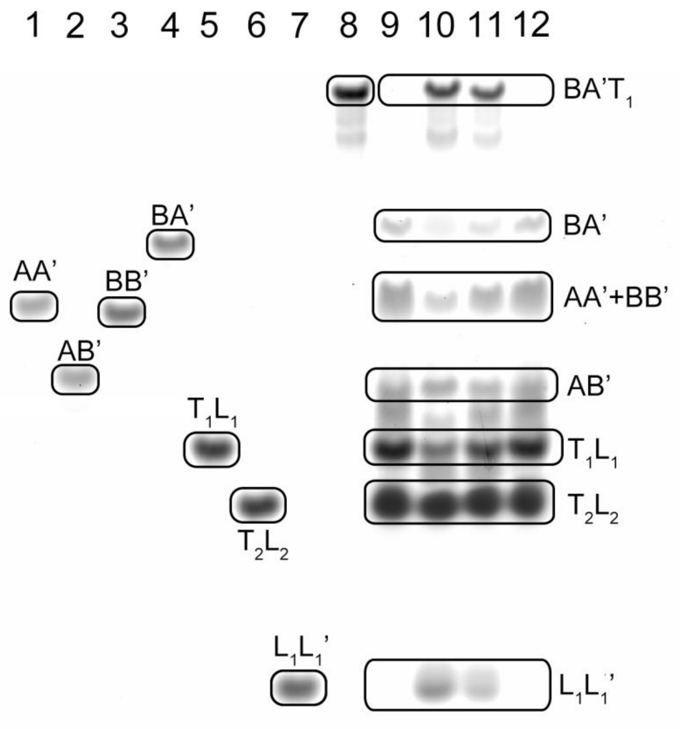

Figure S16. Electrophoretic quantitative separation of the constituents and the accompanying structures at different time intervals during the dissipative transitions of CDN "X" to CDN "Y" and back, upon subjecting CDN "X" that includes the nicking enzyme, 0.0345 µM, to the fuel strand L_1', 6 µM. Lanes 1-8, control bands corresponding to the individual structures: Lane 1-AA′; Lane 2-AB′; Lane 3- BB′; Lane 4-BA′; Lane 5- T_1L_1; Lane 6-T_2L_2; Lane 7- L_1L_1'; Lane 8-BA′T_1. Lane 9-Separated bands of CDN "X". Lane 10-Separated bands associated with the structures upon subjecting CDN "X" to the fuel strand L_1' for 70 min. Lane 11-Separated bands corresponding to the structures upon treatment of CDN "X" with the fuel strand L_1' for 190 min. Lane 12-Separated bands corresponding to the structures upon treatment of CDN "X" with the fuel strand L_1' for 600 min. Comparison of lanes 9-12 drives conclusions: (1) Upon treatment of CDN "X" with fuel trigger of L_1', the band of L_1L_1' is observed with a quantified concentration of 0.6 µM, the band of T_1L_1 change its quantified concentration from 0.98 µM to 0.39 µM, the quantified concentration of constituents AB′ and BA′ increase approximately from 0.5 µM to 0.8 µM while the sum of AA′ and BB′ decrease from 1 µM to 0.43 µM, respectively. This result demonstrates that the trigger L_1' reconfigures the CDN "X" to CDN "Y". (2) Upon mixing the CDN "X" with the fuel strand L_1' for 190 min and 600 min, the intensity of band L_1L_1' is decreased and depleted due to the cleavage of L_1' by the nicking enzyme. In addition, the released L_1 from duplex L_1/L_1' replace the BA′T1 into the duplexes L_1/T_1 and BA′, leading to the gradual recovery of concentration of all constituents in CDN "Y" into CDN "X" (AA′, BB′, AB′, BA′ recover to approximate 0.5 µM, the L_1T_1 recover to approximate 1 µM). This result demonstrates the network is transiently recovered from CDN "Y" into CDN "X".

Table S1. Quantitative assessment of the concentrations of the constituents at different time intervals during the dissipative transitions of CDN "X" to CDN "Y" and back, upon subjecting CDN "X" that includes the nicking enzyme, 0.0345 μM, to L_1', 6 μM: (i) 0 min; (ii) 70 min; (iii) 190 min; (iv) 600 min.

	[AA′] (μM)	[BB′] (μM)	[BA′] (μM)	[AB′] (μM)	L_1T_1 (μM)	L_1L_1' (μM)
i[a]	0.54±0.02	0.55±0.01	0.51±0.03	0.48±0.01	--	--
i[b]	1.01[c]		0.48[d]	0.49	0.98	0
ii[a]	0.25±0.04	0.25±0.03	0.84±0.02	0.82±0.02	--	--
ii[b]	0.43[c]		0.82[d]	0.80	0.39	0.6
iii[a]	0.42±0.03	0.39±0.02	0.75±0.03	0.73±0.03	--	--
iii[b]	0.79[c]		0.71[d]	0.73	0.61	0.42
iv[a]	0.53±0.01	0.54±0.03	0.52±0.01	0.49±0.01	--	--
iv[b]	1.03[c]		0.50[d]	0.48	0.99	0

[a]Data provided by the catalytic DNAzyme reporter units. [b]Data derived from the quantitative gel electrophoresis. [c]Sum of AA′ and BB′ (Bands cannot be evaluated due to the overlap). [d]Sum of BA′ and $BA'T_1$.

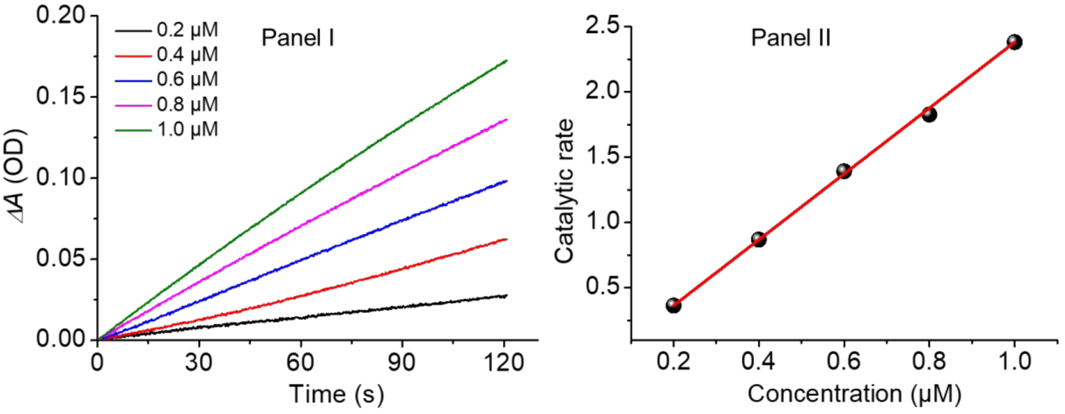

Figure S17. Panel I. Time-dependent absorbance changes of $ABTS^{\bullet-}$ driven by different concentrations of GOx/HRP-BB′ (after subjecting B-GOx, 1 μM, B′-HRP, 1 μM, to different concentrations of complementary strand of B under glucose, 40 μM, $ABTS^{2-}$, 100 μM). Penal II. Calibration curve corresponding to the absorbance difference of different concentrations of GOx/HRP-BB′.

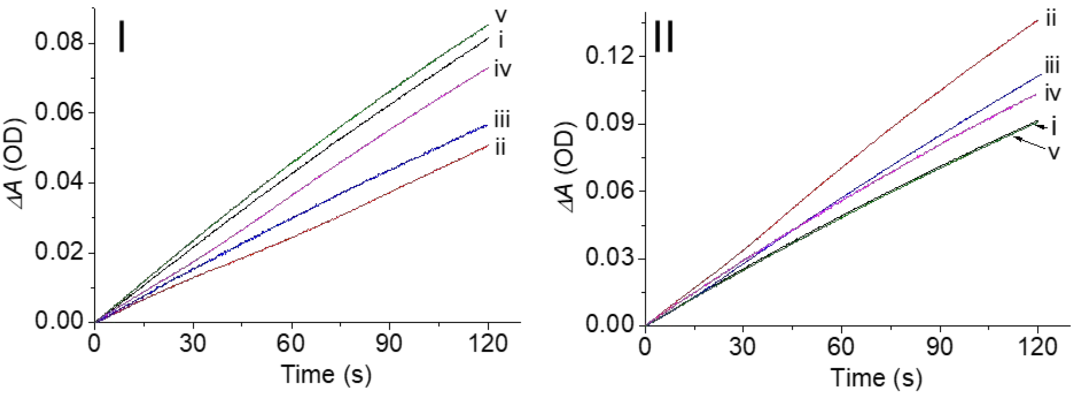

Figure S18. Panel (I)-Time-dependent concentration changes of ABTS$^{•-}$ generated by transient reaction samples of the GOx/HRP cascade upon the L_1' (4 μM)-triggered dynamic reconfiguration of CDN X to CDN Y and back: (i) t = 0, (ii) after 50 min, (ii) after 100 min, (iv) after 200 min, (v) after 300 min. Panel (I)-Time-dependent concentration changes of ABTS$^{•-}$ generated by transient reaction samples of the GOx/HRP cascade upon the L_2' (4 μM)-triggered dynamic reconfiguration of CDN X to CDN Z and back: (i) t = 0, (ii) after 50 min, (ii) after 100 min, (iv) after 200 min, (v) after 300 min.

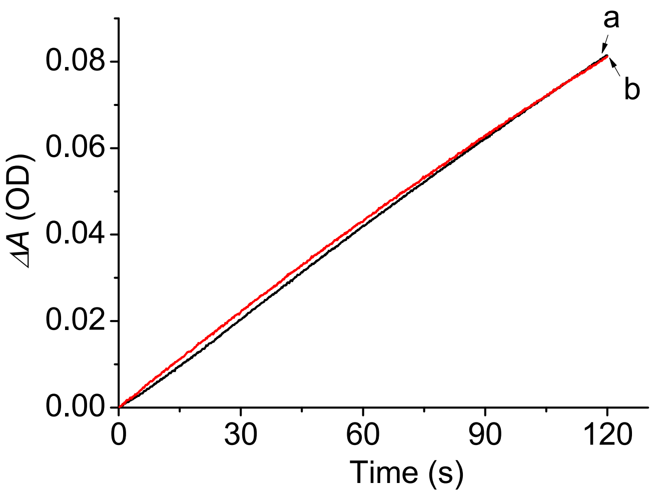

Figure S19. (a) Time-dependent concentration changes of $ABTS^{\bullet-}$ generated by transient reaction samples of the GOx/HRP cascade upon the L_1' (6 μM)-triggered dynamic reconfiguration of CDN X consisting of original L_1/T_1 duplex to CDN Y. (b) Time-dependent concentration changes of $ABTS^{\bullet-}$ generated by transient reaction samples of the GOx/HRP cascade upon the L_1' (6 μM)-triggered dynamic reconfiguration of CDN X that exchanged with a L_1^M/T_1 duplex to CDN Y.

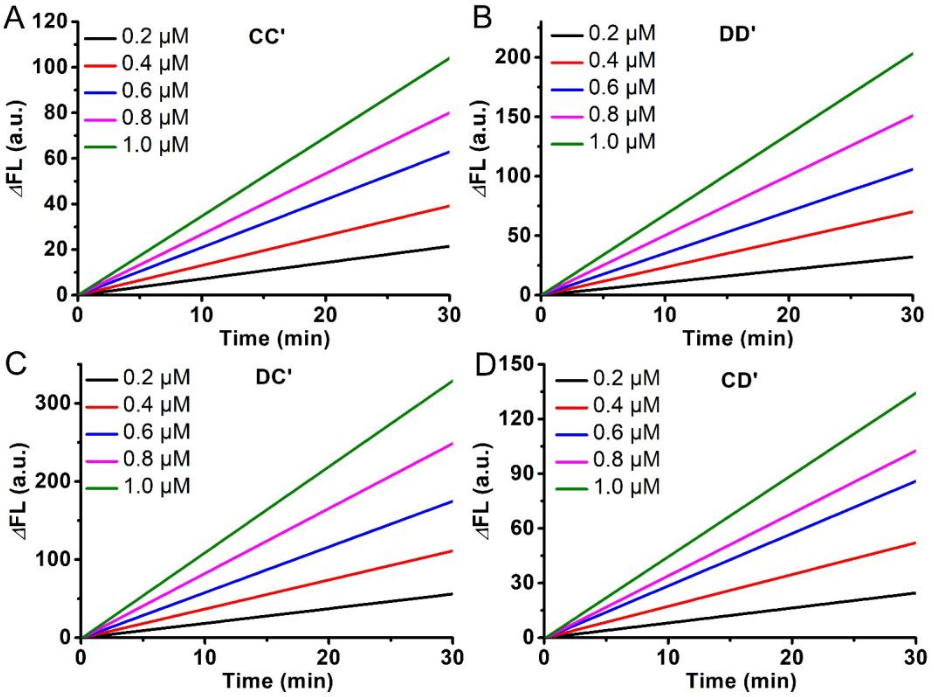

Figure S20. Time-dependent fluorescence changes generated from the cleavage of the fluorophore (F_i)/quencher (Q_i)-modified substrates by the respective DNAzyme reporter units associated with the individual constituents at variable concentrations. It should be noted that the trigger-stabilized constituents reveal similar catalytic activities to the respective constituents without stabilization.

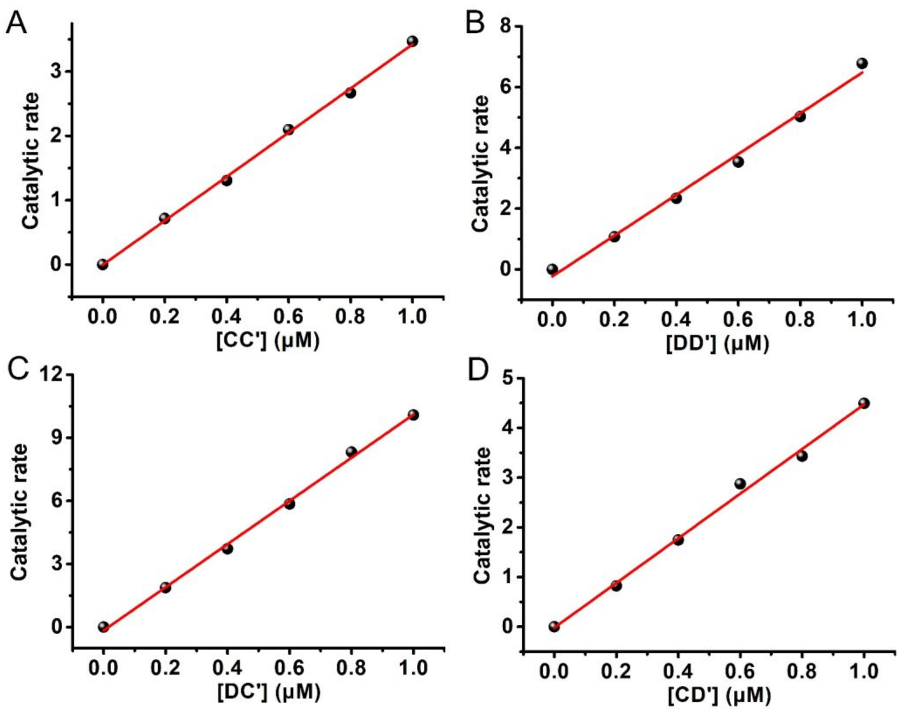

Figure S21. Corresponding calibration curves of the catalytic rates of the constituents as a function of their concentrations, derived from the data shown in Figure S19.

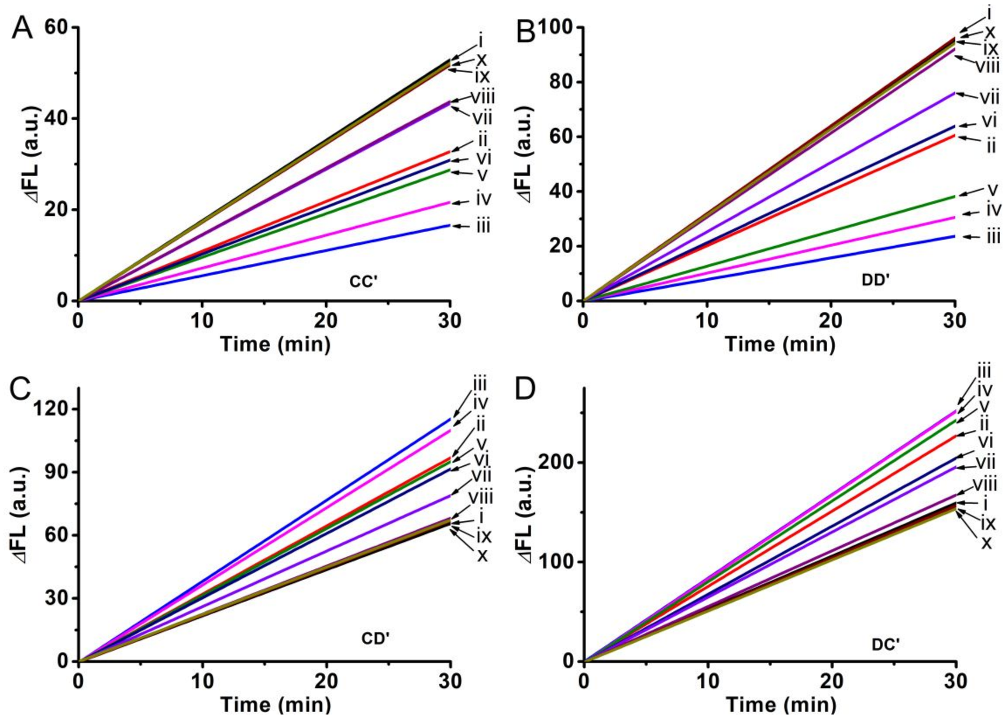

Figure S22. Time-dependent catalytic activity changes of the DNAzyme reporter units associated with the constituents (CC′, DD′, CD′ and DC′) at different time intervals during the dissipative transitions of CDN O→M→O, upon subjecting CDN O that includes the nicking enzyme, 0.0345 μM, to the fuel strand L_1', 6.0 μM: (i) 0 min; (ii) 30 min; (iii) 70 min; (iv) 110 min; (v) 150 min; (vi) 190 min; (vii) 230 min; (viii) 270 min; (ix) 390 min; (x) 600 min.

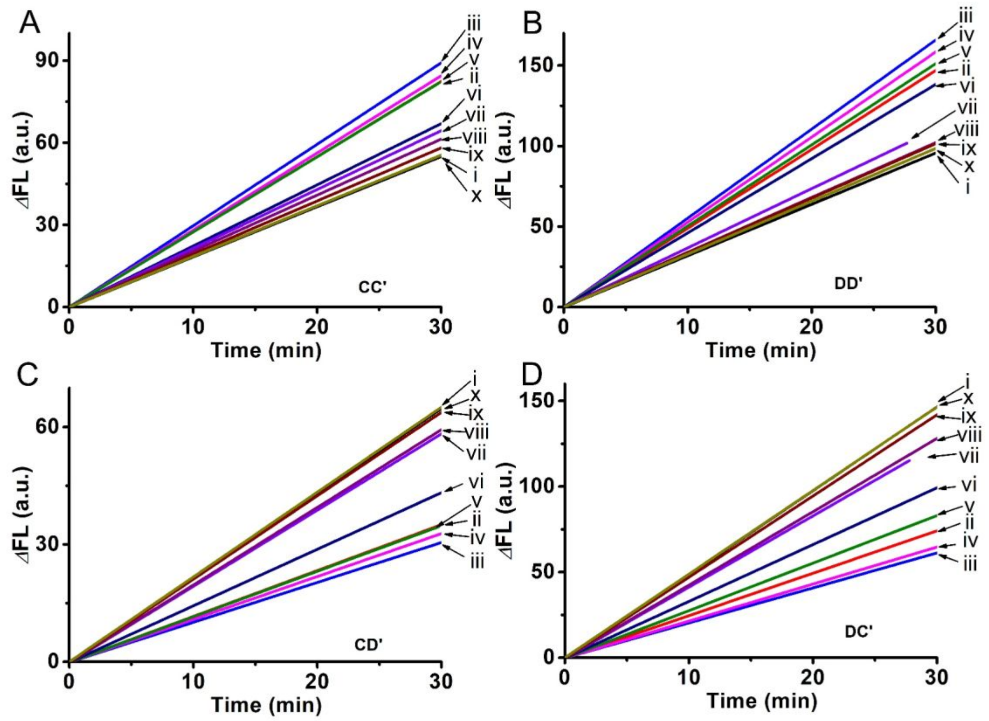

Figure S23. Time-dependent catalytic activity changes of the DNAzyme reporter units associated with the constituents (CC′, DD′, CD′ and DC′) at different time intervals during the dissipative transitions of CDN O→P→O, upon subjecting CDN O that includes the nicking enzyme, 0.0345 μM, to the fuel strand L_2', 6.0 μM: (i) 0 min; (ii) 30 min; (iii) 70 min; (iv) 110 min; (v) 150 min; (vi) 190 min; (vii) 230 min; (viii) 270 min; (ix) 390 min; (x) 600 min.

Kinetic equations of L_1'-triggered transient transition of CDN O→M→O shown in Figure 2A:

$$(1) CC' + DD' \underset{k_{-1}}{\overset{k_1}{\rightleftharpoons}} CD' + DC'$$

$$(2) L_1 + T_1 \underset{k_{-2}}{\overset{k_2}{\rightleftharpoons}} L_1T_1$$

$$(3) T_2 + L_2 \underset{k_{-3}}{\overset{k_3}{\rightleftharpoons}} T_2L_2$$

$$(4) T_1L_1 + L_1' \underset{k_{-4}}{\overset{k_4}{\rightleftharpoons}} L_1L_1' + T_1$$

$$(5) T_1 + DC' \underset{k_{-5}}{\overset{k_5}{\rightleftharpoons}} T_1DC'$$

$$(6) T_2 + CC' \underset{k_{-6}}{\overset{k_6}{\rightleftharpoons}} T_2CC'$$

$$(7) E + L_1L_1' \underset{k_{-7}}{\overset{k_7}{\rightleftharpoons}} EL_1L_1'$$

$$(8) EL_1L_1' \xrightarrow{k_8} E + L_1L_{1-1}'L_{1-2}'$$

$$(9) L_1L_{1-1}'L_{1-2}' \underset{k_{-9}}{\overset{k_9}{\rightleftharpoons}} L_1 + L_{1-1}' + L_{1-2}'$$

Derivatives:

$$\frac{dCC'}{dt} = K_{-1}[CD'][DC'] - K_1[CC'][DD'] + K_{-6}[T_2CC'] - K_6[T_2][CC']$$

$$\frac{dDD'}{dt} = K_{-1}[CD'][DC'] - K_1[CC'][DD']$$

$$\frac{dCD'}{dt} = K_1[CC'][DD'] - K_{-1}[CD'][DC']$$

$$\frac{dDC'}{dt} = K_1[CC'][DD'] - K_{-1}[CD'][DC'] + K_{-5}[T_1DC'] - K_5[T_1][DC']$$

$$\frac{dT_1}{dt} = K_{-2}[T_1L_1] - K_2[T_1][L_1] + K_4[T_1L_1][L_1'] - K_{-4}[L_1L_1'][T_1] + K_{-5}[T_1DC'] - K_5[T_1][DC']$$

$$\frac{dL_1}{dt} = K_{-2}[T_1L_1] - K_2[T_1][L_1] + K_9[L_1L_{1-1}'L_{1-2}'] - K_{-9}[L_1][L_{1-1}'][L_{1-2}']$$

$$\frac{dT_1L_1}{dt} = K_2[T_1][L_1] - K_{-2}[T_1L_1] + K_{-4}[L_1L_1'][T_1] - K_4[T_1L_1][L_1']$$

$$\frac{dT_2}{dt} = K_{-3}[T_2L_2] - K_3[T_2][L_2] + K_{-6}[T_2CC'] - K_6[T_2][CC']$$

$$\frac{dL_2}{dt} = K_{-3}[T_2L_2] - K_3[T_2][L_2]$$

$$\frac{dT_2L_2}{dt} = K_3[T_2][L_2] - K_{-3}[T_2L_2]$$

$$\frac{dL_1'}{dt} = K_{-4}[L_1L_1'][T_1] - K_4[T_1L_1][L_1']$$

$$\frac{dL_1L_1'}{dt} = K_4[T_1L_1][L_1'] - K_{-4}[L_1L_1'][T_1] + K_{-7}[EL_1L_1'] - K_7[E][L_1L_1']$$

$$\frac{dTDC'}{dt} = K_5[T_1][DC'] - K_{-5}[T_1DC']$$

$$\frac{dTCC'}{dt} = K_6[T_2][CC'] - K_{-6}[T_2CC']$$

$$\frac{dE}{dt} = K_{-7}[EL_1L_1'] - K_7[E][L_1L_1'] + K_8[EL_1L_1']$$

$$\frac{dEL_1L_1'}{dt} = K_7[E][L_1L_1'] - K_{-7}[EL_1L_1'] - K_8[EL_1L_1']$$

$$\frac{dL_1L_{1-1}'L_{1-2}'}{dt} = K_8[EL_1L_1'] + K_{-9}[L_1][L_{1-1}'][L_{1-2}'] - K_9[L_1L_{1-1}'L_{1-2}']$$

$$\frac{dL_{1-1}'}{dt} = K_9[L_1L_{1-1}'L_{1-2}'] - K_{-9}[L_1][L_{1-1}'][L_{1-2}']$$

$$\frac{dL_{1-2}'}{dt} = K_9[L_1L_{1-1}'L_{1-2}'] - K_{-9}[L_1][L_{1-1}'][L_{1-2}']$$

Figure S24. The kinetic scheme of the reactions associated with the time-dependent concentration changes of each constituent in transient transition of CDN O→M→O is summarized in the above equations. Knowing the time-dependent concentration changes of each constituent, we computationally simulated the time-dependent concentration changes by using Matlab R2019b.

Kinetic equations of L_2'-triggered transient transition of CDN O→P→O shown in Figure 2A:

$(1) CC' + DD' \underset{k_{-1}}{\overset{k_1}{\rightleftarrows}} CD' + DC'$

$(2) L_1 + T_1 \underset{k_{-2}}{\overset{k_2}{\rightleftarrows}} L_1T_1$

$(3) T_2 + L_2 \underset{k_{-3}}{\overset{k_3}{\rightleftarrows}} T_2L_2$

$(4) T_2L_2 + L_2' \underset{k_{-4}}{\overset{k_4}{\rightleftarrows}} L_2L_2' + T_2$

$(5) T_1 + DC' \underset{k_{-5}}{\overset{k_5}{\rightleftarrows}} T_1DC'$

$(6) T_2 + CC' \underset{k_{-6}}{\overset{k_6}{\rightleftarrows}} T_2CC'$

$(7) E + L_2L_2' \underset{k_{-7}}{\overset{k_7}{\rightleftarrows}} EL_2L_2'$

$(8) EL_2L_2' \xrightarrow{k_8} E + L_2L_{2-1}'L_{2-2}'$

$(9) L_2L_{1-1}'L_{2-2}' \underset{k_{-9}}{\overset{k_9}{\rightleftarrows}} L_2 + L_{2-1}' + L_{2-2}'$

Derivatives:

$$\frac{dCC'}{dt} = K_{-1}[CD'][DC'] - K_1[CC'][DD'] + K_{-6}[T_2CC'] - K_6[T_2][CC']$$

$$\frac{dDD'}{dt} = K_{-1}[CD'][DC'] - K_1[CC'][DD']$$

$$\frac{dCD'}{dt} = K_1[CC'][DD'] - K_{-1}[CD'][DC']$$

$$\frac{dDC'}{dt} = K_1[CC'][DD'] - K_{-1}[CD'][DC'] + K_{-5}[T_1DC'] - K_5[T_1][DC']$$

$$\frac{dT_1}{dt} = K_{-2}[T_1L_1] - K_2[T_1][L_1] + K_{-5}[T_1DC'] - K_5[T_1][DC']$$

$$\frac{dL_1}{dt} = K_{-2}[T_1L_1] - K_2[T_1][L_1]$$

$$\frac{dT_1L_1}{dt} = K_2[T_1][L_1] - K_{-2}[T_1L_1]$$

$$\frac{dT_2}{dt} = K_{-3}[T_2L_2] - K_3[T_2][L_2] + K_4[T_2L_2][L_2'] - K_{-4}[L_2L_2'][T_2]$$

$$\frac{dL_2}{dt} = K_{-3}[T_2L_2] - K_3[T_2][L_2] + K_9[L_2L_{2-1}'L_{2-2}'] - K_{-9}[L_2][L_{2-1}'][L_{2-2}']$$

$$\frac{dT_2L_2}{dt} = K_3[T_2][L_2] - K_{-3}[T_2L_2] + K_{-4}[L_2L_2'][T_2] - K_4[T_2L_2][L_2']$$

$$\frac{dL_2'}{dt} = K_{-4}[L_2L_2'][T_2] - K_4[T_2L_2][L_2']$$

$$\frac{dL_2L_2'}{dt} = K_4[T_2L_2][L_2'] - K_{-4}[L_2L_2'][T_2] + K_{-7}[EL_2L_2'] - K_7[E][L_2L_2']$$

$$\frac{dTDC'}{dt} = K_5[T_1][DC'] - K_{-5}[T_1DC']$$

$$\frac{dTCC'}{dt} = K_6[T_2][CC'] - K_{-6}[T_2DD']$$

$$\frac{dE}{dt} = K_{-7}[EL_2L_2'] - K_7[E][L_2L_2'] + K_8[EL_2L_2']$$

$$\frac{dEL_2L_2'}{dt} = K_7[E][L_2L_2'] - K_{-7}[EL_2L_2'] - K_8[EL_2L_2']$$

$$\frac{dL_2L_{2-1}'L_{2-2}'}{dt} = K_8[EL_2L_2'] + K_{-9}[L_2][L_{2-1}'][L_{2-2}'] - K_9[L_2L_{2-1}'L_{2-2}']$$

$$\frac{dL_{2-1}'}{dt} = K_9[L_2L_{2-1}'L_{2-2}'] - K_{-9}[L_2][L_{2-1}'][L_{2-2}']$$

$$\frac{dL_{2-2}'}{dt} = K_9[L_2L_{2-1}'L_{2-2}'] - K_{-9}[L_2][L_{2-1}'][L_{2-2}']$$

Figure S25. The kinetic scheme of the reactions associated with the time-dependent concentration changes of each constituent in transient transition of CDN O→P→O is summarized in the above equations. Knowing the time-dependent concentration

changes of each constituent, we computationally simulated the time-dependent concentration changes by using Matlab R2019b.

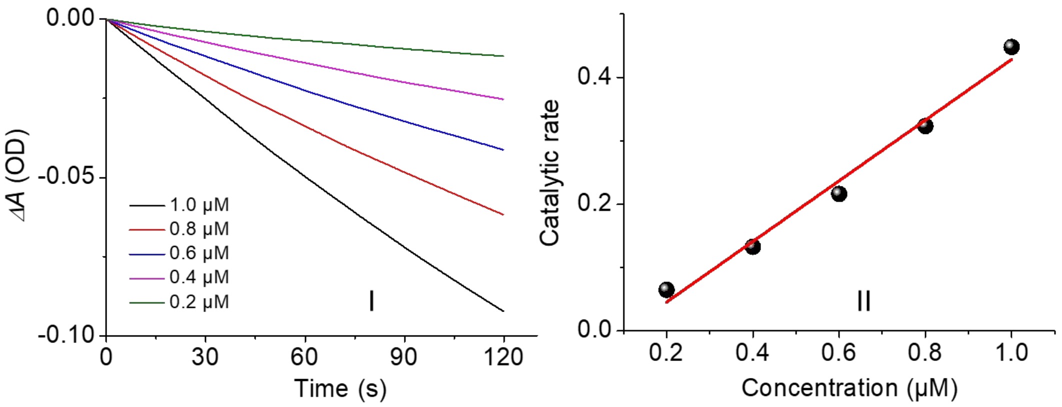

Figure S26. Panel I-Time-dependent absorbance changes of MB^+ driven by different concentrations of LDH/NAD^+-CD′ (after subjecting D′-LDH, 1 μM, C-NAD^+, 1 μM, to different concentrations of complementary strand of C under 1 mM lattic acid, 1 mM hydrazine, and 50 μM MB^+). Penal II-Calibration curve corresponding to the absorbance difference of different concentrations of LDH/NAD^+-CD′.

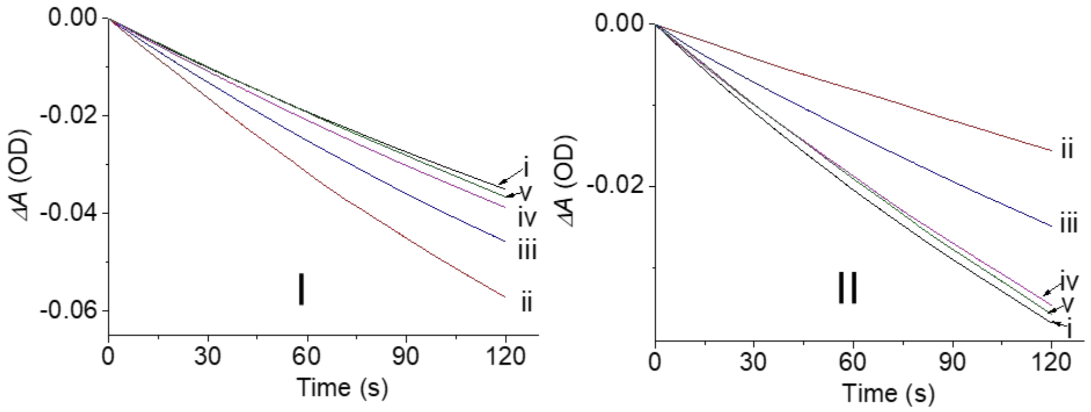

Figure S27. Panel I-Time-dependent concentration changes of MB^+ generated by transient reaction samples of the LDH/NAD^+ cascade upon the L_1' (4 μM)-triggered dynamic reconfiguration of CDN O to CDN M and back: (i) t = 0, (ii) after 50 min, (ii) after 100 min, (iv) after 200 min, (v) after 300 min. Panel II-Time-dependent concentration changes of MB^+ generated by transient reaction samples of the LDH/NAD^+ cascade upon the L_2' (4 μM)-triggered dynamic reconfiguration of CDN O to CDN P and back: (i) t = 0, (ii) after 50 min, (ii) after 100 min, (iv) after 200 min, (v) after 300 min.

Kinetic equations of L_1'-triggered transient transition of CDN H→L→H shown in Figure 3A:

$$(1) AA' + BB' \underset{k_{-1}}{\overset{k_1}{\rightleftharpoons}} AB' + BA'$$

$$(2) CC' + DD' \underset{k_{-2}}{\overset{k_2}{\rightleftharpoons}} CD' + DC'$$

$$(3) L_1 + T_1 \underset{k_{-3}}{\overset{k_3}{\rightleftharpoons}} L_1T_1$$

$$(4) T_2 + L_2 \underset{k_{-4}}{\overset{k_4}{\rightleftharpoons}} T_2L_2$$

$$(5) T_1L_1 + L_1' \underset{k_{-5}}{\overset{k_5}{\rightleftharpoons}} L_1L_1' + T_1$$

$$(6) T_1 + BA' \underset{k_{-6}}{\overset{k_6}{\rightleftharpoons}} T_1BA'$$

$$(7) T_2 + AA' \underset{k_{-7}}{\overset{k_7}{\rightleftharpoons}} T_2AA'$$

$$(8) T_1 + DC' \underset{k_{-8}}{\overset{k_8}{\rightleftharpoons}} T_1DC'$$

$$(9) T_2 + CC' \underset{k_{-9}}{\overset{k_9}{\rightleftharpoons}} T_2CC'$$

$$(10) E + L_1L_1' \underset{k_{-10}}{\overset{k_{10}}{\rightleftharpoons}} EL_1L_1'$$

$$(11) EL_1L_1' \xrightarrow{k_{11}} E + L_1L_{1-1}'L_{1-2}'$$

$$(12) L_1L_{1-1}'L_{1-2}' \underset{k_{-12}}{\overset{k_{12}}{\rightleftharpoons}} L_1 + L_{1-1}' + L_{1-2}'$$

Derivatives:

$$\frac{dAA'}{dt}=K_{-1}[AB'][BA']-K_1[AA'][BB']+K_{-7}[T_2AA']-K_7[T_2][AA']$$

$$\frac{dBB'}{dt}=K_{-1}[AB'][BA']-K_1[AA'][BB']$$

$$\frac{dAB'}{dt}=K_1[AA'][BB']-K_{-1}[AB'][BA']$$

$$\frac{dBA'}{dt}=K_1[AA'][BB']-K_{-1}[AB'][BA']+K_{-6}[T_1BA']-K_6[T_1][BA']$$

$$\frac{dCC'}{dt}=K_{-2}[CD'][DC']-K_2[CC'][DD']+K_{-9}[T_2CC']-K_9[T_2][CC']$$

$$\frac{dDD'}{dt}=K_{-2}[CD'][DC']-K_2[CC'][DD']$$

$$\frac{dCD'}{dt}=K_2[CC'][DD']-K_{-2}[CD'][DC']$$

$$\frac{dDC'}{dt}=K_2[CC'][DD']-K_{-2}[CD'][DC']+K_{-8}[T_1DC']-K_8[T_1][DC']$$

$$\frac{dT_1}{dt}=K_{-3}[T_1L_1]-K_3[T_1][L_1]+K_5[T_1L_1][L_1']-K_{-5}[L_1L_1'][T_1]+K_{-6}[T_1BA']-K_6[T_1][BA']+K_{-8}[T_1DC']-K_8[T_1][DC']$$

$$\frac{dL_1}{dt}=K_{-3}[T_1L_1]-K_3[T_1][L_1]+K_{12}[L_1L_{1-1}'L_{1-2}']-K_{-12}[L_1][L_{1-1}'][L_{1-2}']$$

$$\frac{dT_1L_1}{dt}=K_3[T_1][L_1]-K_{-3}[T_1L_1]+K_{-5}[L_1L_1'][T_1]-K_5[T_1L_1][L_1']$$

$$\frac{dT_2}{dt}=K_{-4}[T_2L_2]-K_4[T_2][L_2]+K_{-7}[T_2AA']-K_7[T_2][AA']+K_{-9}[T_2CC']-K_9[T_2][CC']$$

$$\frac{dL_2}{dt}=K_{-4}[T_2L_2]-K_4[T_2][L_2]$$

$$\frac{dT_2L_2}{dt}=K_4[T_2][L_2]-K_{-4}[T_2L_2]$$

$$\frac{dL_1'}{dt}=K_{-5}[L_1L_1'][T_1]-K_5[T_1L_1][L_1']$$

$$\frac{dL_1L_1'}{dt}=K_5[T_1L_1][L_1']-K_{-5}[L_1L_1'][T_1]+K_{-10}[EL_1L_1']-K_{10}[E][L_1L_1']$$

$$\frac{dTBA'}{dt}=K_6[T_1][BA']-K_{-6}[T_1BA']$$

$$\frac{dTAA'}{dt}=K_7[T_2][AA']-K_{-7}[T_2AA']$$

$$\frac{dTDC'}{dt}=K_8[T_1][DC']-K_{-8}[T_1DC']$$

$$\frac{dTCC'}{dt}=K_9[T_2][CC']-K_{-9}[T_2CC']$$

$$\frac{dE}{dt}=K_{-10}[EL_1L_1']-K_{10}[E][L_1L_1']+K_{11}[EL_1L_1']$$

$$\frac{dEL_1L_1'}{dt}=K_{10}[E][L_1L_1']-K_{-10}[EL_1L_1']-K_{11}[EL_1L_1']$$

$$\frac{dL_1L_{1-1}'L_{1-2}'}{dt}=K_{11}[EL_1L_1']-K_{12}[L_1L_{1-1}'L_{1-2}']+K_{-12}[L_1][L_{1-1}'][L_{1-2}']$$

$$\frac{dL_{1-1}'}{dt}=K_{12}[L_1L_{1-1}'L_{1-2}']-K_{-12}[L_1][L_{1-1}'][L_{1-2}']$$

$$\frac{dL_{1-2}'}{dt}=K_{12}[L_1L_{1-1}'L_{1-2}']-K_{-12}[L_1][L_{1-1}'][L_{1-2}']$$

Figure S28. The kinetic scheme of the reactions associated with the time-dependent concentration changes of each constituent in transient transition of CDN H→L→H is summarized in the above equations. Knowing the time-dependent concentration changes of each constituent, we computationally simulated the time-dependent concentration changes by using Matlab R2019b.

Kinetic equations of L_2'-triggered transient transition of CDN H→K→H shown in Figure 3A:

$$(1) AA' + BB' \underset{k_{-1}}{\overset{k_1}{\rightleftarrows}} AB' + BA'$$

$$(2) CC' + DD' \underset{k_{-2}}{\overset{k_2}{\rightleftarrows}} CD' + DC'$$

$$(3) L_1 + T_1 \underset{k_{-3}}{\overset{k_3}{\rightleftarrows}} L_1T_1$$

$$(4) T_2 + L_2 \underset{k_{-4}}{\overset{k_4}{\rightleftarrows}} T_2L_2$$

$$(5) T_2L_2 + L_2' \underset{k_{-5}}{\overset{k_5}{\rightleftarrows}} L_2L_2' + T_2$$

$$(6) T_1 + BA' \underset{k_{-6}}{\overset{k_6}{\rightleftarrows}} T_1BA'$$

$$(7) T_2 + AA' \underset{k_{-7}}{\overset{k_7}{\rightleftarrows}} T_2AA'$$

$$(8) T_1 + DC' \underset{k_{-8}}{\overset{k_8}{\rightleftarrows}} T_1DC'$$

$$(9) T_2 + CC' \underset{k_{-9}}{\overset{k_9}{\rightleftarrows}} T_2CC'$$

$$(10) E + L_2L_2' \underset{k_{-10}}{\overset{k_{10}}{\rightleftarrows}} EL_2L_2'$$

$$(11) EL_2L_2' \xrightarrow{k_{11}} E + L_2L_{2-1}'L_{2-2}'$$

$$(12) L_2L_{2-1}'L_{2-2}' \underset{k_{-12}}{\overset{k_{12}}{\rightleftarrows}} L_2 + L_{2-1}' + L_{2-2}'$$

Derivatives:

$$\frac{dAA'}{dt} = K_{-1}[AB'][BA'] - K_1[AA'][BB'] + K_{-7}[T_2AA'] - K_7[T_2][AA']$$

$$\frac{dBB'}{dt} = K_{-1}[AB'][BA'] - K_1[AA'][BB']$$

$$\frac{dAB'}{dt} = K_1[AA'][BB'] - K_{-1}[AB'][BA']$$

$$\frac{dBA'}{dt} = K_1[AA'][BB'] - K_{-1}[AB'][BA'] + K_{-6}[T_1BA'] - K_6[T_1][BA']$$

$$\frac{dCC'}{dt} = K_{-2}[CD'][DC'] - K_2[CC'][DD'] + K_{-9}[T_2CC'] - K_9[T_2][CC']$$

$$\frac{dDD'}{dt} = K_{-2}[CD'][DC'] - K_2[CC'][DD']$$

$$\frac{dCD'}{dt} = K_2[CC'][DD'] - K_{-2}[CD'][DC']$$

$$\frac{dDC'}{dt} = K_2[CC'][DD'] - K_{-2}[CD'][DC'] + K_{-8}[T_1DC'] - K_8[T_1][DC']$$

$$\frac{dT_1}{dt} = K_{-3}[T_1L_1] - K_3[T_1][L_1] + K_{-6}[T_1BA'] - K_6[T_1][BA'] + K_{-8}[T_1DC'] - K_8[T_1][DC']$$

$$\frac{dL_1}{dt} = K_{-3}[T_1L_1] - K_3[T_1][L_1]$$

$$\frac{dT_1L_1}{dt} = K_3[T_1][L_1] - K_{-3}[T_1L_1]$$

$$\frac{dT_2}{dt} = K_{-4}[T_2L_2] - K_4[T_2][L_2] + K_5[T_2L_2][L_2'] - K_{-5}[L_2L_2'][T_2] + K_{-7}[T_2AA'] - K_7[T_2][AA'] + K_{-9}[T_2CC'] - K_9[T_2][CC']$$

$$\frac{dL_2}{dt} = K_{-4}[T_2L_2] - K_4[T_2][L_2] + K_{-12}[L_2][L_{2-1}'][L_{2-2}'] - K_{12}[L_2L_{2-1}'L_{2-2}']$$

$$\frac{dT_2L_2}{dt} = K_4[T_2][L_2] - K_{-4}[T_2L_2] + K_{-5}[L_2L_2'][T_2] - K_5[T_2L_2][L_2']$$

$$\frac{dL_2'}{dt} = K_{-5}[L_2L_2'][T_2] - K_5[T_2L_2][L_2']$$

$$\frac{dL_2L_2'}{dt} = K_5[T_2L_2][L_2'] - K_{-5}[L_2L_2'][T_2] + K_{-10}[EL_2L_2'] - K_{10}[E][L_2L_2']$$

$$\frac{dTBA'}{dt} = K_6[T_1][BA'] - K_{-6}[T_1BA']$$

$$\frac{dTAA'}{dt} = K_7[T_2][AA'] - K_{-7}[T_2AA']$$

$$\frac{dTDC'}{dt} = K_8[T_1][DC'] - K_{-8}[T_1DC']$$

$$\frac{dTCC'}{dt} = K_9[T_2][CC'] - K_{-9}[T_2CC']$$

$$\frac{dE}{dt} = K_{-10}[EL_2L_2'] - K_{10}[E][L_2L_2'] + K_{11}[EL_2L_2']$$

$$\frac{dEL_2L_2'}{dt} = K_{10}[E][L_2L_2'] - K_{-10}[EL_2L_2'] - K_{11}[EL_2L_2']$$

$$\frac{dL_2L_{2-1}'L_{2-2}'}{dt} = K_{11}[EL_2L_2'] - K_{12}[L_2L_{2-1}'L_{2-2}'] + K_{-12}[L_2][L_{2-1}'][L_{2-2}']$$

$$\frac{dL_{2-1}'}{dt} = K_{12}[L_2L_{2-1}'L_{1-2}'] - K_{-12}[L_2][L_{2-1}'][L_{2-2}']$$

$$\frac{dL_{2-2}'}{dt} = K_{12}[L_2L_{2-1}'L_{2-2}'] - K_{-12}[L_2][L_{2-1}'][L_{2-2}']$$

Figure S29. The kinetic scheme of the reactions associated with the time-dependent concentration changes of each constituent in transient transition of CDN H→K→H is summarized in the above equations. Knowing the time-dependent concentration changes of each constituent, we computationally simulated the time-dependent concentration changes by using Matlab R2019b.

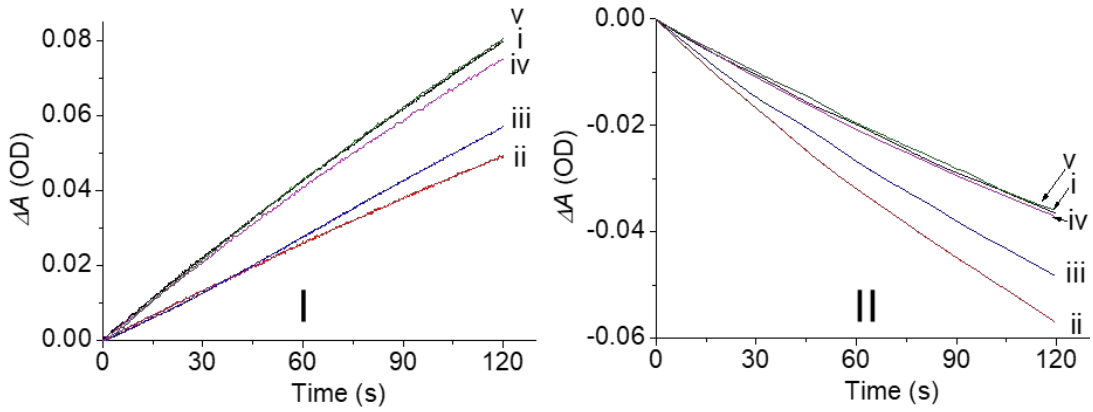

Figure S30. L_1' (8 μM)-triggered time-dependent absorbance changes generated by Panel I-the GOx/HRP cascade and Panel II-the LDH/NAD^+ cascade at time-intervals of the transient transition CDN H→L→H: (i) t = 0, (ii) after 50 min, (ii) after 100 min, (iv) after 200 min, (v) after 300 min.

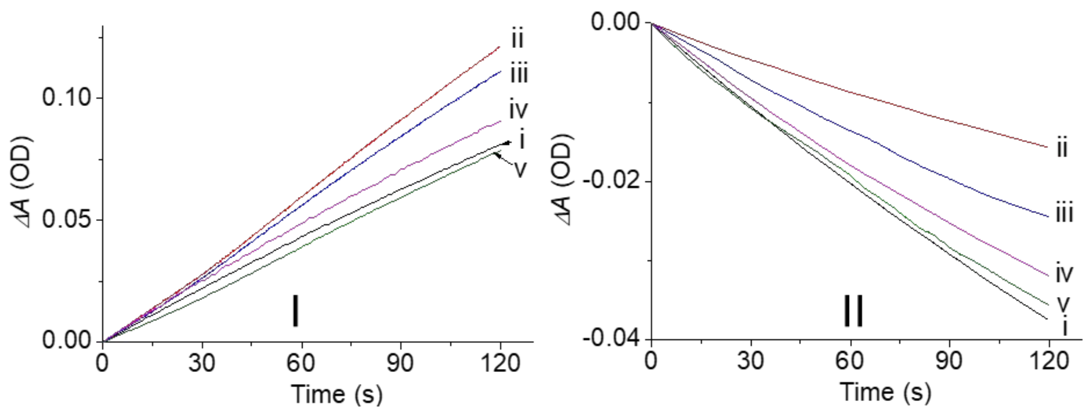

Figure S31. L_2' (8 μM)-triggered time-dependent absorbance changes generated by Panel I-the GOx/HRP cascade and Panel II-the LDH/NAD^+ cascade at time-intervals of the transient transition CDN H→K→H: (i) t = 0, (ii) after 50 min, (ii) after 100 min, (iv) after 200 min, (v) after 300 min.

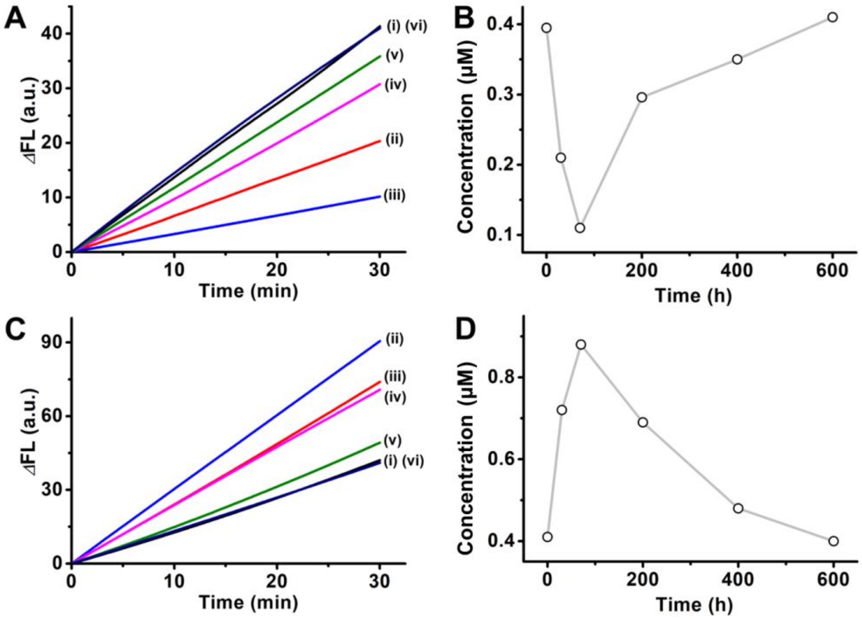

Figure S32. (A) Time-dependent catalytic activity changes of the DNAzyme reporter units associated with the constituents (CC′) at different time intervals during the dissipative transitions of CDN G→G_a→G, upon subjecting CDN G that includes the nicking enzyme, 0.0345 μM, to the fuel strand L_1', 6.0 μM. (B) Transient concentration changes of constituent CC′ upon subjecting the CDN G to L_1'. (C) Time-dependent catalytic activity changes of the DNAzyme reporter units associated with the constituents (CC′) at different time intervals during the dissipative transitions of CDN G→G_b→G, upon subjecting CDN G that includes the nicking enzyme, 0.0345 μM, to the fuel strand L_2', 6.0 μM. (D) Transient concentration changes of constituent CC′ upon subjecting the CDN G to L_2'.

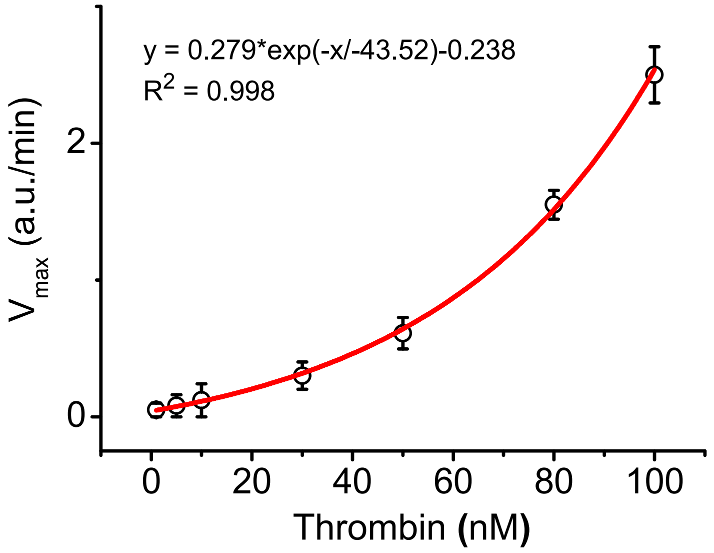

Figure S33. The calibration curve of sensing thrombin using the CDN G as a practical sensing platform.

Table S2. Rate constants derived from the computational simulation of L_1'-triggered transient transition of CDN X→Y→X shown in Figure 1A

k_1	1.26 μM^{-1} min^{-1}	k_4	0.10 μM^{-1} min^{-1}	k_7	6.23 μM^{-1} min^{-1}
k_{-1}	2.92 μM^{-1} min^{-1}	k_{-4}	5.65 μM^{-1} min^{-1}	k_{-7}	0.36 min^{-1}
k_2	15.21 μM^{-1} min^{-1}	k_5	7.63 μM^{-1} min^{-1}	k_8	1.27 min^{-1}
k_{-2}	9.5×10^{-5} min^{-1}	k_{-5}	0.086 min^{-1}	k_9	0.85 min^{-1}
k_3	12.68 μM^{-1} min^{-1}	k_6	6.82 μM^{-1} min^{-1}	k_{-9}	0.22 μM^{-2} min^{-1}
k_{-3}	8.33×10^{-4} min^{-1}	k_{-6}	0.035 min^{-1}		

Table S3. Rate constants derived from the computational simulation of L_2'-triggered transient transition of CDN X→Z→X shown in Figure 1A

k_1	1.26 μM^{-1} min^{-1}	k_4	0.35 μM^{-1} min^{-1}	k_7	7.23 μM^{-1} min^{-1}
k_{-1}	2.92 μM^{-1} min^{-1}	k_{-4}	4.53 μM^{-1} min^{-1}	k_{-7}	0.26 min^{-1}
k_2	15.21 μM^{-1} min^{-1}	k_5	7.63 μM^{-1} min^{-1}	k_8	1.27 min^{-1}
k_{-2}	9.5×10^{-5} min^{-1}	k_{-5}	0.086 min^{-1}	k_9	0.68 min^{-1}
k_3	12.68 μM^{-1} min^{-1}	k_6	6.82 μM^{-1} min^{-1}	k_{-9}	0.08 μM^{-2} min^{-1}
k_{-3}	8.33×10^{-4} min^{-1}	k_{-6}	0.035 min^{-1}		

Table S4. Rate constants derived from the computational simulation of L_1'-triggered transient transition of CDN O→M→O shown in Figure 2A

k_1	1.63 μM^{-1} min^{-1}	k_4	0.10 μM^{-1} min^{-1}	k_7	6.23 μM^{-1} min^{-1}
k_{-1}	2.86 μM^{-1} min^{-1}	k_{-4}	5.65 μM^{-1} min^{-1}	k_{-7}	0.36 min^{-1}
k_2	15.21 μM^{-1} min^{-1}	k_5	7.35 μM^{-1} min^{-1}	k_8	1.27 min^{-1}
k_{-2}	9.5×10^{-5} min^{-1}	k_{-5}	0.11 min^{-1}	k_9	0.85 min^{-1}
k_3	12.68 μM^{-1} min^{-1}	k_6	6.93 μM^{-1} min^{-1}	k_{-9}	0.22 μM^{-2} min^{-1}
k_{-3}	8.33×10^{-4} min^{-1}	k_{-6}	0.069 min^{-1}		

Table S5. Rate constants derived from the computational simulation of L_2'-triggered transient transition of CDN O→P→O shown in Figure 2A

k_1	1.63 μM^{-1} min^{-1}	k_4	0.35 μM^{-1} min^{-1}	k_7	7.23 μM^{-1} min^{-1}
k_{-1}	2.86 μM^{-1} min^{-1}	k_{-4}	4.53 μM^{-1} min^{-1}	k_{-7}	0.26 min^{-1}
k_2	15.21 μM^{-1} min^{-1}	k_5	7.35 μM^{-1} min^{-1}	k_8	1.27 min^{-1}
k_{-2}	9.5×10^{-5} min^{-1}	k_{-5}	0.11 min^{-1}	k_9	0.68 min^{-1}
k_3	12.68 μM^{-1} min^{-1}	k_6	6.93 μM^{-1} min^{-1}	k_{-9}	0.08 μM^{-2} min^{-1}
k_{-3}	8.33×10^{-4} min^{-1}	k_{-6}	0.069 min^{-1}		

Table S6. Rate constants derived from the computational simulation of L_1'-triggered transient transition of CDN H→L→H shown in Figure 3A

k_1	1.26 μM^{-1} min^{-1}	k_5	0.10 μM^{-1} min^{-1}	k_9	6.93 μM^{-1} min^{-1}
k_{-1}	2.92 μM^{-1} min^{-1}	k_{-5}	5.65 μM^{-1} min^{-1}	k_{-9}	0.069 min^{-1}
k_2	15.21 μM^{-1} min^{-1}	k_6	7.63 μM^{-1} min^{-1}	k_{10}	6.23 μM^{-1} min^{-1}
k_{-2}	9.5×10^{-5} min^{-1}	k_{-6}	0.086 min^{-1}	k_{-10}	0.36 min^{-1}
k_3	15.21 μM^{-1} min^{-1}	k_7	6.82 μM^{-1} min^{-1}	k_{11}	1.27 min^{-1}
k_{-3}	9.5×10^{-5} min^{-1}	k_{-7}	0.035 min^{-1}	k_{12}	0.85 min^{-1}
k_4	12.68 μM^{-1} min^{-1}	k_8	7.35 μM^{-1} min^{-1}	k_{-12}	0.22 μM^{-2} min^{-1}
k_{-4}	8.33×10^{-4} min^{-1}	k_{-8}	0.11 min^{-1}		

Table S7. Rate constants derived from the computational simulation of L_2'-triggered transient transition of CDN H→K→H shown in Figure 3A

k_1	1.26 μM^{-1} min^{-1}	k_5	0.35 μM^{-1} min^{-1}	k_9	6.93 μM^{-1} min^{-1}
k_{-1}	2.92 μM^{-1} min^{-1}	k_{-5}	4.53 μM^{-1} min^{-1}	k_{-9}	0.069 min^{-1}
k_2	15.21 μM^{-1} min^{-1}	k_6	7.63 μM^{-1} min^{-1}	k_{10}	7.23 μM^{-1} min^{-1}
k_{-2}	9.5×10^{-5} min^{-1}	k_{-6}	0.086 min^{-1}	k_{-10}	0.26 min^{-1}
k_3	15.21 μM^{-1} min^{-1}	k_7	6.82 μM^{-1} min^{-1}	k_{11}	1.27 min^{-1}
k_{-3}	9.5×10^{-5} min^{-1}	k_{-7}	0.035 min^{-1}	k_{12}	0.68 min^{-1}
k_4	12.68 μM^{-1} min^{-1}	k_8	7.35 μM^{-1} min^{-1}	k_{-12}	0.08 μM^{-2} min^{-1}
k_{-4}	8.33×10^{-4} min^{-1}	k_{-8}	0.11 min^{-1}		

Table S8. Quantification of the concentration and DNA labeling ratio of the purified enzyme-DNA conjugates by measuring the absorbance at 260 and 280 nm

**Enzyme**	**ε_{260} (M^{-1} cm^{-1})**	**ε_{280} (M^{-1} cm^{-1})**	**E_{405} (M^{-1} cm^{-1})**	**E_{450} (M^{-1} cm^{-1})**
GOx	168336	267200	-	22600
HRP	38000	-	100000	-
LDH	132175	186502	-	-
**DNA**	**ε_{260} (M^{-1} cm^{-1})**	**ε_{280} (M^{-1} cm^{-1})**		
B	385000	239229		
B′	283100	178975		
D′	281700	176752		
**Purified DNA-Enzyme**	**Ratio$_{(DNA:\ Enzyme)}$**			
B-GOx	1.01:1			
B′-HRP	1.03:1			
D′-LDH	0.98:1			

A_{260} (Enzyme-DNA) = ε_{260} (enzyme) × C (enzyme) + ε_{260} (DNA) × C (DNA) (1)

A_{280} (Enzyme-DNA) = ε_{280} (enzyme) × C (enzyme) + ε_{280} (DNA) × C (DNA) (2)

$$Ratio\left(\frac{DNA}{enzyme}\right) = \frac{c_{DNA}}{c_{enzyme}} \quad (3)$$
